# Cross-Scale Synthesis of Organic High-*k* Semiconductors Based on Spiro-Gridized Nanopolymers

**DOI:** 10.34133/2022/9820585

**Published:** 2022-01-12

**Authors:** Dongqing Lin, Wenhua Zhang, Hang Yin, Haixia Hu, Yang Li, He Zhang, Le Wang, Xinmiao Xie, Hongkai Hu, Yongxia Yan, Haifeng Ling, Jin'an Liu, Yue Qian, Lei Tang, Yongxia Wang, Chaoyang Dong, Linghai Xie, Hao Zhang, Shasha Wang, Ying Wei, Xuefeng Guo, Dan Lu, Wei Huang

**Affiliations:** ^1^Centre for Molecular Systems and Organic Devices (CMSOD), State Key Laboratory of Organic Electronics and Information Displays & Institute of Advanced Materials (IAM), Nanjing University of Posts & Telecommunications, 9 Wenyuan Road, Nanjing 210023, China; ^2^National Synchrotron Radiation Laboratory, Anhui Provincial Engineering Laboratory of Advanced Functional Polymer Film, CAS Key Laboratory of Soft Matter Chemistry, University of Science and Technology of China, Hefei 230026, China; ^3^School of Physics, State Key Laboratory of Crystal Materials, Shandong University, Jinan, Shandong 250100, China; ^4^Beijing National Laboratory for Molecular Sciences, National Biomedical Imaging Center, College of Chemistry and Molecular Engineering, Peking University, Beijing 100871, China; ^5^Frontiers Science Center for Flexible Electronics (FSCFE), MIIT Key Laboratory of Flexible Electronics (KLoFE), Northwestern Polytechnical University, Xi'an 710072, China; ^6^State Key Laboratory of Supramolecular Structure and Materials, College of Chemistry, Jilin University, 2699 Qianjin Avenue, Changchun 130012, China

## Abstract

High dielectric constants in organic semiconductors have been identified as a central challenge for the improvement in not only piezoelectric, pyroelectric, and ferroelectric effects but also photoelectric conversion efficiency in OPVs, carrier mobility in OFETs, and charge density in charge-trapping memories. Herein, we report an ultralong persistence length (*l*_p_ ≈ 41 nm) effect of spiro-fused organic nanopolymers on dielectric properties, together with excitonic and charge carrier behaviors. The state-of-the-art nanopolymers, namely, nanopolyspirogrids (NPSGs), are synthesized via the simple cross-scale Friedel-Crafts polygridization of A_2_B_2_-type nanomonomers. The high dielectric constant (*k* = 8.43) of NPSG is firstly achieved by locking spiro-polygridization effect that results in the enhancement of dipole polarization. When doping into a polystyrene-based dielectric layer, such a high-*k* feature of NPSG increases the field-effect carrier mobility from 0.20 to 0.90 cm^2^ V^−1^ s^−1^ in pentacene OFET devices. Meanwhile, amorphous NPSG film exhibits an ultralow energy disorder (<50 meV) for an excellent zero-field hole mobility of 3.94 × 10^−3^ cm^2^ V^−1^ s^−1^, surpassing most of the amorphous *π*-conjugated polymers. Organic nanopolymers with high dielectric constants open a new way to break through the bottleneck of efficiency and multifunctionality in the blueprint of the fourth-generation semiconductors.

## 1. Introduction

The fourth-generation semiconductors have been conceived as the cornerstone of intelligent flexible electronics, which would revolute the function and morphology of integrated circuits beyond Moore's law [1, 2]. In this aspect, organic semiconductors [3] hold promising advantages over atomically precise synthesis, structural diversity, multifunctional integration, and high biocompatibility, in promising applications of large-area, ultrathin, and wearable optoelectronic devices. However, even achieving significant development in organic light-emitting diodes [4], solar cells [5], and transistors [6, 7], organic semiconductors still suffer from low device performances, versus the rising hybrid perovskites [8] and inorganic counterparts. One of the fundamental origins for the dilemma situation is molecular nanoscale limitation that results in insufficient charge-screening behaviors and low dielectric constants (*k* = 2 ~ 4) [9]. Actually, dielectric constant is directly related to not only piezoelectric, pyroelectric, or ferroelectric effects [10] but also optoelectronic performances including charge separation efficiency in OPVs [11], carrier mobility in OFETs [12], and charge density in charge-trapping memories [13]. Particularly, high dielectric constant *k* ≥ 10 is a prerequisite of band-like carrier transport [14] and Wannier-Mott-like excitons [15] for high-performance inorganic semiconductors. However, it is quite difficult to improve *k* values only through carbon element, because the small electronic cloud volume leads to an intrinsically low polarization efficiency [16]. In consideration of carbon nanostructures [17*–*19] that play a key role in wave absorption materials with excellent dielectric properties, we design organic nanopolymers to propagate dipole polarization through effectively overcoming the limitation of molecular nanoscale.

**Figure 1:**
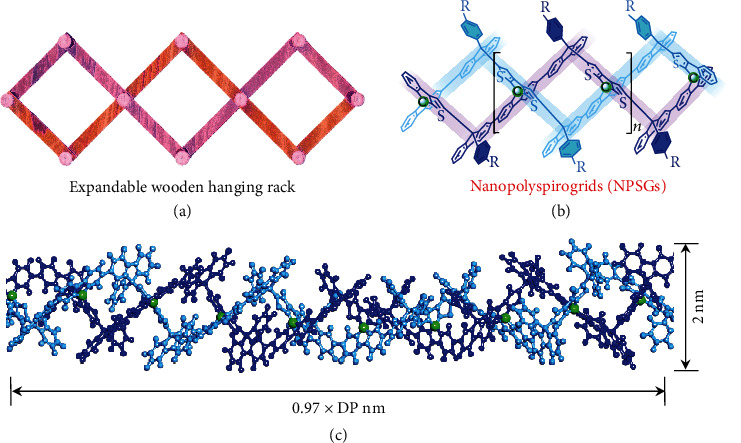
Nanopolyspirogrid (NPSG) models. (a) The wooden structure in an expandable hanging rack. (b, c) Molecular structures and 3D atomistic models of NPSG, respectively.

Cross-scale polygridization is a chemical platform to transform *π*-functional backbones into covalently ordered nanoarchitectures (with sizes of 10~100 nm) with promising applications in nanoelectronics and plastic electronics [17, 20], based on the extensibility and scalability of molecular nanogrid vertexes [2]. Generally, gridarene-based organic nanopolymers (also called polygrids) are one-dimensional covalent nanochains with intermediate main-chain rigidity between *π*-conjugated polymers and carbon nanotubes (CNT). However, these single-bond-linked polygrids [21] with relatively large conformational entropy exhibit a limited persistence length (*l*_p_ ≈ 16 nm) and thus disfavor covalent nanoscale ordering. Rhombus-type polygrids (RPGs), with backbone shapes resembling macroscopically expandable coat and cap racks (Figure 1(a)), offer an alternative installing paradigm to covalently lock conformational rotation and increase single-chain rigidity. In order to create double-stranded RPGs, we introduce orthogonal spiro-aromatic building blocks as diagonal vertexes of rhombus-type nanogrid (RGs) to construct spiro-nanopolymers. Figure 1(b) exhibits the structure of target nanopolyspirogrids (NPSGs) with blue-emitting terfluorenyl moieties linked by 3,3-dithiophenyl transverse beams at the spiro-sites (Figure 1(c)). Particularly, the dithiophenyl group ensures regular and high-efficient covalent linkages during Friedel-Crafts processes [22]. Herein, we synthesize spirodithiophenefluorene- (SDTF-) based spirodigrids (SDGs) and one-dimensional NPSGs with a length × width × height scale of 2 ~ 50 × 2 × 2.5 ~ 3.5 nm^3^, via the kinetically controlled spiro-polygridization of A_2_B_2_-type nanosynthons. An ultralong *l*_p_ = 41 nm is demonstrated by synchrotron radiation small-angle X-ray (SAXS) and static light scattering (SLS). Even with *π*-interrupted backbone, such ultrarigid NPSG exhibits a high dielectric constant (*k* = 8.43), an ultralow energy disorder (*σ* = 46.6 meV), and a highest carrier mobility (*μ* = 3.94 × 10^3^ cm^2^ V^−1^ s^−1^) in amorphous states, suggesting excellent electrical properties beyond *π*-conjugated polymers.

## 2. Results and Discussion

### 2.1. Synthesis and Characterizations of NPSG Nanochains

**Figure 2:**
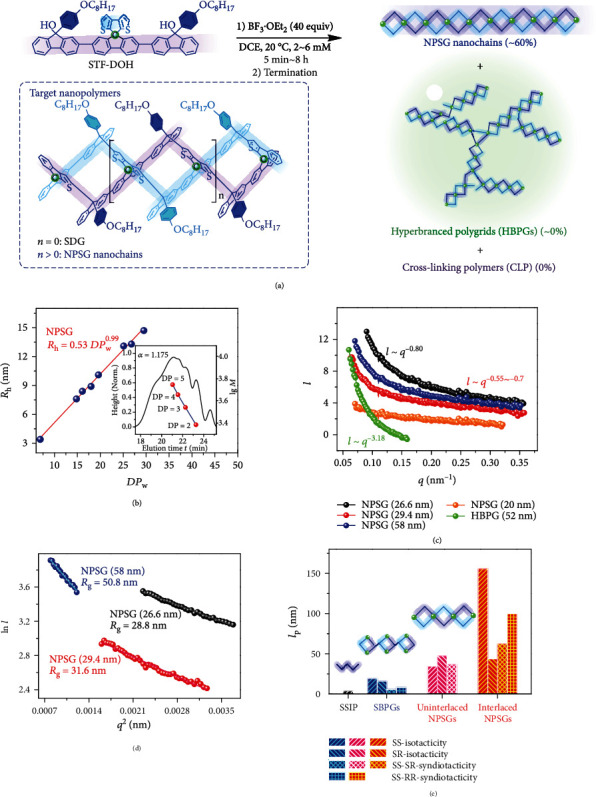
Synthesis and characterizations of NPSG. (a) Chemical equation of polygridization reaction. The likely structure of hyperbranched polygrid (HBPG), as byproducts, is provided in Figure S11. (b) The hydrodynamic radius (*R*_h_) relying on DP_w_. The calibration of NPSG oligomers (with the degree of polymerization DP = 2 ~ 5) from GPC spectra was also provided to calculate the Mark-Houwink exponent (*α*). (c) The scaling plots *I* ~ *q*^−*d*^ of NPSG and HBPG. The *l*_c_ of NPSG (in the brackets) are transformed from individual *R*_h_ or DP_w_ values. (d) The Quinier plots of NPSG with various *l*_c_ (in the brackets). The calculated radius of gyration (*R*_g_) is also provided. (e) The simulation of *l*_p_ of SSIP (orange), SBPGs (deep blue), and NPSGs (pink and red). The SSIP chain is defined as the single-chain segment of NPSG that removes all spiro-carbon atoms.

NPSGs can be synthesized via the Friedel-Crafts polygridization of SDTF-based terfluorenyl diol (STF-DOH), as one of A_2_B_2_-type nanosynthons (Figure 2(a)). Such nanosynthon consists of a 3,3-bithiophene-type benzenoid group (as B_2_-part) at the middle and two tertiary alcohols (as A_2_-part) at the ends, which enables the geometric matching between reactive 2-sites of thiophenes and 9-sites of fluorene-based carbocationic species for the formation of C-C bonds (Figure S9). As the fluorenol chirality does not influence the gridization/polygridization pathways due to S_N2_-type process [23], we performed the polygridization of STF-DOH in a chirality-mixed manner (mixed with *rac* and *meso*-configurations). In Figure 2(a), under the condition of BF_3_∙OEt_2_ (as acid catalyst [24]) in 1,2-dichloroethane (DCE) solvent and the dilute STF-DOH concentration (*C*_STF‐DOH_ = 2 mM), the linear soluble NPSG were afforded in ~60% yield. Further, elongating the reaction time from 0.5 min to 4~8 h increases the number-average degree of polymerization (DP_n_) of NPSG from 5.2 to 26.1 (Figure S10), corresponding to the average contour length (*l*_c_) of 25~30 nm. However, the longer polygridization time (13~22 h) results in the formation of hyperbranched polygrids (HBPGs), suggesting that the spiro-polygridization into NPSG should be in kinetic control. In addition, under the same polygridization time (4 h), increasing *C*_STF‐DOH_ to 6 mM can generate longer NPSG nanochains with *l*_c_ ≈ 58 nm, corresponding to the weight-average degree of polymerization (DP_w_) of ~65 (Figure S12). Nevertheless, higher *C*_STF‐DOH_ (12~16 mM) affords insoluble cross-linked polymers (CLP) in ~90% yield, whereas NPSG nanochains were not obtained.

The NPSG structures with *l*_c_ ≈ 2 ~ 50 nm were characterized by nuclear magnetic resonance (NMR), Fourier transform infrared (FT-IR), and Mass Spectrometry (MS). The MS characterization shows *m*/*z* = 2437.32 for SDG and *m*/*z* = 3497.45 for spirotrigrid (DP = 3 of NPSG), both of which are consistent with individual formula and isotopic distributions (Figure S29). Moreover, the oligomers with higher DP were also detected (Figure S32), including *m*/*z* = 4557.19 for DP = 4, *m*/*z* = 5618.55 for DP = 5, and *m*/*z* = 6679.29 for DP = 6, respectively, where each molecular weight difference between adjacent DP values (*m*/*z* ≈ 1060) is consistent with the structural feature. Through ^13^C NMR spectra (Figure S33), the absence of carbon signals at 54 ppm (the 9-position of phenylfluorenes) and 83.4 ppm (the 9-position of fluorenols) supports the occurrence of polygridization and the termination with A_1_B_1_ synthons, which eliminate hydroxyl groups at chain-ends (also confirmed by FT-IR spectra in Figure S34).

Further, the linear main-chain configuration of NPSG nanochains was demonstrated by gel permeation chromatography (GPC), dynamic light scattering (DLS), and SAXS. Through the analysis of GPC spectra (Figure 2(b)), the Mark-Houwink exponent (*α*) of NPSG oligomers (DP = 2 ~ 5) was calculated to be 1.175, which falls into the range of rod-like conformation [25] (*α* = 1.0 ~ 1.7) rather than branched configurations (*α* = 0.3 ~ 0.8) [26] (Figure S35). Meanwhile, through DLS characterization, the relationship of hydrodynamic radius (*R*_h_ = 7.6 ~ 14.7 nm) in *R*_h_ ~ DP_w_^0.99^ also reveals the rod-like conformation of NPSG nanochains, consistent with the visualization of atomic force microscopy (Figure S37). Via SAXS characterization, the rod-like conformation of NPSG nanochains is firmly confirmed by the absence of intrachain folding (deduced from Kratky plots *q*^2^*I* in Figure S38) and the ultralow mass fractal dimension (*d*) in scattering laws *I* ~ *q*^−0.8~−0.55^ (Figure 2(c)), where *I* and *q* are scattering intensity and vector, respectively. In contrast, the byproduct HBPG shows a high *d* = 3.18 with the presence of intrachain folding (Figure S38), as in agreement with spherical branching backbones (*d* ≈ 2 ~ 3) [27]. These results rule out the probability of branched structures (with significant amount) on NPSG nanochains. Deeply, the distance distribution function *P*(*r*) of NPSG (Figure S40) with a series of peaks at *r* = 2, 7.5, 12.5, and 18 nm suggests the covalently ordered periodic distribution on polygrid segments, unlike only a broad Gaussian-like pattern on random coil-like backbones [28].

The rigidity of NPSG nanochains was investigated via SLS and SAXS characterizations, as well as molecular dynamic simulations. We obtained the radius of gyration (*R*_g_) of 28.5 ~ 31.1 nm for the synthesized NPSG with *l*_c_ = 26 ~ 30 nm (Figure 2(d)), probably indicating *l*_p_ ≥ 30 nm. For longer NPSG nanochains with *l*_c_ ≈ 58 nm, we obtained *R*_g_ = 53.2 nm from SLS results and *R*_g_ = 50.8 nm from SAXS data (Figure S44). Based on the rigidity calculation [29], the average *R*_g_ = 52 nm and *l*_c_ = 58 nm afford *l*_p_ ≈ 41 nm, as beyond all soluble *π*-conjugated polymers [29, 30] (*l*_p_ = 3 ~ 20 nm, Figure S46). These results confirm the ultralong *l*_p_ property arising from synergistically double-bond-linked polygridization effects, as similar to DNA systems (*l*_p_ = 53 nm for double-stranded backbones and *l*_p_ = 1.5 nm for single-stranded main-chains) [31]. Further, through molecular dynamic simulation (Figure S47-S55), we evaluated *l*_p_ of all NPSG backbones (in an approximate Θ state, Figure 2(e)), including interlaced configurations (*l*_p_ = 155.8, 43.7, 62.5, and 100 nm for *SS*-isotacticity, *SR*-isotacticity, *SS*-*SR*-syndiotacticity, and *SS*-*RR*-syndiotacticity, respectively) and uninterlaced counterparts (*l*_p_ = 34.5 ~ 47.6 nm). The above *l*_p_ values are 1.8~27 times higher than that of single-bond-linked polygrids (SBPG) with *l*_p_ = 5.7 ~ 19.4 nm, revealing the effect of double-bond-linked spiro-polygridization on restricting conformational rotations of RG units (Figure S50). Further, *l*_p_ of NPSGs are 11~53 times longer than that of single-stranded *π*-interrupted polymers (SSIP) with *l*_p_ = 3.7 nm. In addition, the incorporation of ungridized structural defects also diminishes *l*_p_ to 5~10 nm (Figure S58-60), which reconfirms that our synthesized NPSG nanochains should have few ungridized structural defects. It is noted that such ultrarigid NPSG nanochains provide a unique molecular platform for covalent nanoscale ordering.

### 2.2. Dielectric Properties and Dipole Polarization of NPSG

**Figure 3:**
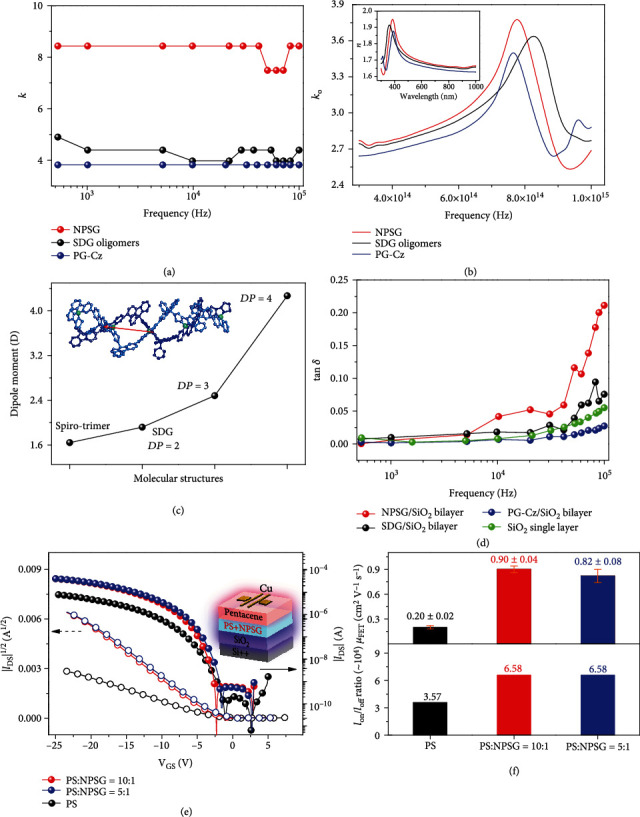
Dielectric features and OFET application of NPSG. (a) The dielectric constant (*k*) in the frequency range of 500~10^5^ Hz. (b) The optical dielectric constant (*k*_o_) and refractive index (*n*) of NPSG and other nanopolymer films. (c) The dependence of DP on the dipole moment, via quantum calculations. The dipole direction on the NPSG oligomer (DP = 4) was marked in red arrow. (d) The dielectric loss tangent (tan *δ*) of nanopolymer/SiO_2_ bilayer at 500~10^5^ Hz. (e) The transfer characteristics of top-gate/bottom-contact devices, where polystyrene (PS) and the doped NPSG serve as the dielectric layer; the pentacene serve as the semiconductor layer. The device structure is shown as well. (f) The field-effect carrier mobility (*μ*_FET_) and the on/off ratio (*I*_on_/*I*_off_), extracted from (e).

We probed the dramatic spiro-polygridization effect on the dielectric constant (*k*) at the frequency scope of 10^3^~10^5^ Hz (Figure 3(a)), through an impedance analyzer. In Figure 3(a), NPSG has an obviously higher *k* ≈ 8.43 than that of single-bond-linked polygrid PG-Cz (*k* ≈ 3.82) [32], as well as *π*-conjugated polythiophenes (*k* ≈ 3.75) [9] and polyfluorenes (*k* ≈ 2.75) [33]. This feature is completely distinguished from the porous effect of covalent organic frameworks [34] that reduce *k* value to 1.2~1.6 through large free volume. Furthermore, compared with NPSG nanochains, SDG oligomers exhibit the relatively lower *k* ≈ 4.39, suggesting that the covalently large-scale effect of NPSG can also be favorable to enhance polarization behaviors.

We further gain insight into the mechanism of polarization enhancement from the spiro-polygridization effect. Considering the frequency scope of 10^3^~10^5^ Hz, the high-*k* feature of NPSG likely originates from either electronic and atomic polarization or dipole polarization. In order to rule out electronic and atomic polarizations, we measured the optical dielectric constant (*k*_o_). For NPSG film, the refractive index (*n*) of 1.66~1.81 and the extinction coefficient (*K*) of 0~0.008 are transformed into *k*_o_ = 2.77 ~ 3.27at the high frequency of 3 × 10^14^ ~ 7 × 10^14^ Hz (Figure 3(b)), as comparable to SDG oligomers (*n* = 1.77 ~ 1.66 and *k*_o_ = 2.74 ~ 3.13) and PG-Cz (*n* = 1.75 ~ 1.63 and *k*_o_ = 2.64 ~ 3.08). The difference between *k*_o_ and *k* is obviously observed in polygrid backbones, especially for NPSG with *k* − *k*_o_ = 5.16 ~ 5.66. This feature is distinct from *π*-conjugated polyfluorenes (*n* ≈ 1.6 and *k*_o_ ≈ 2.5) [35] and polythiophenes (*n* ≈ 1.92 and *k*_o_ ≈ 3.6) [36] with *k* ≈ *k*_o_. Therefore, the high-*k* feature of NPSG nanochains is probably derived from the enhancement in dipole polarization.

The enhancement in dipole polarization was also confirmed by the theoretical calculations of dipole moment. In the NPSG oligomer models (Figure 3(c) and S69), we found that the dipole direction is roughly along the main-chain. Deeply, the dipole moment is gradually increased from 1.643 D (ungridized spiro-trimer), 1.919 D (SDG, DP = 2), 2.482 D (DP = 3) to 4.269 D (DP = 4). As a result, increasing the NPSG length enables to accumulate dipole moment. For longer NPSG nanochains, the ultralong persistence length allows the covalently ordered arrangement of STDF groups in rod-like main-chain conformation, versus coil-like backbones with disordered arrangements. In this case, such ultrarigid feature is favorable to maintain the dipole orientation (along the main-chain) and accumulatively enhance the dipole moment when increasing the DP. On this basis, the tiny pores of RG units (the sizes of 0.4 nm^2^) on NPSG nanochains (even with few amount of structural defects, the electric properties were not influenced, see the following) suppress the effect of free volume on reducing the *k* value, which ensures the enhancement of dipole polarization in longer NPSG nanochains.

In addition, we examined the dielectric loss tangent (tan *δ*) of NPSG-containing films. In Figure 3(d), the NPSG/SiO_2_ bilayer possesses tan *δ* = 0.05 ~ 0.20 at 10^4^~ 10^5^ Hz, which are obviously higher than that of the single SiO_2_ layer with tan *δ* = 0.008 ~ 0.055, the SDG/SiO_2_ bilayer with tan *δ* = 0.02 ~ 0.08, the PG-Cz/SiO_2_ bilayer with tan *δ* = 0.006 ~ 0.027, and polyfluorene/SiO_2_ bilayer with tan *δ* = 0.01 ~ 0.03 (Figure S70). These results suggest that adding NPSG layer obviously increases the leakage current and affords higher conductivity for dielectric layers [37], which is also consistent with the nature of higher dielectric constant.

We further fabricated pentacene OFETs by blending polystyrene- (PS-) based dielectric layer with NPSG to confirm the effect of high-*k* feature. Figure 3(e) exhibits the transfer characteristics when using the NPSG-doped dielectric layer. The SiO_2_ layer is also used to maintain the low leakage current of OFET devices. Transistors with blending NPSG in the 1 : 10~1 : 5 ratio increase the drain current (*I*_DS_) by 5 times in the saturation regimes at the negative gate voltage (*V*_GS_) of -20~-25 V. Correspondingly, the field-effect mobility (*μ*_FET_) of pentacene layer is obviously increased from 0.20 ± 0.02 cm^2^ V^−1^ s^−1^ (undoped PS dielectric layer) to 0.90 ± 0.04 cm^2^ V^−1^ s^−1^ (PS : NPSG = 10 : 1) and 0.82 ± 0.08 cm^2^ V^−1^ s^−1^ (PS : NPSG = 5 : 1, Figure 3(f)), which are higher than *μ*_FET_ = 0.1 ~ 0.3 cm^2^ V^−1^ s^−1^ in common pentacene OFET devices [12, 38, 39]. Meanwhile, the on/off ratio (*I*_on_/*I*_off_) is increased from 3.57 × 10^4^ (undoped PS layer) to 6.58 × 10^4^ (NPSG-doping layer). Considering that the film morphology with larger roughness (Figure S71) should not enhance the carrier mobility, the high-*k* property of NPSG may be partly attributed to the improved OFET performances, because of the excellent screening effect on diminishing the interfacial coulomb impurities [39]. Further, doping high-*k* NPSG in the PS layer also results in the decreased threshold voltage from -5.37 V to -4.88~-4. 78 V, which is potential to develop OFET with lower operational voltage.

### 2.3. Excitonic Emission and Charge Carrier Transport in NPSG

To examine the effect of ultralong *l*_*p*_ feature and covalently large scale on the optoelectronic behaviors of NPSG (DP_n_ ≈ 26), we also conducted a series of measurements such as ultraviolet-visual absorption (UV), photoluminescence (PL), carrier mobility, and single-molecular conductance.

**Figure 4:**
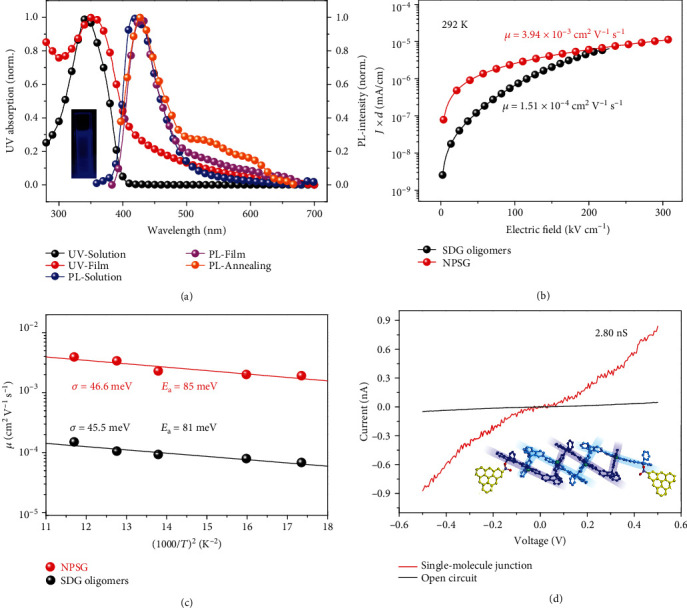
Optoelectronic features and carrier mobility of NPSG. (a) UV-vis absorbance and PL spectra of NPSG in solution (CHCl_3_ solvent), pristine, and annealing film. (b) The space-charge limit current density (*J*) of NPSG (red) and SDG (black) at 292 K, along with their hole mobility. The *d* is the thickness of the film. (c) The dependence of temperature on hole mobility for NPSG (red) and SDG (black), along with the energy disorder (*σ*) and activation energy (*E*_a_). (d) Current-voltage curves of single-chain NPSG and single-molecular devices.

For UV spectra in solution (Figure 4(a)), NPSG nanochains have an absorption peak at 343 nm and a wide shoulder peak at 368~370 nm. Although only with an efficient *π*-conjugation length on terfluorenes, NPSG possesses an optical bandgap (*E*_g_) of ~3.11 eV, closely equivalent to that of tetrafluorenyl moieties (*E*_g_ = 3.10 eV) [32]. Moreover, even on *π*-interrupted backbones, *E*_g_ is still gradually decreased from 3.33 eV to 3.11 eV (Figure S72) when increasing the backbone length from ~2 nm (SDG) to >20 nm (NPSG). These results indicate that *π*-interrupted NPSG still benefits for the excitonic delocalization. For PL spectra in solution, NPSG shows a deep-blue emission at 415 nm with a lifetime of ~1.07 ns (Figure S73) and a larger Stokes shift of ~68 nm, totally different from ladder-type polymers [40] with small Stokes shifts of 5~25 nm. In the film state, NPSG still maintains a blue emission at ~430 nm without the obvious *g*-band defect emission, even after annealing under 260°C temperature at air atmosphere (Figure S74). Thus, NPSG-based wide-bandgap nanomaterials enable to compete with conjugated polyfluorenes [41], ZnO nanoparticles [42], and CNT (without emission) [43].

The carrier mobility of amorphous NPSG film (Figure S75) was investigated through the space-charge limited current (SCLC) method. Under the temperature of 292 K (Figure 4(b)), NPSG exhibits an outstanding zero-field hole mobility *μ* = 3.94 × 10^−3^ cm^2^ V^−1^ s^−1^. To our best knowledge, such zero-field mobility is the highest value (Figure S77) among all fully *π*-interrupted molecules [44] with *μ* = 10^−7^ ~ 10^−4^ cm^2^ V^−1^ s^−1^. Particularly, it is unexpected that the zero-field hole mobility of *π*-interrupted NPSG can be even higher than that of amorphous *π*-conjugated polymers including polythiophenes [45] (*μ* ≈ 1 × 10^−3^ cm^2^ V^−1^ s^−1^) and polyfluorenes [46, 47] (*μ* ≈ 10^−7^ ~ 10^−4^ cm^2^ V^−1^ s^−1^). In contrast, amorphous SDG oligomers exhibit an evidently lower current density under the same electric field and possess a lower mobility *μ* = 1.51 × 10^−4^ cm^2^ V^−1^ s^−1^, as an order of magnitude lower than that of NPSG. In addition, versus NPSG, the SCLC curve of SDG shows a stronger electric-field-dependent feature in current density (Figure S79), which suggests the presence of more deep-traps [48]. These results support the effect of covalently large scale on improving charge transport, which was ever hidden by the effective *π*-conjugation length and was diminished by the short persistence length in conventional organic semiconductors [15].

Further, we studied the energy disorder and activation energy of amorphous NPSG film to uncover the physical mechanism during carrier transport. In Figure 4(c), even under low temperature at 240 K, NPSG still displays a high carrier mobility of *μ* = 1.93 × 10^−3^ cm^2^ V^−1^ s^−1^ and shows a relatively weak temperature dependence on hole transport. According to the Gaussian disorder model [49], NPSG displays an ultralow energy disorder (*σ*) of 46.6 meV, which is far lower than that of amorphous *π*-conjugated polymers (Figure S81) with obviously shorter *l*_p_, including polyfluorenes [46] with *σ* ≈ 100 meV and *l*_p_ ≈ 6 ~ 9 nm, poly-*p*-phenylene vinylenes [50] with *σ* ≈ 160 meV and *l*_p_ ≈ 7 nm, and polythiophenes [45, 49] with *σ* ≈ 70 meV and *l*_p_ ≈ 3 ~ 10 nm. Meanwhile, the activation energy (*E*_a_) of amorphous NPSG film is 85 meV that can even fall into the semicrystalline range [7] of polythiophenes with *E*_a_ = 50 ~ 100 meV, as lower than other amorphous conjugated polymers [7] with *E*_a_ = 150 ~ 250 meV. These results confirm the ultralong persistence length effect that efficiently suppresses the defect scattering and trapping levels to decrease the width of density-of-states (DOS). Deeply, through quantum calculation (Figures S82-S84), the highest occupied molecular orbital (HOMO) and its degenerated orbital levels for hole transport are distributed on both dithiophenyl and monofluorenyl planes of STDF moieties, which are arranged in covalently well-ordered states for the narrow DOS feature. In addition, the ultralow energy disorder is similarly observed in SDG oligomers (*σ* = 45.5 meV and *E*_a_ = 81 meV). These results reveal that even with structural defects in low amount (such as ungridized or branching defects that can increase conformational entropy), the electric properties of our synthesized NPSG nanochains are not influenced obviously. Moreover, the above ultralow *σ* level confirms the theoretical observation of gridization effect on lowering reorganization energy [51]. Notably, the ultralow energy disorder can be integrated with high-*k* feature perfectly, which cannot be possible in other high-*k* organic polymers via adding polar groups on flexible alkyl chains [52]. High-*k*-based NPSG with ultralow energy disorder offers a potential approach to design advanced donor/acceptor materials in the application of OPVs.

Single-molecular diode was also applied to investigate the effect of *π*-interrupted NPSG backbone on the single-chain conductance (*G*). The NPSG single-chain (with the length of 15~20 nm) was covalently linked by graphene segments (as the electrodes), and then, the voltage between two graphene segments was applied to obtain the current (*I*)-voltage (*V*) curve. Generally, the conjugated planar polymer such as polyporphyrin nanochains exhibits the relatively high conductance *G* = 10^2^ ~ 10^4^ nS [17]. In contrast, NPSG single-chain exhibits the highest conductance up to *G* = 2.8 nS (in Figure 4(d)), which is at least two orders of magnitude lower than that of conjugated polymer chains. This result is consistent with the feature of *π*-interrupted main-chain that disfavors the *π*-electronic delocalization and transport behaviors. Even so, the *I*-*V* curve of NPSG single-chain exhibits the approximately symmetric pattern where the conductance is enhanced with increasing the bias from 0 to 0.5 V, reflecting the feature of typically molecular diode. Thus, NPSG nanochains would be favorable for the semiconducting molecular nanowires or nanoelectronics.

## 3. Conclusion

In summary, a state-of-the-art ultrarigid spiro-nanopolymer has been created to explore the effect of covalent nanoscale ordering on the ultralow energy disorder and high dielectric constant. Conformational entropy has been effectively suppressed by spiro-polygridization to achieve the recording rigidity with *l*_p_ ≈ 41 nm. As a result, the dipole polarization is enhanced by large covalent nanoscale effect to give the higher dielectric constant (*k* = 8.43), as approximate to GaN (*k* ≈ 9). Furthermore, conformational defects and trapping levels are reduced, which gives an excellent hole mobility (*μ* = 3.94 × 10^−3^ cm^2^ V^−1^ s^−1^). To our best knowledge, these results surpass all amorphous *π*-conjugated polymer semiconductors reported in the literatures. Our polygridization strategy probably become the powerful cross-scale chemistry to hierarchically modulate the nature of organic nanopolymers that would open a new door to challenge the physical optoelectronic extreme and to make recording performance of semiconducting device. By means of such polygridization-type molecular integration technology (MIT), organic nanopolymer semiconductors would become potential candidates of the fourth-generation semiconductors with the feature of high-performance, multifunctionality, intelligence, and ubiquity to fulfill the requirement of flexible electronics, nanoelectronics, and organic intelligence.

## 4. Materials and Methods

### 4.1. Materials

The detailed synthetic procedures of substrate synthons and NPSG chains are described in the Supplementary Information files.

### 4.2. Gel Permeation Chromatography (GPC)

The GPC characteristics were conducted on a HP1100 HPLC system possessing 7911GP-502 and GPC columns using polystyrenes as the standard and tetrahydrofuran (THF) as the eluent at a flow rate of 1.0 ml/min at 25°C. The concentrations of NPSG solutions were about 0.8 mg/ml.

### 4.3. Dynamic Light Scattering (DLS) and Static Light Scattering (SLS)

The DLS characterizations of NPSG solution (CHCl_3_ as a solvent) were determined by a Brookhaven Instrument (ZetaPALS) to obtain *R*_h_. The operating wavelength of light source is 632.8 nm. The SLS measurements were performed to calculate *R*_g_, via ALV/CGS-3 light-scattering spectrometer that is equipped with an ALV/LSE-7004 multiple-*τ* digital correlator. All of the operating wavelength are 632.8 nm for light source.

### 4.4. Small-Angel X-Ray Scattering (SAXS)

The scattering datum was provided via the synchrotron radiation SAXS from Shanghai Synchrotron Radiation Facility. The distance from the sample cell (with mica windows in the path length of 1.5 mm) to the detector is 1 m. The wavelength of X-ray is 1.2 Å. The collected scattering vectors *q* ranges from 0.01 to 0.4 nm^−1^. The substrate of the solvent (toluene) from the solution scattering was performed before the analysis. The data were collected with the exposure time of 2 s, the acquired period of 2.01 s, and the images of 10. The measured NPSG solutions were 0.5~2 mg/ml in toluene solvents.

### 4.5. Molecular Dynamic Simulations

The molecular models of NPSG-based spirotrigrids were constructed and calculated via the Forcite plus module in the software Materials Studio. The geometry optimization was performed based on the SMART algorithm (as the cascade Steepest Descent-ABNR-Quasi-Newton algorithm). The simulations of conformational motions of spirotrigrids were based on the NVT ensemble (in vacuum), the pcff forcefield, the time-step of 0.2 fs, and the total time of 100 ps.

### 4.6. The Calculation of Dielectric Constant (*k*) of NPSG

The diode device structure Cu/SiO_2_/NPSG/Si (n^+^) was fabricated to measure the total capacitance *C*_i_, which is transformed to *k* via the equation *ε*_0_/C_i_ = *d*_s_/*k*_*s*_ − *d*/*k*, where *d*_s_, *k*_*s*_, and *ε*_0_ are defined as the layer thickness, the dielectric constant of SiO_2_ (*k*_*s*_ = 4.0) [9], and the permittivity of vacuum (8.85 × 10^−12^ F/m), respectively. The NPSG layer (from the DCE solution with the NPSG concentration of 5 mg/ml) was spin-coated on the SiO_2_ layer, which was followed by annealing under the conditions of a vacuum environment, 80°C and 30 min. The procedure and conditions of spin-coating other polymers/oligomer films were the same as those of NPSG. *C*_i_ was measured via an impedance analyzer (IM3533), through a frequency sweep of 500~100000 Hz and a bias of 0.1 V. The thicknesses of the NPSG film were measured by ellipsometry (J.A. Woollam RC2). The dielectric loss (tan *δ*) of NPSG-based diode device (bilayer of NPSG and SiO_2_) was afforded via impedance analyzer at 10^3^~10^5^ Hz. The optical dielectric constant (*k*_o_) of NPSG, in the high-frequency range of 3 × 10^14^ ~ 8 × 10^14^ Hz, was calculated via the equation *k*_o_ = *n*^2^–*K*^2^, where *n* and *K* are defined as the refractive index and the extinction coefficient, respectively. Both were afforded via the ellipsometry characterization.

### 4.7. OFET Device

We used a heavily doped *n*-type Si wafer (as the control gate) containing a 50 nm-thick SiO_2_ layer, which serves as the control dielectric layer. The surface of Si wafer is carefully washed by acetone, ethanol, and deionized water in 20 minutes, via ultrasonic cleaning, which is followed by blowing with nitrogen atmosphere. Then, these wafers were dried over in the vacuum atmosphere (under the temperature of 120°C and 30 minutes). The polystyrene or the mixed polystyrene and NPSG samples were dissolved in 1,2-dichloroethane and then spin coated on SiO_2_ as a polymer dielectric layer. The semiconductor layer of 50 nm thick pentacene was deposited onto the PS layer or mixed PS and NPSG layer, under the thermal vacuum evaporation method at 5 × 10^4^ Pa. The Cu film with the thickness of 100 nm, serving as the source and drain electrodes, was thermally evaporated through a shadow mask. The channel length (*L*) and width (*W*) were 150 and 1500 *μ*m, respectively. All of the devices were synchronously fabricated at the same conditions and characterized in a shielding box in ambient air (RH = 2%), using a Keithley 2636B semiconductor parameter analyzer.

### 4.8. Ultraviolet-Visual Absorption (UV-vis) and Photoluminescence Spectra (PL)

The solution was prepared under the concentration of 10^−2^ mg/ml in CHCl_3_ or DCE solvent. The film was spin-coated from the solution (DCE solvent, the concentration of 8 mg/ml) under the 800 rad/s. The UV spectra (LAMBDA 35) were used to characterize the photophysical properties of their ground states. The PL spectra (RF-6000 Plus) were obtained to study the excitonic behaviors of their excited states.

### 4.9. Measurement of Space-Charged-Limited Current for Carrier Mobility

The hole-only device with the structure of ITO/PEDOT:PSS/NPSG/Au was fabricated according to the literature [49]. The poly(3,4-ethylenedioxythiophene)-doped poly(styrene sulfonic acid) (PEDOT : PSS) layer and NPSG layer (or SDG oligomers, both in chlorobenzene solvent) were prepared via spin-coating at the spin rate 7000 RPM and annealing under 140°C (10 min). For the space-charged-limited current measurement, injecting the charge carriers into the thin-film active layer was performed under the DC voltage, through a source measure unit (SMU) Keithley (Model 2612B) that also records the currents under different voltage conditions. The calculation of zero-field carrier mobility is based on the *J* ~ *E*^2^ region in the SCLC curve (where *J* and *E* are the current density and electric field, respectively) to ensure the low deviation.

### 4.10. Single-Molecular Electronic Device

A new dash-line lithographic (DLL) method, referred to the literature [53], was used to fabricate the single-molecular electronic device. For the linkage of a polymeric single-chain via ester linkage, NPSG were dissolved in dichloromethane with the concentration ~10^−4^ M, followed by adding graphene devices and carbodiimide dehydrating/activating agent. After 2 days, the NPSG device was taken out from solution. Such device was washed through copious acetone and ultrapure water solvent and then was dried under N_2_ atmosphere. We used an Agilent 4155C semiconductor characterization system and a Karl Suss (PM5) manual probe station to measure the current (*I*)-voltage (*V*) curve under the ambient atmosphere.

## Data Availability

All data is available in the main text or the Supplementary Information. The data are available from the corresponding author on reasonable request.

## Supplementary Materials

Supplementary Materials Figure S1: synthetic routes of NPSG. Figure S2: the nanoarchitectures of rhombus-type grids (RGs) and single-bond-linked polygrids (SBPGs). Figure S3: the diastereoisomers of RGs. Figure S4: the nanoarchitectures of spirodigrids (SDGs), and single-bond-linked rhombus-type digrids (SRDGs). Figure S5: the diastereoisomers of SDG. Figure S6: the NPSG chains with various tacticity and interlaced or uninterlaced types. Figure S7: the helical backbones of SS-isotactic and RR-isotacticity NPSG backbones (all in interlaced states). Figure S8: the retrosynthetic analysis of NPSG. Figure S9: the geometric matching model of STF-DOH intermediates (carbon cationic species). Figure S10: the reaction time (*t*_r_) dependence of polygridization. Figure S11: the likely structure of hyperbranched polymer (HBPG) from the spiro-polygridization. Figure S12: the *C*_STF‐DOH_ dependence of polygridization. Figure S13: the GPC spectra of spirodigrids (a) and the oligomers of NPSG (b). Table S1: the relationship between elution time and molar mass of NPSG oligomers. Table S2: the elution time of NPSG with individual molar mass (extrapolated via its calibration equation). Figures S14, S15, S16, S17, S18, S19, S20, S21, S22, S23, S24, S25, and S26: the GPC spectra of NPSG. Figures S27 and S28: the hydrodynamic radius (*R*_h_) of NPSG. Figure S29: MS spectra of SDG (a) and spirotrigrid (b). Figure S30: MS spectra simulation of ungridized defective SDG structures. Figure S31: MS spectra simulation of ungridized defective spriotrigrid structures. Figure S32: MS spectra of NPSG oligomers. Figure S33: ^13^C NMR spectra of STF-DOH, STF-DOH-2H (dehydroxylated STF-DOH, as the byproduct) SDG, and NPSG. Figure S34: FT-IR spectra of NPSG. Figure S35: the relationship between Mark-Houwink exponent *α* and persistence length (in *θ* or stretched state). Figure S36: the hydrodynamic radius models of the NPSG chain (DP = 8 as an example). Figure S37: the NPSG single-chain image from atomic force microscopy (AFM), with the height profiles (in blue region). Figure S38: the Kratky plots of NPSG (not green lines) and BSG (green lines, in the green region of branch with chain folding) in toluene. Figure S39: the special mass fractal dimension of NPSG and other polymers. Figure S40: the distance distribution function *P*(*r*) of NPSG with various average chain lengths. Figure S41: the distance distribution function *P*(*r*) of HBPG. Figures S42, S43, and S44: the radius of gyration of NPSG with various contour lengths. Figure S45: the radius of gyration of HBPG with average chain length *L* ≈ 52.2 nm (transformed from *R*_h_ values). Figure S46: the NPSG position (marked in red stars) in the statistical graph of persistence length and monomeric sizes. Figure S47: the models of calculating persistence length. Figures S48, S49, S50, S51, S52, S53, S54, S55, and S56: the conformational relaxations of spirotrigrids and STRG with various tacticies. Figure S57: the conformational relaxation of single-stranded ungridized oligomers whose polymers originate from the deletion of the spiro-carbon atoms on NPSG backbones (SSIP). Figure S58: the ungridized defective NPSG oligomer model. Figures S59 and S60: the conformational relaxations of ungridized defective spirotrigrids with various tacticities. Figures S61, S62, S63, and S64: the capacity of SiO_2_ layer, NPSG-based diode device, SDG oligomer-based diode device, and PG-Cz-based diode device, respectively. Figures S65, S66, and S67: the refractive index (*n*) and the extinction coefficient (*K*) of NPSG, SDG, and PG-Cz, respectively, in the wavelength range of 300~1000 nm. Figure S68: the optical dielectric constant (*k*_o_) of NPSG, SDG oligomers, and PG-Cz in the high-frequency range of 3 × 10^14^ ~ 1 × 10^15^ Hz. Figure S69: the direction of dipole moment (marked in blue lines) of spiro-trimer, SDG, spirotrimer, and spirotetramer. Figure S70: the dielectric loss tangent (tan *δ*) of polyfluorene/SiO_2_ bilayer. Figure S71: AFM image of polystyrene (PS) and polystyrene-NPSG doping films. Table S3: the photophysical parameters of NPSG solutions. Figure S72: UV-PL spectra of STF-DOH and NPSG (in CHCl_3_ solvent) with various DP_n_. Figure S73: transition decay and lifetimes of NPSG in CHCl_3_ solvent. Figure S74: photophysical properties of STF-DOH, SDG, and NPSG. Table S4: the photophysical parameters of NPSG film (spin-coated from DCE solution). Figure S75: the X-ray diffraction (XRD) of spin-coated NPSG film. Figure S76: the DSC characterization of NPSG. Figure S77: the zero-field carrier mobility of various organic small molecules and polymers in amorphous state. Figure S78: the dependence of space-charged-limited current density (*J*) × (film thickness of NPSG/SDG) *d* on the electric field, under the temperature of 240 K. Figures S79 and S80: fitting of SCLC curve of NPSG and SDG under the temperature of 293 K and 240 K, respectively. Figure S81: the energy disorder of various organic small molecules and polymers in amorphous state. Figures S82, S83, and S84: the molecular orbital distribution of SDG, spirotrigrid, and spirotetragrid, respectively. Figure S85: a macroscopic pattern of NPSG-based molecular electronic device. Figure S86: the SEM imagine of molecular electronic device. Figures S87, S88, S89, S90, S91, S92, S93, S94, S95, S96, S97, and S98: ^1^H and ^13^C NMR spectra of substrates, intermediates, and NPSGs.
