# Supplementary material for: Cross-Scale Synthesis of Organic High-k Semiconductors Based on Spiro-Gridized Nanopolymers
Source: Research (Wash D C). 2022 Jan 12;2022:9820585. doi: 10.34133/2022/9820585 (PMC8777471; doi:10.34133/2022/9820585)
Supplement: Supplementary Materials — Figure S1: synthetic routes of NPSG. Figure S2: the nanoarchitectures of rhombus-type grids (RGs) and single-bond-linked polygrids (SBPGs). Figure S3: the diastereoisomers of RGs. Figure S4: the nanoarchitectures of spirodigrids (SDGs), and single-bond-linked rhombus-type digrids (SRDGs). Figure S5: the diastereoisomers of SDG. Figure S6: the NPSG chains with various tacticity and interlaced or uninterlaced types. Figure S7: the helical backbones of SS-isotactic and RR-isotacticity NPSG backbones (all in interlaced states). Figure S8: the retrosynthetic analysis of NPSG. Figure S9: the geometric matching model of STF-DOH intermediates (carbon cationic species). Figure S10: the reaction time (tr) dependence of polygridization. Figure S11: the likely structure of hyperbranched polymer (HBPG) from the spiro-polygridization. Figure S12: the CSTF‐DOH dependence of polygridization. Figure S13: the GPC spectra of spirodigrids (a) and the oligomers of NPSG (b). Table S1: the relationship between elution time and molar mass of NPSG oligomers. Table S2: the elution time of NPSG with individual molar mass (extrapolated via its calibration equation). Figures S14, S15, S16, S17, S18, S19, S20, S21, S22, S23, S24, S25, and S26: the GPC spectra of NPSG. Figures S27 and S28: the hydrodynamic radius (Rh) of NPSG. Figure S29: MS spectra of SDG (a) and spirotrigrid (b). Figure S30: MS spectra simulation of ungridized defective SDG structures. Figure S31: MS spectra simulation of ungridized defective spriotrigrid structures. Figure S32: MS spectra of NPSG oligomers. Figure S33: 13C NMR spectra of STF-DOH, STF-DOH-2H (dehydroxylated STF-DOH, as the byproduct) SDG, and NPSG. Figure S34: FT-IR spectra of NPSG. Figure S35: the relationship between Mark-Houwink exponent α and persistence length (in θ or stretched state). Figure S36: the hydrodynamic radius models of the NPSG chain (DP = 8 as an example). Figure S37: the NPSG single-chain image from atomic force microscopy (AFM), with the he [file 9820585.f1.docx]

**Supporting Information**

**Cross-scale Synthesis of Organic High-k Semiconductors with Ultralow Energy Disorder from Spiro-gridized Nanopolymers**

Dongqing Lin^1^, Wenhua Zhang^2^, Hang Yin^3^, Haixia Hu^3^, Yang Li^1^, He Zhang^1^, Le Wang^1^, Xinmiao Xie^1,4^, Hongkai Hu^1^, Yongxia Yan^1^, Haifeng Ling^1^, Jin’an Liu^1^, Yue Qian^1^, Lei Tang^1^, Yongxia Wang^1^, Chaoyang Dong^1^, Linghai Xie^1,6*^, Hao Zhang^5^, Shasha Wang^1^, Ying Wei^1*^, Xuefeng Guo^4^, Dan Lu^5^, Wei Huang^1,6*^

^1^Centre for Molecular Systems and Organic Devices (CMSOD), State Key Laboratory of Organic Electronics and Information Displays & Institute of Advanced Materials (IAM), Nanjing University of Posts & Telecommunications, 9 Wenyuan Road, Nanjing 210023, China

^2^National Synchrotron Radiation Laboratory, Anhui Provincial Engineering Laboratory of Advanced Functional Polymer Film, CAS Key Laboratory of Soft Matter Chemistry, University of Science and Technology of China, Hefei 230026, China

^3^School of Physics, State Key Laboratory of Crystal Materials, Shandong University, Jinan, Shandong 250100, P. R. China

^4^Beijing National Laboratory for Molecular Sciences, National Biomedical Imaging Center, College of Chemistry and Molecular Engineering, Peking University, Beijing 100871, P. R. China.

^5^State Key Laboratory of Supramolecular Structure and Materials, College of Chemistry, Jilin University, 2699 Qianjin Avenue, Changchun, 130012, China

^6^Frontiers Science Center for Flexible Electronics (FSCFE), MIIT Key Laboratory of Flexible Electronics (KLoFE), Northwestern Polytechnical University, Xi'an 710072, China

**Contents**

**1. Materials and Methods.**

**2. Methods**

**3. Three-dimensional models of the rhombus-type grid unit (RG), spirodigrid (SDG) and nanopolyspirogrid (NPSG)**

**4. The supplementary points of spiro-polygridization**

**5. Average degree of polymerization (*DP*) of NPSG nanochains**

**6. Characterizations of NPSG chains**

**7. Single-chain models and properties of NPSG**

**8. Calculating the persistence length via molecular dynamic simulations**

**9. Dielectric properties**

**10. Optoelectronic properties** **of NPSG**

**11. Amorphous properties of NPSG film**

**12. Electric properties of NPSG film**

**13. NMR spectra**

**14. References:**

**1. Materials and Methods.**

**1.1 Materials**

All the solvents and reagents were purchased from commercial suppliers and used without further purification, unless noted otherwise. Dichloromethane (DCM) and 1,2-dichloroethane (DCE) were dried by anhydrous sodium under room temperature. Tetrahydrofuran and toluene were dried over sodium benzophenone ketyl anion radical and distilled under a dry nitrogen atmosphere immediately prior to use. BF_3_∙OEt_2_ (47 wt%), [potassium carbonate](javascript:showMsgDetail('ProductSynonyms.aspx?CBNumber=CB4853879&postData3=CN&SYMBOL_Type=A');), magnesium sulphate, chloroform, and toluene were purchased from Sinopharm Chemical Reagent Co, Ltd. without further purification. Palladiumacetate (Pd(OAc)_2_), tetra(triphenylphosphine)palladium (Pd(PPh_3_)_4_), 1,1'-bis(diphenylphosphino)ferrocene (dppf), 2-bromo-9-fluorenone, 2,7-dibromo-9-fluorenone (2,7-DBrFO), 4-bromophbenol and 1-bromooctane were obtained from Aldrich Chemical Co. The synthetic procedures of 2-bromo-3,3'-bithiophene (as the primary reagent of the synthon SXs) and 2-bromospiro[fluorene-9,8'-indeno[2,1-*b*]thiophene] (Thspiro, as the moiety of the end-capping reagent ML) are the same as the literature [*1*]. The synthetic procedure of reagent 9-(4-(octyloxy)phenyl)-2-(4,4,5,5-tetramethyl-1,3,2-dioxaborolan-2-yl)-9*H*-fluoren-9-ol (FOHBpin) refers to the literature [*2*].

**Synthetic Procedure**

**Figure S1. Synthetic routes of NPSG.** As the synthesis of NPSG is dependent on the reaction time and the concentration that are carefully discussed in the manuscript, we do not provide the yields of NPSG, HBPG and CLP.

**9-([3,3'-Bithiophen]-2-yl)-2,7-dibromo-9*H*-fluoren-9-ol (1)**

The synthetic procedure of **1** is referred to our previous work [1]. The reagents include 2,7-dibromo-9-fluorenone (**2,7-DBrFO**, 3.38 g, 10 mmol, 1 equiv), magnesium (0.312 mg, 13 mmol, 1.3 equiv), a drop of iodine, 2-bromo-3,3'-bithiophene (2.94 g, 12 mmol, 1.2 equiv). The THF solvent was used in the Grignard reagent (8 ml) and in the reaction solution (40 ml). After the purification process via column chromatography [silica gel, petroleum ether: DCM = 2:1], the products **1** were obtained in 70% yield. (White powders, 3.51 g, 7 mmol). ^1^H NMR (400 MHz, CDCl_3_) δ 7.46 – 7.45 (d, *J* = 2.0 Hz, 1H), 7.40 – 7.37 (dd, *J* = 8.0, 1.6 Hz, 2H), 7.29 – 7.28 (d, *J* = 5.2 Hz, 1H), 7.22 – 7.20 (d, *J* = 8.0 Hz, 2H), 6.85 – 6.83 (d, *J* = 5.2 Hz, 1H), 6.82 – 6.80 (dd, *J* = 5.0, 3.0 Hz, 1H), 6.14 – 6.12 (d, *J* = 4.8Hz, 1H), 6.11 – 6.09 (d, *J* = 3.2Hz, 1H), 2.70 (s, 1H). ^13^C NMR (100 MHz, CDCl_3_) δ 150.4, 141.0, 137.5, 135.5, 133.1, 132.5, 131.1, 128.1, 128.1, 123.8, 123.0, 122.8, 122.2, 121.5, 81.6. HRMS (ion: MALDI, FT-ICR-MS): m/z calcd for [M^+^] C_21_H_11_OBr_2_S_2_: 500.8624; found: 500.8621.

**2',7'-Dibromospiro[cyclopenta[1,2-*b*:4,3-*b*']dithiophene-7,9'-fluorene] (2):**

Add **1** (0.504 g, 1 mmol, 1 equiv) in DCM solvent (50 ml), Then drop BF_3_∙OEt_2_ (47 wt%, 0.65 ml, 2.5 mmol, 2.5 equiv) that were dissolved in 200 ml DCM solvent. The reaction mixture was stirred at 20^o^C for 3 h and then was quenched via excessive KOH aqueous. The mixture was extracted by DCM solvent and we removed DCM solvent through evaporation under reduced pressure. Purification by recrystallization from THF and petroleum ether afforded **2** (white powder, 0.437 g, 0.9 mmol) in 90% yield. ^1^H NMR (400 MHz, CDCl_3_) δ 7.63 – 7.61 (d, *J* = 8.0 Hz, 2H), 7.52 - 7.50 (dd, *J* = 8.0, 2.0 Hz, 2H), 7.37 – 7.36 (d, *J* = 4.8 Hz, 2H), 7.20 – 7.19 (d, *J* = 4.8 Hz, 2H), 7.01 – 7.00 (d, *J* = 2.0 Hz, 2H). ^13^C NMR (100 MHz, CDCl_3_) δ 148.8, 148.6, 144.0, 138.9, 131.8, 129.5, 127.1, 122.1, 121.7, 118.3, 62.0. HRMS (ion: MALDI, FT-ICR-MS): m/z calcd for [M^+^] C_21_H_11_Br_2_S_2_: 484.8663; found: 484.8662.

**2,2'-(Spiro[cyclopenta[1,2-*b*:4,3-*b'*]dithiophene-7,9'-fluorene]-2',7'-diyl)bis(9-(4-(octyloxy)phenyl)-9*H*-fluoren-9-ol) (STF-DOH)**

In a three-necked flask, **2** (0.486 g, 1 mmol, 1 equiv), 9-(4-(octyloxy)phenyl)-2-(4,4,5,5-tetramethyl-1,3,2-dioxaborolan-2-yl)-9*H*-fluoren-9-ol (**FOHBpin** synthesized in our previous work [2], 1.12 g, 2.2 mmol, 2.2 equiv), Pd(PPh_3_)_4_ (116 mg, 0.1 mmol, 0.1 equiv), toluene/THF mixed solvents (5 mL) and K_2_CO_3_/KF aqueous solution (2 M, 2 mL, 4 equiv) were added under the nitrogen atmosphere. The mixture was heated up to 90 °C and stirred for 24 hours. The mixture was extracted via DCM solvent and the organic layer was dried over MgSO_4_. Then the solvent were removed by evaporation under reduced pressure. Purification by silica gel column chromatography (petroleum ether: DCM = 1:5) afforded the **STF-DOH** synthon in 70% yield (yellow powder, 0.767 g, 0.7 mmol). ^1^H NMR (400 MHz, CDCl_3_) δ 7.81 – 7.79 (d, *J* = 8.0 Hz, 2H), 7.62 – 7.58 (m, 6H), 7.49 – 7.46 (m, 4H), 7.36 – 7.33 (m, 2H), 7.32 – 7.30 (m, 6H), 7.29 – 7.27 (m, 2H), 7.25 – 7.19 (m, 4H), 7.14 – 7.12 (m, 2H), 6.78 – 6.76 (d, *J* = 8.8 Hz, 4H), 3.90 – 3.87 (t, *J* = 7.4 Hz, 4H), 2.57 (s, 2H), 1.78 – 1.71 (m, 4H), 1.44 – 1.40 (m, 4H), 1.34 – 1.30 (m, 16H), 0.93 – 0.90 (m, 6H). ^13^C NMR (100 MHz, CDCl_3_) δ 158.4, 151.2, 150.9, 150.2, 148.2, 143.9, 141.2, 141.0, 139.9, 138.9, 138.8, 134.9, 129.0, 128.4, 128.2, 127.7, 126.6, 124.7, 123.3, 122.2, 120.6, 120.3, 120.1, 118.4, 114.2, 83.4, 67.9, 62.7, 31.9, 29.8, 29.4, 29.3, 29.3, 26.1, 22.7, 14.2. FT-IR spectra: 3438, 3068, 3040, 2927, 2856, 1607, 1579, 1506, 1466, 1450, 1339, 1300, 1246, 1166, 1029, 919, 891, 823, 803, 779, 752, 736, 707, 691 cm^-1^. MALDI-TOF-MS: m/z calcd for [M+] C_112_H_86_N_2_: 1096.46; Found: 1094.43.

**9-(4-(Octyloxy)phenyl)-2-(spiro[fluorene-9,8'-indeno[2,1-b]thiophen]-2-yl)-9*H*-fluoren-9-ol (ML, as the end-capping reagent of the polygridization)**

In a three-necked flask, 2-bromospiro[fluorene-9,8'-indeno[2,1-*b*]thiophene] (Thspiro synthesized in our previous work [2], 0.403 g, 1 mmol, 1equiv), **FOHBpin** (0.56 g, 1.1 mmol, 1.1 equiv), Pd(PPh_3_)_4_ (116 mg, 0.05 mmol, 0.05 equiv), toluene/THF mixed solvents (5 mL) and K_2_CO_3_/KF aqueous solution (2 M, 1 mL, 2 equiv) were added under the nitrogen atmosphere. The mixture was heated up to 90 °C and stirred for 24 hours. The mixture was extracted via DCM solvent and the organic layer was dried over MgSO_4_. Then the solvent were removed by evaporation under reduced pressure. Purification by silica gel column chromatography (petroleum ether: DCM = 1:1) afforded the **ML** in 78% yield (white powder, 0.55 g, 0.78 mmol). ^1^H NMR (400 MHz, CDCl_3_) δ 7.86 – 7.82 (t, *J* = 7.8 Hz, 2H), 7.63 – 7.58 (m, 4H), 7.46 – 7.44 (m, 2H), 7.42 – 7.41 (d, *J* = 5.2 Hz, 1H), 7.39 – 7.35 (m, 3H), 7.35 – 7.33 (d, *J* = 7.6 Hz, 2H), 7.32 – 7.27 (m, 3H), 7.24 – 7.20 (t, *J* = 7.2 Hz, 1H), 7.15 – 7.11 (t, *J* = 7.6 Hz, 1H), 7.03 (s, 1H), 7.00 – 6.93 (m, 1H, with two triplet peaks *J* = 7.6 Hz), 6.81 – 6.79 (d, *J* = 8.4 Hz, 1H), 6.77 – 6.74 (d, *J* = 8.8 Hz, 2H), 6.67 – 6.62 (m, 1H, with two double peaks *J* = 7.6 Hz), 3.89 – 3.86 (t, *J* = 6.8 Hz, 2H), 2.43 (s, 1H), 1.76 – 1.69 (m, 2H), 1.43 – 1.38 (m, 2H), 1.28 – 1.26 (m, 8H), 0.90 – 0.86 (m, 2H). ^13^C NMR (100 MHz, CDCl_3_) δ 158.4, 153.0, 152.9, 151.2, 150.9, 149.4, 148.3, 148.2, 148.2, 147.9, 147.8, 141.3, 141.0, 140.9, 140.8, 140.8, 139.0, 139.0, 138.7, 134.9, 130.3, 130.3, 129.0, 128.4, 128.2, 128.2, 128.0, 127.6, 127.6, 127.4, 126.6, 125.7, 125.7, 124.7, 123.8, 123.7, 123.7, 123.3, 123.3, 122.4, 122.4, 120.5, 120.3, 120.2, 120.1, 119.5, 119.4, 118.7, 118.7, 114.2, 83.4, 67.9, 64.0, 31.9, 29.4, 29.3, 29.3, 26.1, 22.7, 14.2. HRMS (ion: MALDI, FT-ICR-MS): m/z calcd for [M^+^] C_50_H_42_O_2_S: 706.2900; found: 706.2900.

**Nanopolyspirogrid (NPSG)** via polygridizations

Add **STF-DOH** (110 mg, 0.1 mmol, 1 equiv) in DCE solvent (50 ml), then stir this solution in 5 min, which is followed by the addition of BF_3_∙OEt_2_ catalyst (47 wt%, 2 ml, 7.6 mmol, ~80 equiv). After specific time (5 min~8 h, especially for 4 h), we added end-capping reagent ML to terminate this polygridization and added excessive KOH aqueous solution to quench this polygridization. The mixture was extracted via DCM solvent and the organic layer was dried over MgSO_4_. Then the solvent were removed by evaporation under reduced pressure. The film-like products was further purified by Soxhlet extraction via THF and acetone mixing solvents. If the insoluble cross-linking polymers **CLP** were generated (in much STF-DOH concentration up to 12~16 mM), we would filtered the **CLP** solid and then performed the extraction. After the Soxhlet extraction, we obtained **NPSGs** in 60% yields (yellow film-like state, 66 mg). Probably due to the absence in stereoselectivity, the ^1^H NMR spectra (Figure S84) exhibits drastically broad peaks at 8.10~6.60 ppm and at 4.20~3.50 ppm, which cannot be analyzed. Similarly, the carbon resonance signals are weak and become broad peaks (Figure S85), apart from some of sp^3^-carbon atoms with clear signals at 67.9, 62.6 (spiro-carbon, but rather weak), 31.8, 29.7, 29.4, 26.9, 26.1, 22.7, 14.1. Fortunately, the FT-IR spectra can qualitatively demonstrate this backbone structure: 3063, 3035, 2921, 2853, 1610, 1577, 1506, 1466, 1448, 1295, 1248, 1177, 1025, 820, 783, 743, 724 cm^-1^.

For soluble CLP, we characterized their FT-IR spectra: 3063, 3035, 2927, 2853, 1607, 1579, 1509, 1464, 1450, 1419, 1380, 1328, 1295, 1246, 1180, 1029, 820, 783, 745, 722 cm^-1^.

**Spirodigrid (SDG)**

Add **STF-DOH** (110 mg, 0.1 mmol, 1 equiv) and **ML** (141 mg, 0.2 mmol, 2 equiv) in DCE solvent (50 ml), then stir this solution in 5 min, which is followed by the addition of BF_3_∙OEt_2_ catalyst (47 wt%, 2 ml, 7.6 mmol, ~80 equiv). After 30 min, the reaction was quenched by excessive KOH aqueous solution. Then, the mixture was extracted by DCM solvent and we removed DCM solvent via evaporation under reduced pressure. Purification by silica gel column chromatography (petroleum ether: DCM = 2.5:1) afforded the **SDG** in 11% yield (dilute yellow powder, 27 mg, 0.011 mmol). Considering the massive stereoisomers, we further tentatively isolated some of stereoisomers via silica gel column chromatography (petroleum ether: DCM: acetone = 30:6:1) and luckily obtained the specific configurations with relatively well-defined NMR signals, though the stereoisomers were too difficult to identify. ^1^H NMR (400 MHz, CDCl_3_) δ 7.74 – 7.72 (d, *J* = 8.0 Hz, 2H), 7.70 – 7.68 (d, *J* = 7.6 Hz, 2H), 7.67 – 7.64 (d, *J* = 8.0 Hz, 4H), 7.63 – 7.54 (m, 12H), 7.54 – 7.46 (m, 10H), 7.42 – 7.40 (d, *J* = 8.8 Hz, 4H), 7.36 – 7.35 (d, *J* = 2.8 Hz, 2H), 7.32 – 7.30 (d, *J* = 6.8 Hz, 4H), 7.29 – 7.28 (m, 4H), 7.16 – 7.14 (d, *J* = 7.2 Hz, 2H), 7.13 (s, 2H), 7.10 – 7.07 (d, *J* = 8.4, 2.8 Hz, 4H), 7.06 – 7.04 (d, *J* = 8.0 Hz, 2H), 6.97 (s, 2H), 6.93 – 6.89 (m, 8H), 6.83 – 6.81 (d, *J* = 7.6 Hz, 4H), 6.79 – 6.77 (d, *J* = 9.2 Hz, 4H), 6.59 – 6.57 (d, *J* = 8.8 Hz, 2H), 3.91 – 3.88 (t, *J* = 5.8 Hz, 8H), 2.31 – 2.21 (m, 4H), 2.06 – 1.97 (m, 4H), 1.79 – 1.70 (m, 8H), 1.30 – 1.29 (m, 32H), 0.86 – 0.83 (m, 12H). ^13^C NMR (100 MHz, CDCl_3_) δ 158.5, 158.3, 156.1, 152.2, 152.0, 151.5, 151.3, 151.3, 148.0, 147.7, 147.7, 147.6, 147.5, 147.1, 147.0, 147.0, 146.9, 143.5, 141.0, 140.5, 140.2, 140.0, 139.9, 139.8, 139.8, 139.7, 139.3, 139.1, 138.9, 138.8, 138.7, 138.5, 138.4, 136.8, 136.6, 135.1, 124.5, 124.0, 123.7, 123.6, 123.5, 123.4, 121.9, 121.8, 120.2, 119.1, 114.3, 114.2, 114.1, 68.1, 67.9, 64.2, 62.5, 62.3, 32.0, 31.5, 31.5, 30.2, 30.1, 29.7, 29.4, 26.2, 26.1, 22.7, 14.2. MALDI-TOF-MS: m/z calcd for [M+] C_175_H_144_O_4_S_4_: 2439.01; Found: 2439.00.

**2. Methods**

**Nuclear Magnetic Resonance (NMR)**

^1^H and ^13^C NMR data were received from a Bruker 400 MHz NMR Fourier transform spectrometer (400 MHz and 100 MHz, respectively) at 20 ^o^C. Chemical shifts are shown as δ in units of parts per million (ppm) relative to internal standard [^1^H NMR: tetramethylsilane (TMS) = 0.00 ppm] or relative residual peaks (^1^H NMR: 7.26 for CDCl_3_; ^13^C NMR: 77.0 triplet for CDCl_3_). Multiplicities of every signal peak in briefly were shown as: s (singlet); d (doublet); t (triplet); q (quartet); dd (doublet of doublets); dt (doublet of triplets); m (multiplet). Coupling constants are expressed as a *J* value in Hz.

**Fourier Transform Infrared spectrometer (FT-IR)**

The spirodithiophenefluorene-containing difluorenyl synthon (STF-DOH), spirodigrid (SDG, *DP* = 2 of NPSG), nanopolyspirogrid (NPSG) and cross-linking polymer (CLP) samples were addressed as KBr pellets. Their infrared absorption spectrometer were characterized by Fourier Transform Infrared Spectrophotometer (SHIMADZU, IRPrestige-21).

The simulation of the vibration modes are the same as the method in the literature [*3*], especially for the RB3LYP method, the basic set of 6-31G(D) and the scaling factor 0.9614 [*4*].

**Molar Mass for substrates and SDG products**

The Matrix-Assisted Laser Desorption/Ionization Time of Flight Mass Spectrometry (MALDI-ToF-MS, Bruker, reflective mode) and High Resolution Mass Spectroscopy HRMS (Thermo Fisher Scientific LTQ FTICR-MS) were used as the determination of molecular weight.

**Gel permeation chromatography (GPC).** The GPC characteristics were conducted on a HP1100 HPLC system possessing 7911GP-502 and GPC columns using polystyrenes as the standard and tetrahydrofuran (THF) as the eluent at a flow rate of 1.0 mL/min at 25 ^o^C. The concentrations of NPSG solutions were about 0.8 mg/ml.

**Dynamic Light Scattering (DLS) and Static Light Scattering (SLS).** The DLS characterizations of NPSG solution (CHCl_3_ as a solvent) were determined by a Brookhaven instrument (ZetaPALS) to obtain *R*_h_. The operating wavelength of light source is 632.8 nm. The SLS measurements were performed to calculate *R*_g_, via ALV/CGS-3 light-scattering spectrometer that is equipped with an ALV/LSE-7004 multiple-τ digital correlator. All of the operating wavelength are 632.8 nm for light source.

**Visualization via Atomic Force Microscopy (AFM).**

The NPSG solution (in CHCl_3_ solvent, the concentration of 2 ug/ml) was spin-coated on the the mica sheet. The AFM (Bruker, dimension-icon) characterizations were used to tentatively visualize the single-chain NPSG morphology, under the scanasyst mode (in peak-force tapping type) and the supersharp tips (SAA-HPI-SS). In this case, we can measure the length and the height of single-chains, but we cannot accurately measure the width because of the unavoidably tip-broadening effect [*5*].

**Small-angel X-ray Scattering (SAXS).** The scattering datum were provided via the synchrotron radiation SAXS from Shanghai Synchrotron Radiation Facility. The distance from the sample cell (with mica windows in the path length of 1.5 mm) to the detector is 1 m. The wavelength of X-ray is 1.2 Å. The collected scattering vectors *q* ranges from 0.01 to 0.4 nm. The substrate of the solvent (toluene) from the solution scattering was performed before the analysis. The data were collected with the exposure time of 2 s, the acquired period of 2.01 s, and the images of 10. The measured NPSG solutions were 0.5~2 mg/ml in toluene solvents.

**Molecular dynamic simulations.** The molecular models of NPSG-based spirotrigrids were constructed and calculated via the Forcite plus module in the software Material Studio. The geometry optimization were performed based on the SMART algorithm (as the cascade Steepest Descent-ABNR-Quasi-Newton algorithm). The simulations of conformational motions of spirotrigrids were based on the NVT ensemble (in vacuum), the pcff forcefield, the time-step of 0.2 fs and the total time of 100 ps.

**Ultraviolet-Visual absorption (UV-Vis) and Photoluminescence spectra (PL).** The solution was prepared under the concentration of 10^-2^ mg/ml in CHCl_3_ or DCE solvent. The film was spin-coated from the solution (DCE solvent, the concentration of 8 mg/ml) under the 800 rad/s. The UV spectra (LAMBDA 35) were used to characterize the photophysical properties of their ground states. The PL spectra (RF-6000 Plus) were obtained to study the excitonic behaviors of their excited states.

**The transient decay spectra of NPSG solution**. The transition decay were characterized via Edinburgh FLSP920 fluorescence spectrophotometer via the laser with the wavelength of 335 nm.

**Measurement of space-charged-limited current for carrier mobility (*μ*).** The hole-only device with the structure of ITO/PEDOT:PSS/NPSG/Au was fabricated according to the literature [*6*]. The poly(3,4-ethylenedioxythiophene)-doped poly(styrene sulfonic acid) (PEDOT:PSS) layer and NPSG layer (or SDG oligomers, both in chlorobenzene solvent) were prepared via spin-coating at the spin rate 7000 RPM and annealing under 140 °C (10 min). For the space-charged-limited current measurement, injecting the charge carriers into the thin-film active layer was performed under the DC voltage, through a source measure unit (SMU) Keithley (Model 2612B) that also record the currents under different voltage conditions. The energy disorder (*σ*) was calculated via the equation *μ* (*T*) = *μ*_∞_ exp [-(2*σ*/3k_B_*T*)^2^], where *μ*_∞_, k_B_ and *T* are defined as the carrier mobility at ultrahigh temperature, the Boltzmann constant and the temperature, respectively. The activation energy (*E*_a_) of carrier transport was calculated via the equation *μ* (*T*) = *μ*_∞_ exp (-*E*_a_/k_B_*T*).

**The molecular orbital distributions of the spiro-trimer, SDG, spirotrigrid and spirotetragrid (*DP* = 4 of NPSG).** The molecular orbital distributions were obtained from the geometry optimization in quantum chemistry calculation. The molecules in our system were optimized by DFT calculation at the B3LYP/6-31G (d) level. All the quantum chemistry calculations were carried out using the Gaussian 09 program [*7*].

**Single-molecular electronic device.** A new dash-line lithographic (DLL) method, referred to the literature [*8*], was used to fabricate the single-molecular electronic device. For the linkage of a polymeric single-chain via ester linkage, NPSG were dissolved in dichloromethane with the concentration ~10^-4^ M, followed by adding graphene devices and carbodiimide dehydrating/activating agent. After 2 days, the NPSG device was taken out from solution. Such device was washed through copious acetone and ultrapure water solvent, and then was dried under N_2_ atmosphere. We used an Agilent 4155C semiconductor characterization system and a Karl Suss (PM5) manual probe station to measure the current (I)-voltage (V) curve under the ambient atmosphere.

**The calculation of dielectric constant (*k*) of NPSG.** The diode device structure Cu/SiO_2_/NPSG/Si (n^+^) was fabricated to measure the total capacitance *C*_i_, which is transformed to *k* via the equation *ε*_0_/C_i_ = *d*_s_/*k_s_* - *d*/*k*, where *d*_s_ and *k_s_*, *ε*_0_ are defined as the layer thickness, the dielectric constant of SiO_2_ (*k_s_ =* 4.0) [*9*] and the permittivity of vacuum (8.85 × 10^−12^ F/m), respectively. The NPSG layer (from the DCE solution with the NPSG concentration of 5 mg/ml) was spin-coated on the SiO_2_ layer, which is followed by annealing under the conditions of vacuum environment, 80 ℃ and 30 min. The procedure and conditions of spin-coating other polymer/oligomer films were the same as those of NPSG. *C*_i_ was measured via an impedance analyzer (IM3533), through a frequency sweep of 500~100000 Hz and a bias of 0.1 V. The thicknesses of the NPSG film was measured by an ellipsometry (J.A. Woollam RC2). The dielectric loss (tan *δ*) of NPSG-based diode device (bilayer of NPSG and SiO_2_) was afforded via impedance analyzer at 10^3^~10^5^ Hz. The optical dielectric constant (*k*_o_) of NPSG, in the high frequency range of 3 × 10^14^ ~ 8 × 10^14^ Hz, was calculated via the equation *k*_o_ = *n*^2^ – *K*^2^, where *n* and *K* are defined as the refractive index and the extinction coefficient, respectively. Both were afforded via the ellipsometry characterization.

**OFET device fabrication.** We used a heavily doped n-type Si wafer (as the control gate) containing 50 nm-thick SiO_2_ layer, which serve as the control dielectric layer. The surface of Si wafer is carefully washed by acetone, ethanol and deionized water in 20 minutes, via ultrasonic cleaning, which is followed by blowing with nitrogen atmosphere. Then, these wafers were dry over in the vacuum atmosphere (under the temperature of 120 ℃ and 30 minutes). The polystyrene or the mixed polystyrene and NPSG samples were were dissolved in 1, 2-dichloroethane and then spin coated on SiO_2_ as polymer dielectric layer. The semiconductor layer of 50 nm-thick pentacene was deposited onto PS layer or mixed PS and NPSG layer, under the thermal vacuum evaporation method at 5×10^4^ Pa. The Cu film with the thickness of 100 nm, serving as the source and drain electrodes, was thermally evaporated through shadow mask. The channel length (*L*) and width (*W*) were 150 and 1500 μm, respectively. All of the devices were synchronously fabricated at the same conditions and characterized in a shielding box in ambient air (*RH* = 2%), using a Keithley 2636B semiconductor parameter analyzer.

**3. Three-dimensional models of the rhombus-type grid unit (RG), spirodigrid (SDG) and nanopolyspirogrid (NPSG)**


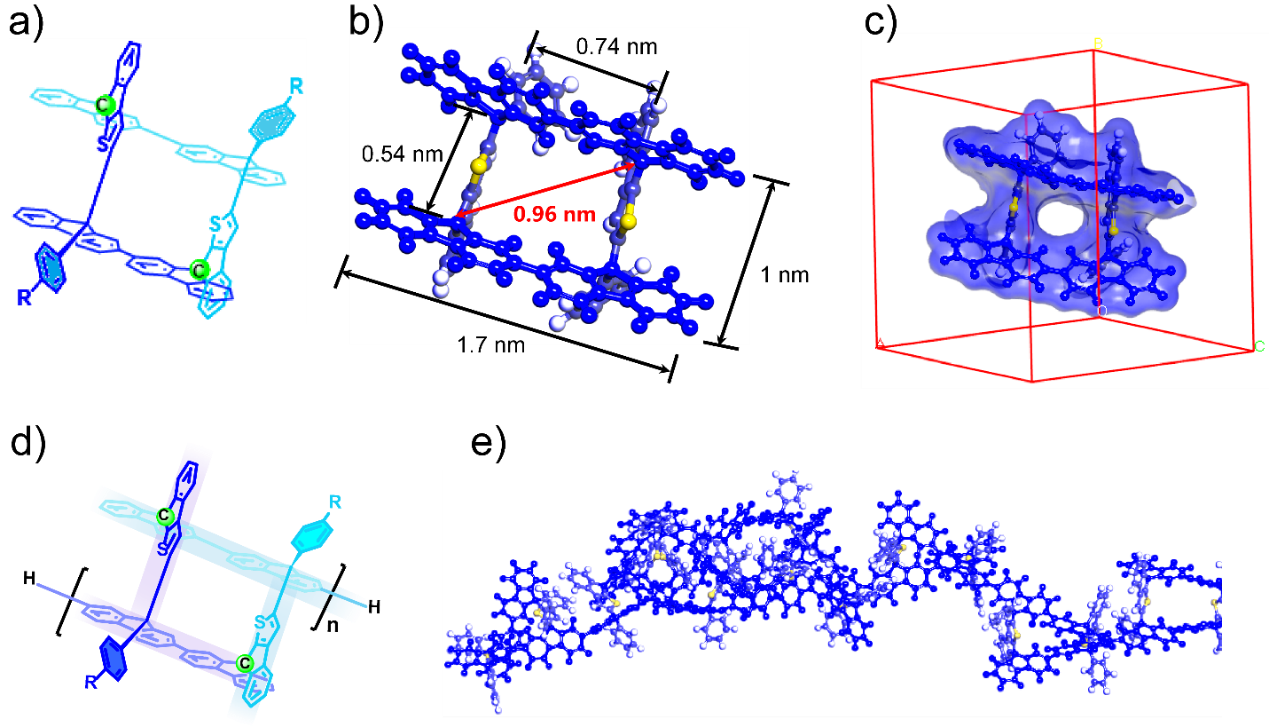


**Figure S2. The nanoarchitectures of rhombus-type grids (RGs) and single-bond-linked polygrids (SBPGs).** (a) The general molecular structures of RG in this work. The R groups represent the alkyl chains or hydrogen atoms. The green dots are marked as the spiro-carbon atoms. (b) The three-dimensional model of RG with various sizes. The blue parts are fluorene groups. (c) The detection of free volume for RG. The dilute blue region is defined as the RG’s volume during which the van de Waals factor is set as 1.0 and the dynamic diameters of the detected tip is set as 0.34 nm. The dynamic diameters of waters are 0.27~0.32 nm. The white region is defined as the free volume that can be packed with guest molecules. Due to the existence of the free volume, these RG units potentially capture or transport waters. (d) The molecular structure of SBPGs. (e) The nanoarchitectural model of SBPGs.


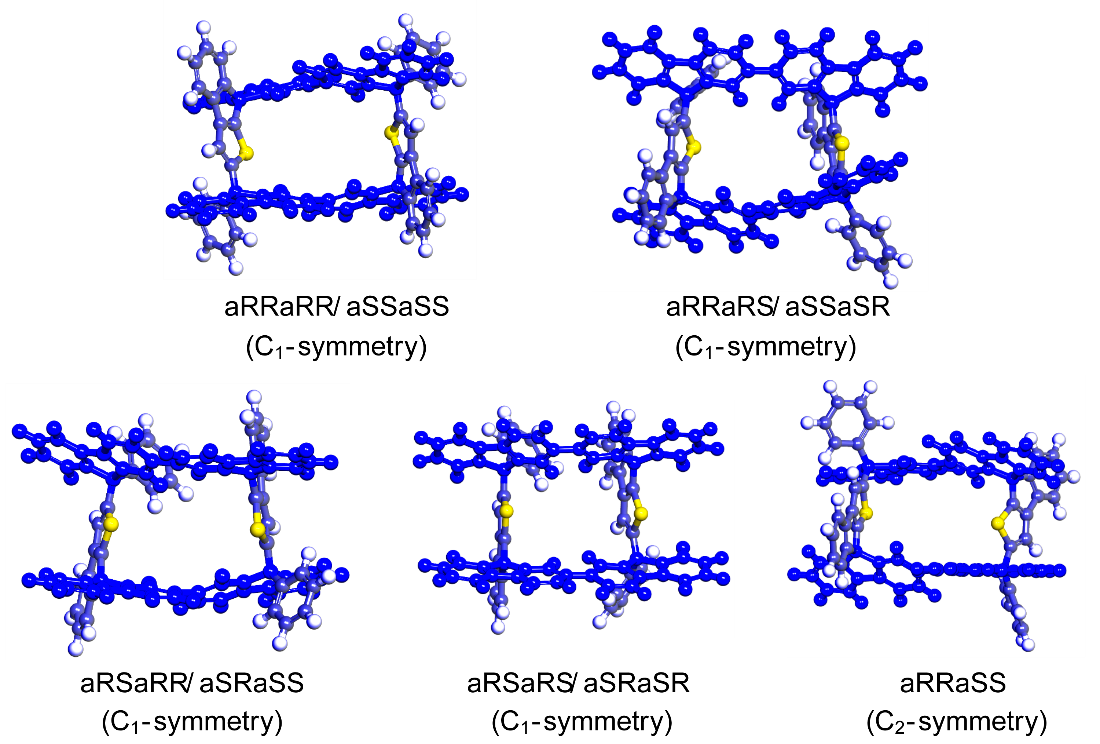


**Figure S3. The diastereoisomers of RG.** The alkyl chains are omitted.

As the repeat unit, a rhombus-type gridarene (RG) comprises two diarylfluorenes and two spiroarenes to form the chemical composition of C_84_H_48_S_2_ with the molecular weight of 1120 m/z (without alkyl chains, Figure S2). Its backbone length measured at the bifluorenyl and spiro-planar edges are 1.7 nm and 1 nm, respectively. Built up from these edges, the internal pore (enclosed by 22 atoms) displays the size of 0.4 nm^2^ where two spiro-carbon atoms are diagonally distributed in the distance of 0.96 nm, which likely transport water-like molecules within the dynamic diameters of 0.34 nm. Considering two chiral diarylfluorenes and two axis-chiral spiroarenes in centrosymmetric distribution, RG has 9 stereoisomers that are divided into four pairs of C_1_-symmetric *rac*-isomers and a C_2_-symmetric *meso*-configurations (Figure S3).


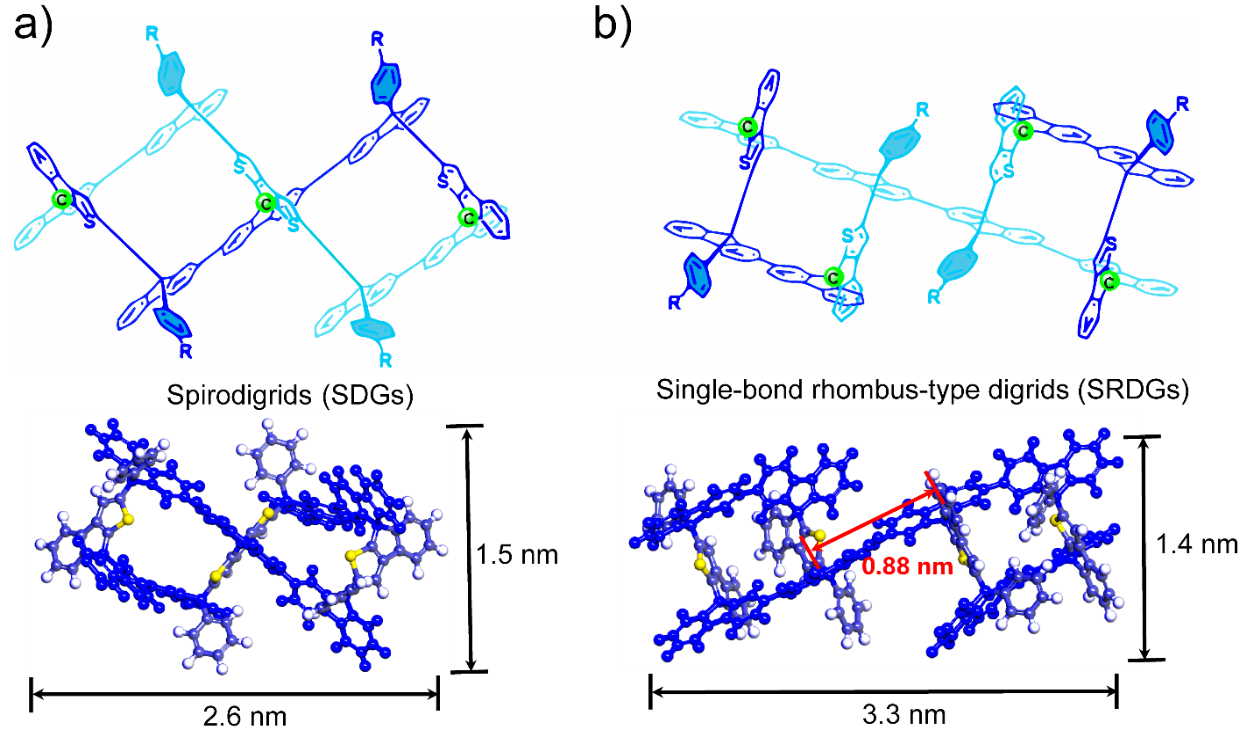


**Figure S4. The nanoarchitectures of spirodigrids (SDGs, a) and single-bond-linked rhombus-type digrids (SRDGs, b)**. The corresponding length of edges and the distance between to grid pores are depicted as well in atomistic models.


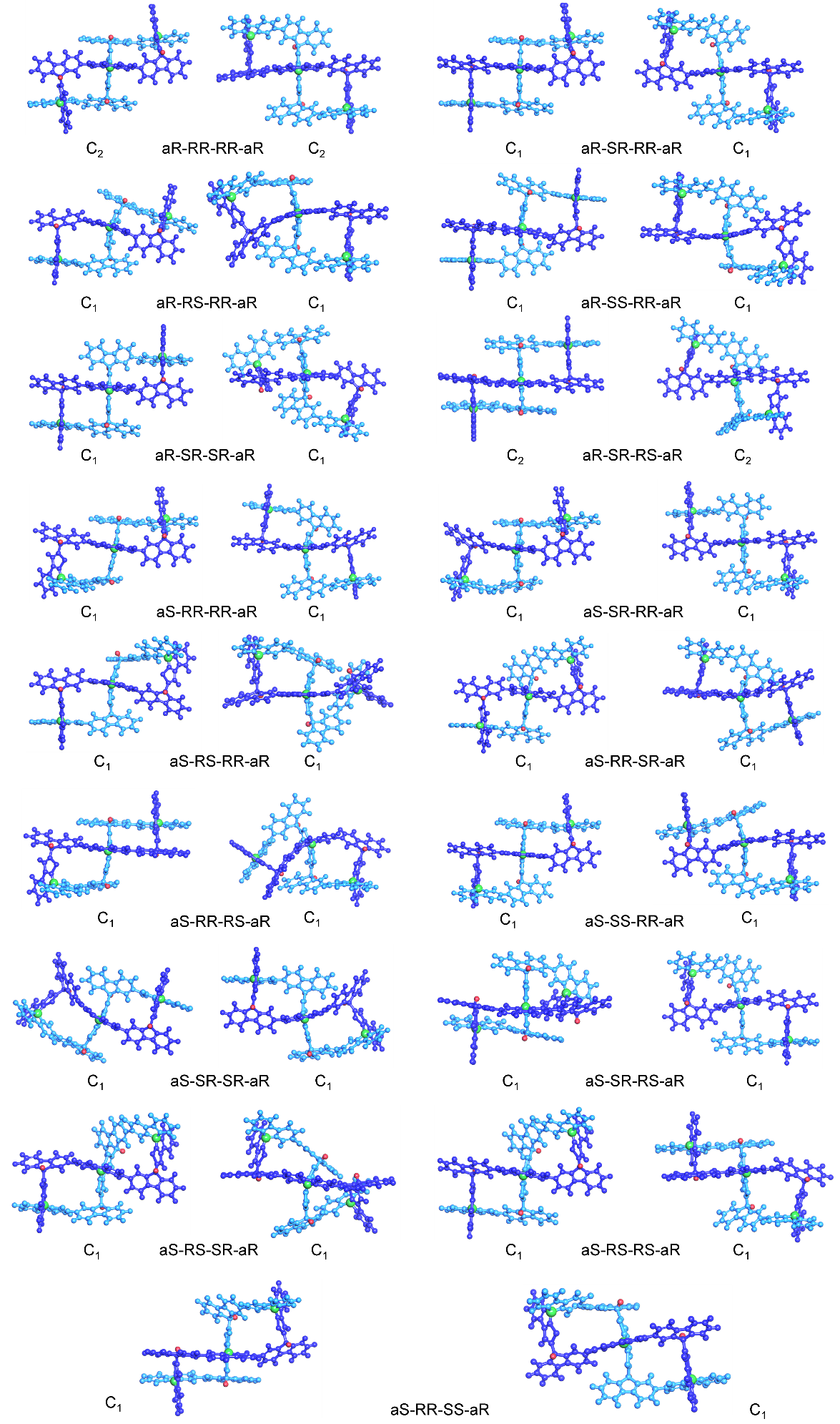


**Figure S5. The diastereoisomers of SDG.** We also considered their symmetry and the axis-chirality of spirodithiophenefluorene (SDTF)-core. The benzyl groups attached on the 9-positions of fluorenes are omitted.

We extended DG into a spirodigrid (SDG) with the chemical composition of C_143_H_80_S_4_ and the molecular weight of 1924 m/z (Figure S4). Such nanoarchitectural length is measured as 2.6 nm where two grid pores are merely separated by a spiro-carbon atom, as more structurally compact than the single-bond-linked rhombus-type digrid (DRG) with the distance of 0.88 nm between two pores (Figure S4b). Composed of four chiral diarylfluorenes and three axis-chiral spiroarenes including the central SDTF moiety, SDG possesses sixty-eight stereoisomers with the maximum C_2_-symmetry (Figure S5).

We installed the nanoarchitectural models of NPSGs based on the structural features of RG unit. Considering the diarylfluorenyl chirality on RG vertices, we roughly categorized the tacticity of NPSGs into *SS*-isotacticity, *SR*-isotacticity, *SS*-*SR*-syndiotacticity and *SS*-*RR*-syndiotacticity. For each tacticity, we further considered the SDTF-based axis-chirality that determines the interlaced or uninterlaced double-strand topology. However, the uninterlaced SS-RR-syndiotactic NPSG nanochains cannot be constructed because of the larger internal strain energy. Therefore, we constructed NPSG nanochains in interlaced *SS*-isotacticity, uninterlaced *SS*-isotacticity, interlaced *SR*-isotacticity, uninterlaced *SR*-isotacticity, interlaced *SS*-*SR*-syndiotacticity, uninterlaced *SS*-*SR*-syndiotacticity and interlaced *SS*-*RR*-syndiotacticity. For single-bond-linked SBPG nanochains, we constructed them in corresponding tacticities, including *SS*-isotacticity, *SR*-isotacticity, *SS*-*SR*-syndiotacticity and *SS*-*RR*-syndiotacticity.


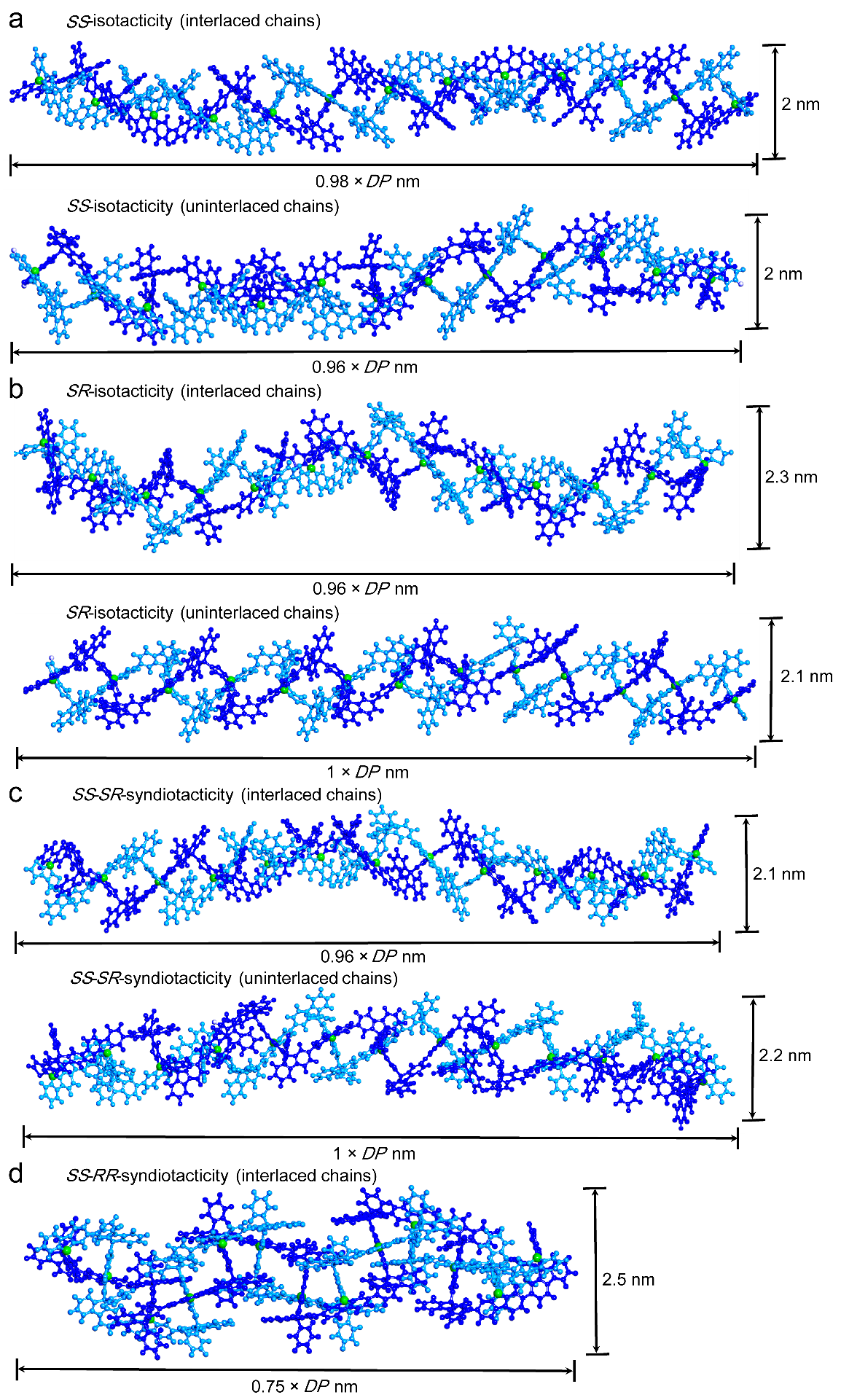


**Figure S6. The NPSG chains with various tacticity and interlaced or uninterlaced types.** (a) *SS*-isotacticity. (b) *SR*-isotacticity. (c) *SS-SR*-syndiotacticity. (d) *SS-RR*-syndiotacticity. The chain sizes are also provided.


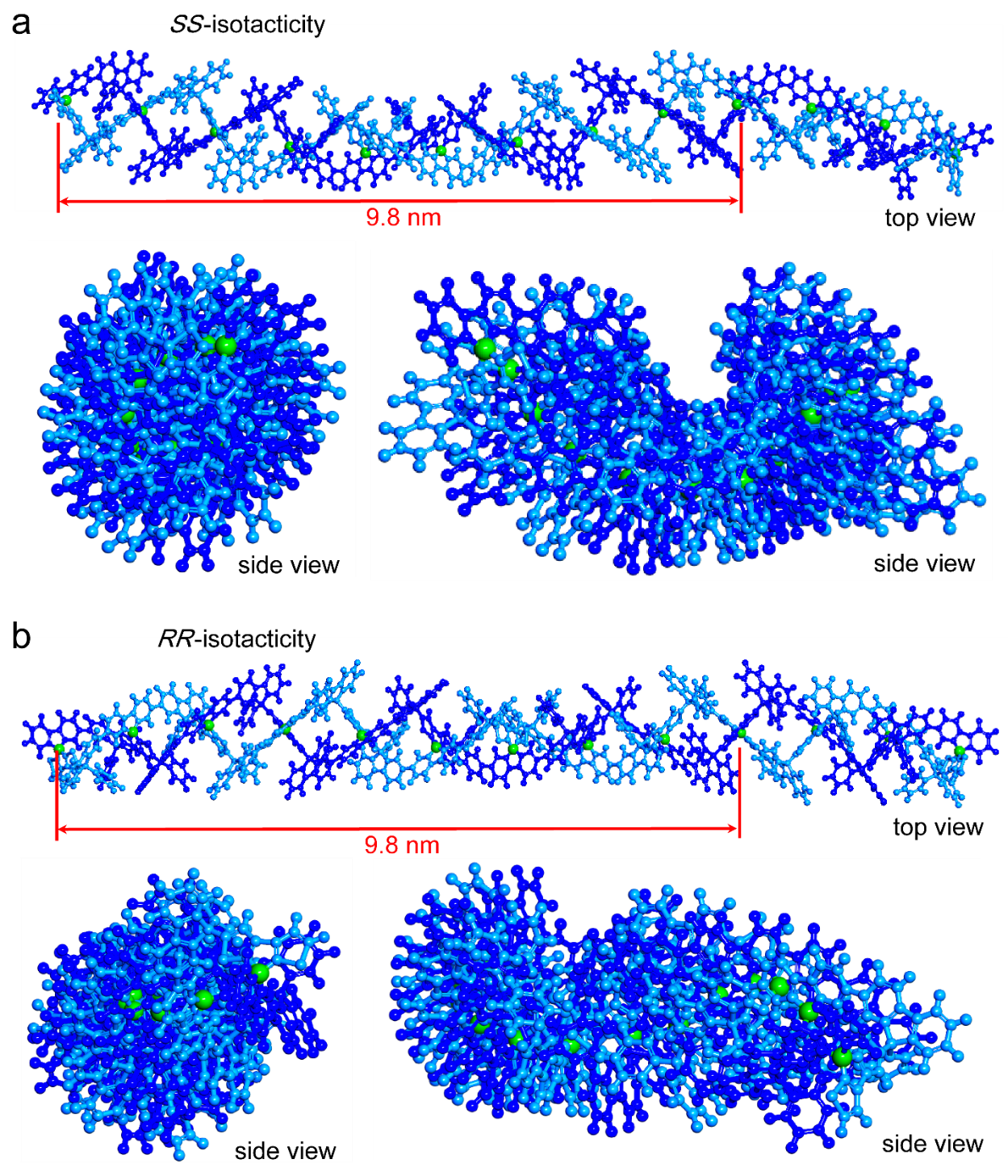


**Figure S7. The helical backbones of *SS*-isotactic and *RR*-isotacticity NPSG backbones (all in interlaced states).**

**4. The supplementary points of spiro-polygridization**


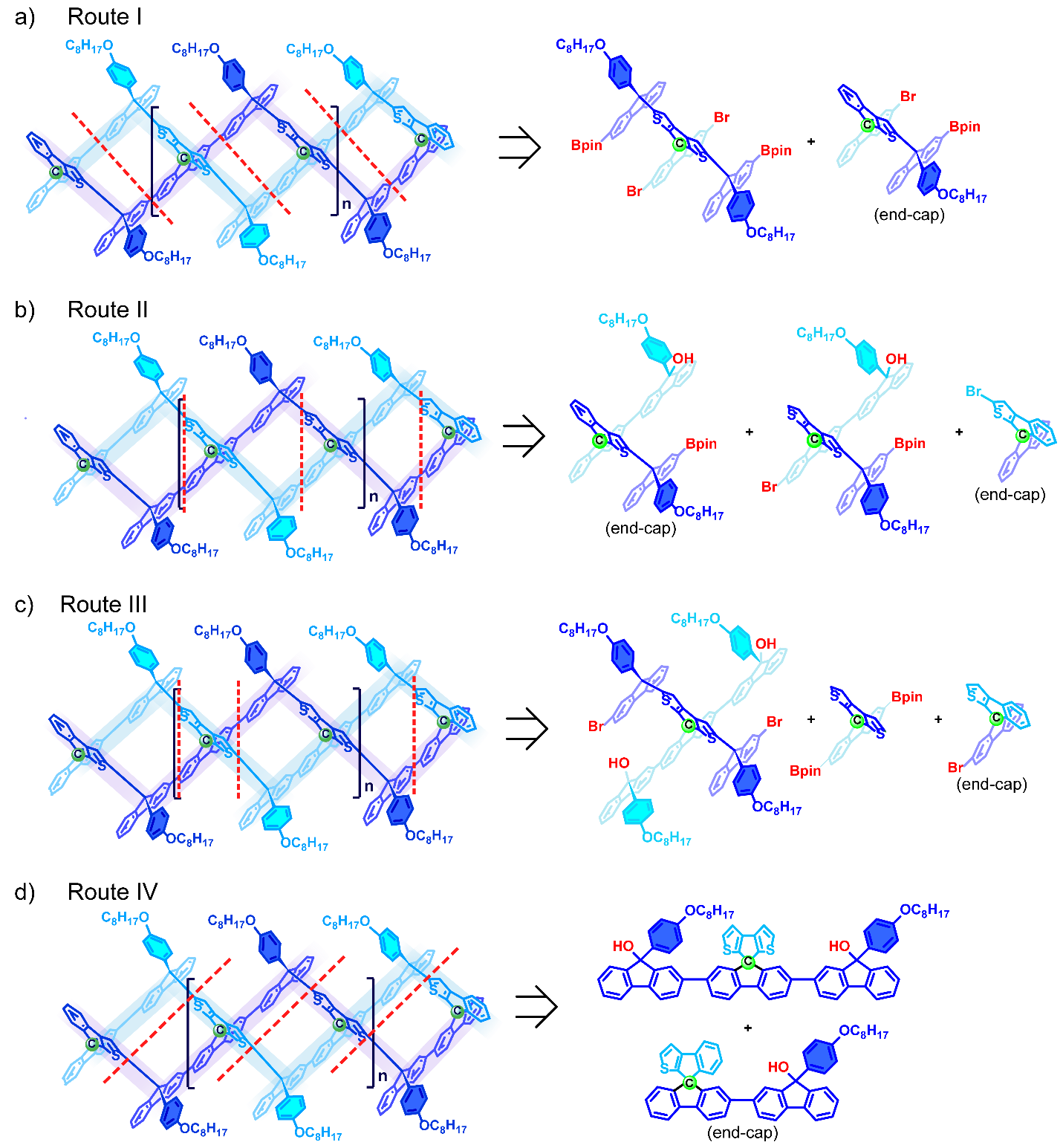


**Figure S8. The Retrosynthetic analysis of NPSG.** The red dashed lines represent the cleavage of C-C bonds.


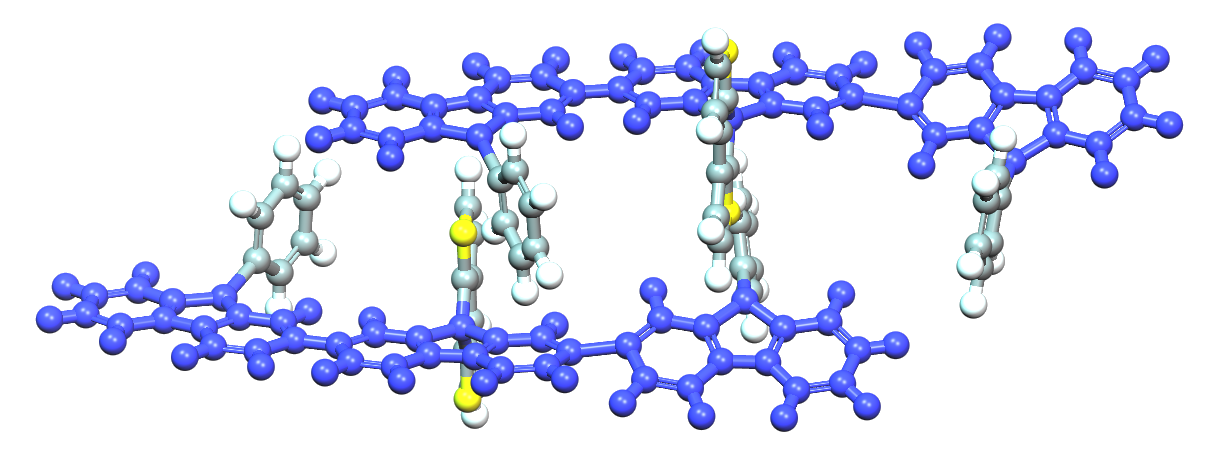


**Figure S9. Geometric matching model of STF-DOH intermediates (carbon cationic species).**


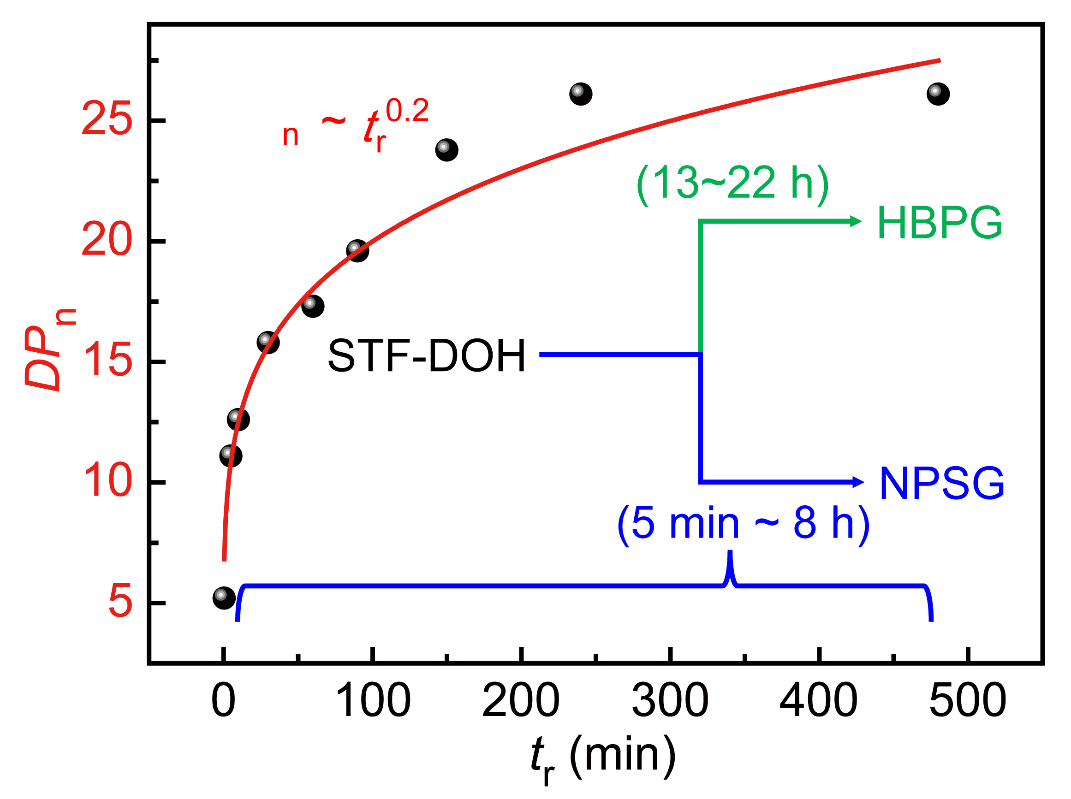


**Figure S10. The reaction time (*t*_r_) dependence of polygridization.** The concentration of STF-DOH (*C*_STF-DOH_) is fixed as 2 mM. The number-average degree of polymerization (*DP*_n_) was calculated based on the molecular weight calibration of NPSG oligomers (see below), for NPSG samples after Soxhlet extraction.


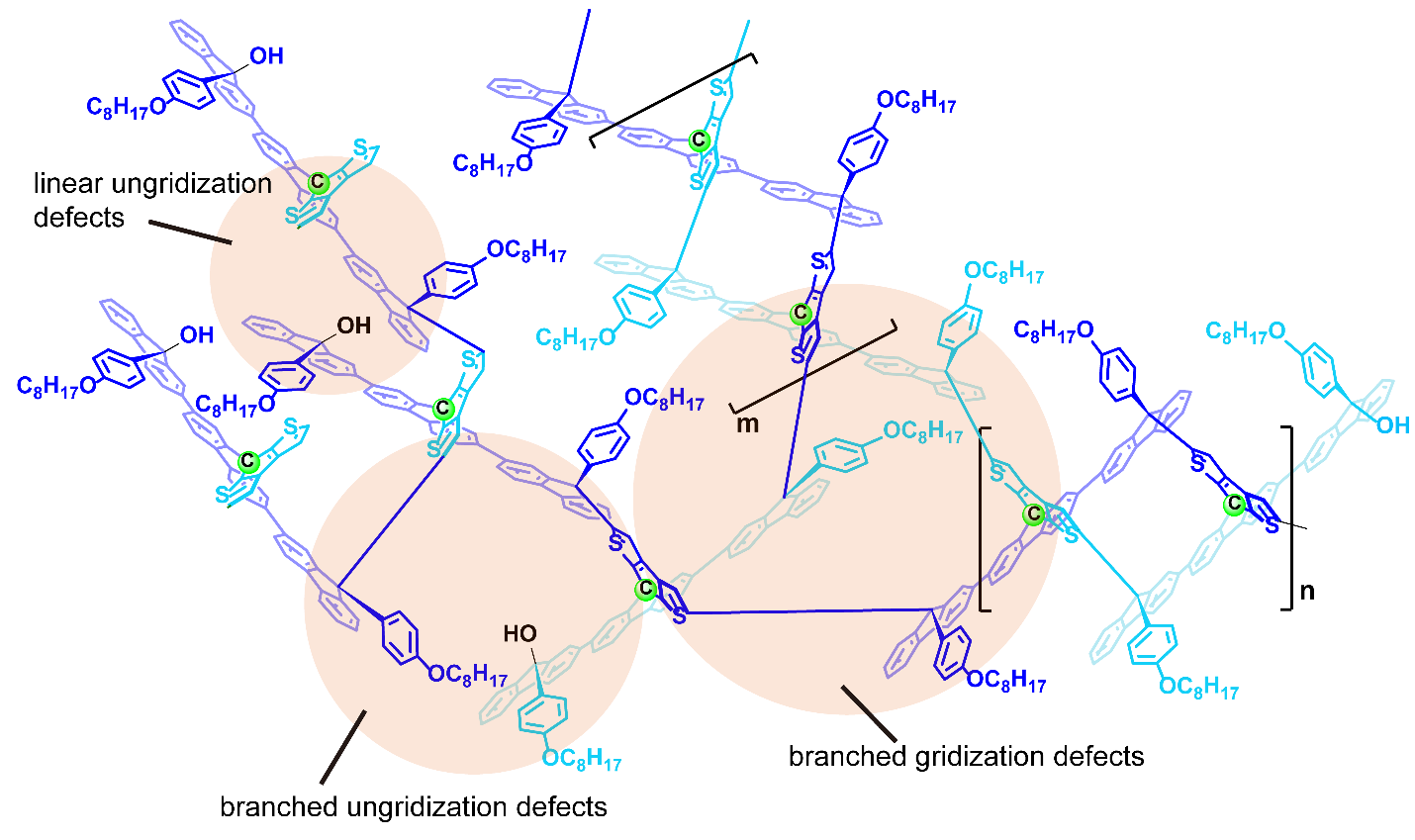


**Figure S11. The likely structure of hyperbranched polymer (HBPG) from the spiro-polygridization.** The structural defects such as the branched gridization defects, branched ungridization defects and linear ungridization defects are displayed in the shadow regions.


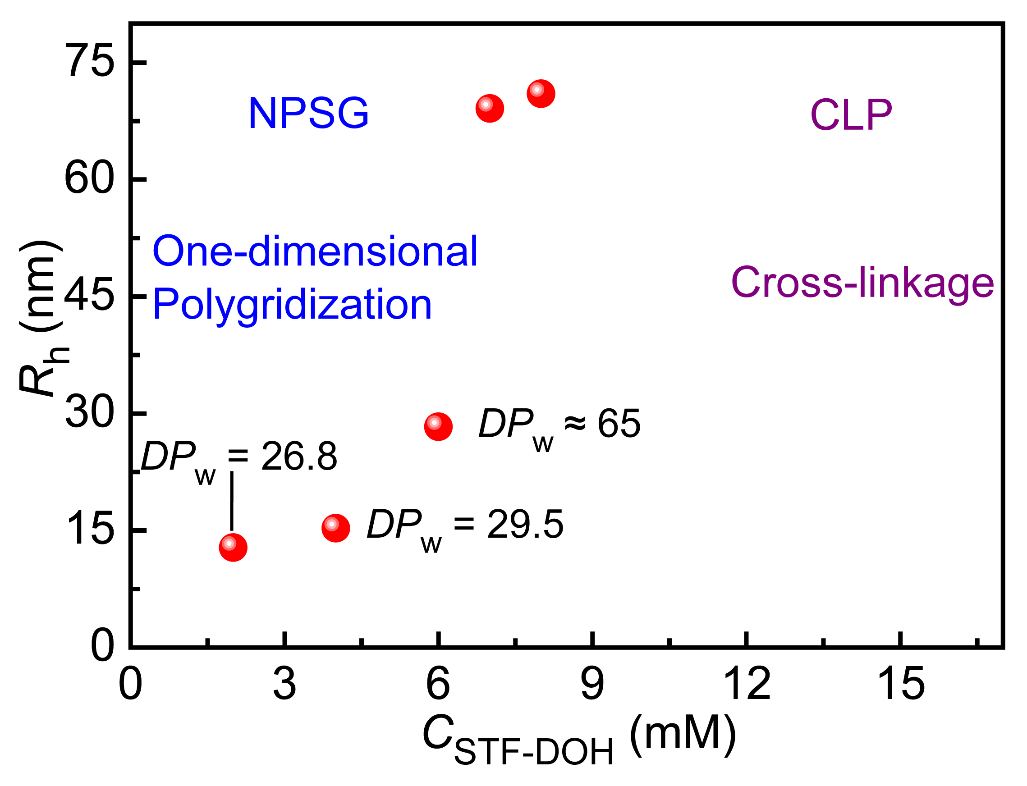


**Figure S12. The** ***C*_STF-DOH_ dependence of polygridization.** The reation time was set as *t*_r_ = 4h. The hydrodynamic radius were obtained via dynamic light scattering (see below). The weight-average degree of polymerization (*DP*_w_) was calculated based on the molecular weight calibration of NPSG oligomers (see below), for NPSG samples after Soxhlet extraction. For ***C*_STF-DOH_** = 7~8 mM, we cannot guarantee the linear structure of polygridized samples.

**5. Average degree of polymerization (*DP*) of NPSG nanochains**

Considering the single-chain properties of NPSG are drastically distinguished from the polystyrene (PS) systems, we calculated the number-average molecular weight (*M*_n_) and weight-average molecular weight (*M*_w_), transformed into number-average degree of polymerization (*DP*_n_) and weight-average degree of polymerization (*DP*_w_), based on the calibration of NPSG oligomers (*DP* = 2~5). The corresponding theories and details are carefully discussed in the literature [3]. Herein, we directly calculated their molecular weight calibration equation.


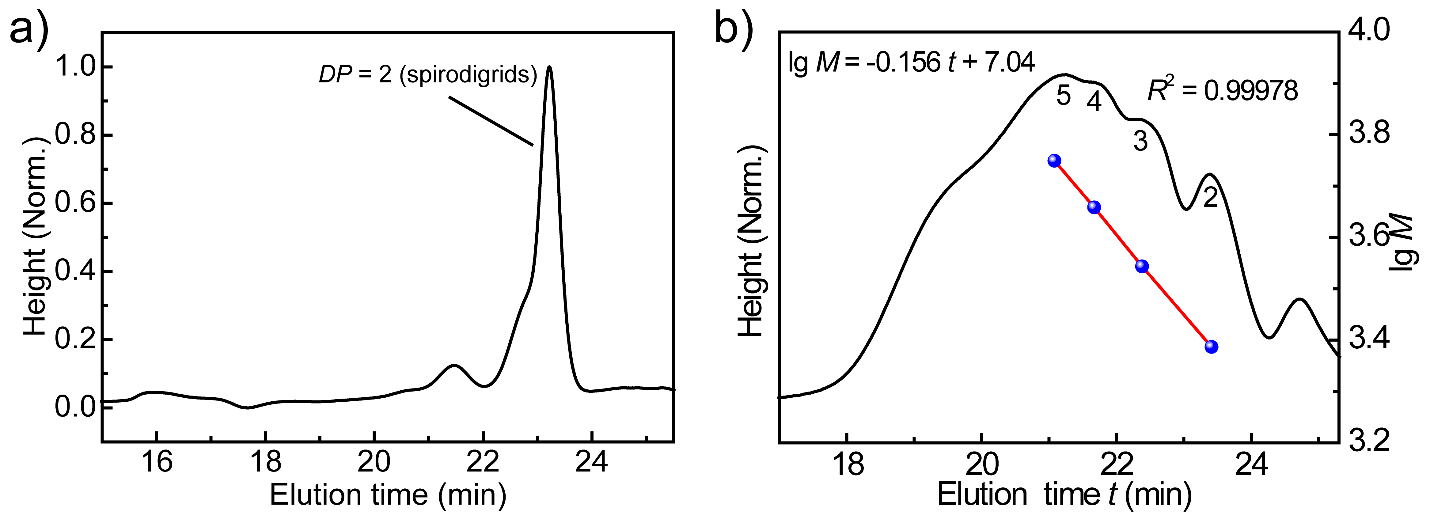


**Figure S13. The GPC spectra of spirodigrids (a) and the oligomers of NPSG (b).**

| *DP* | Elution time (min) | Exact mass (Da) | lg *M* |
| --- | --- | --- | --- |
| 2 | 23.41 | 2436 | 3.39 |
| 3 | 22.50 | 3496 | 3.54 |
| 4 | 21.67 | 4556 | 3.66 |
| 5 | 21.08 | 5616 | 3.75 |

**Table S1. The relationship between elution time and molar mass of NPSG oligomers.**

For the oligomer samples (0.5 min after the polymerization), we obtained their elution time (*t*) and corresponding molar mass (*M*) of NPSG oligomers via GPC spectra, in which the SDG (*DP* = 2 of NPSG oligomers) was isolated and reconfirmed at *t* = 23.41 min (Figure S13a, probably existing tiny deviations). On this basis, according to Figure S13b and Table S1, we constructed the molecular weight calibration equation ES1: lg *M* = -0.156 *t* + 7.04, with the coefficient of determination *R*^2^ = 0.99978.

In terms of the calibration equation, its slope *k*_NPSG_ = -0.156 can be transformed into the Mark-Houwink exponent *α*_NPSG_ = 1.175 (for only oligomers *DP* = 2~5), based on the relationship ES2: (*α*_NPSG_ +1)/ (*α*_PS_ +1) = *k*_PS_ /k_NPSG_, where *k*_PS_ = -0.198 and *α*_PS_ = 0.714 for PS systems in THF solvent [*10*].

To further calibrate higher *DP*s of NPSG chains, we extrapolated its calibration equation that is listed in Table S2.

| *DP* | Elution time (min) | Exact mass (Da) | lg *M* |
| --- | --- | --- | --- |
| 6 | 20.64 | 6676 | 3.82452 |
| 7  8  9  10  11  12  13  15  17  20  23  25  27  30  32  35  40 | 20.18  19.82  19.51  19.22  18.96  18.73  18.51  18.12  17.80  17.33  16.95  16.72  16.50  16.24  16.04  15.79  15.42 | 7796  8866  9936  11006  12076  13136  14196  16356  18336  21706  24916  27056  29196  32116  34546  37756  43016 | 3.89187  3.94773  3.99721  4.04163  4.08192  4.11846  4.15217  4.21368  4.2633  4.33658  4.39648  4.43226  4.46532  4.50672  4.5384  4.57699  4.63363 |
| 45 | 15.09 | 48456 | 4.68535 |

**Table S2. The elution time of NPSG with individual molar mass (extrapolated via its calibration equation)**


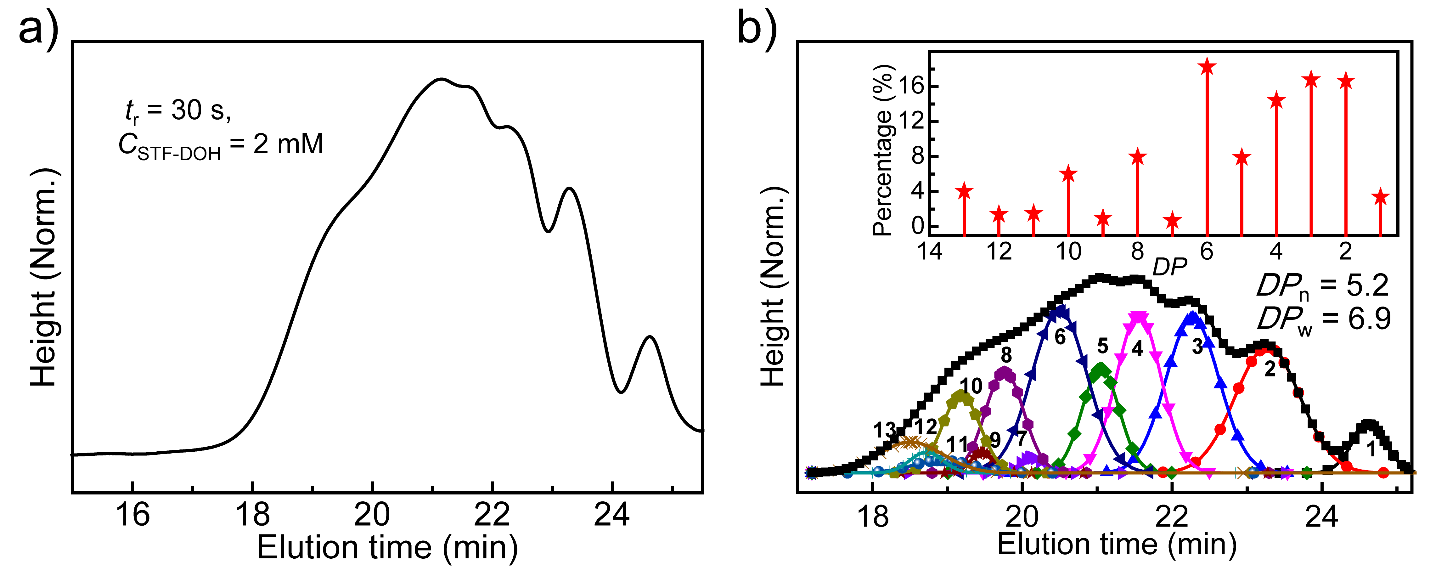


**Figure S14. The GPC spectra of NPSG (oligomers that obtained from the polygridization under the conditions of *t*_r_ = 30 s and *C*_STF-DOH_ = 2 mM).** (a) Raw data. (b) Detailed analysis. The total signal peak is divided into gauss peaks with individually pure values, based on the extrapolation of calibration equation (Table S2). The height of red stars is defined as the percentage of individual *DP* values. The integration of each *DP* (with individual percentage) enables to afford *DP*_n_ and *DP*_w_ respectively, which are also provided.


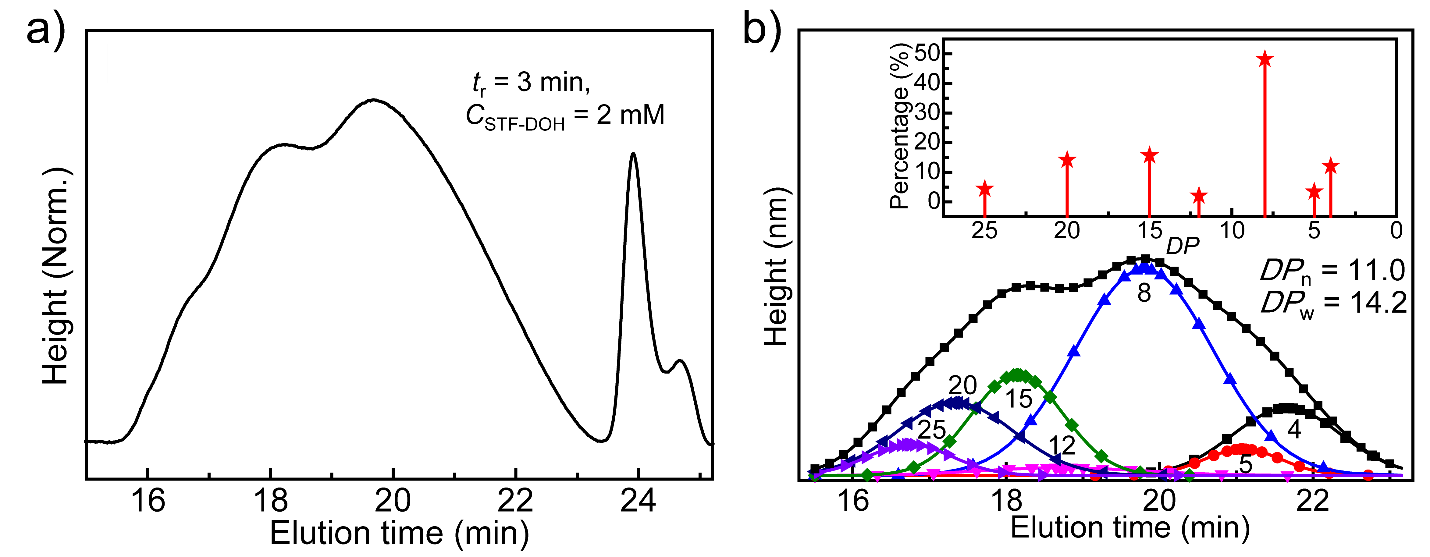


**Figure S15. The GPC spectra of NPSG (from the polygridization under the conditions of *t*_r_ = 3 min and *C*_STF-DOH_ = 2 mM).** (a) Raw data. (b) Detailed analysis. The total signal peak is divided into gauss peaks with individually pure values, based on the extrapolation of calibration equation (Table S2). The height of red stars is defined as the percentage of individual *DP* values. The integration of each *DP* (with individual percentage) enables to afford *DP*_n_ and *DP*_w_ respectively, which are also provided.


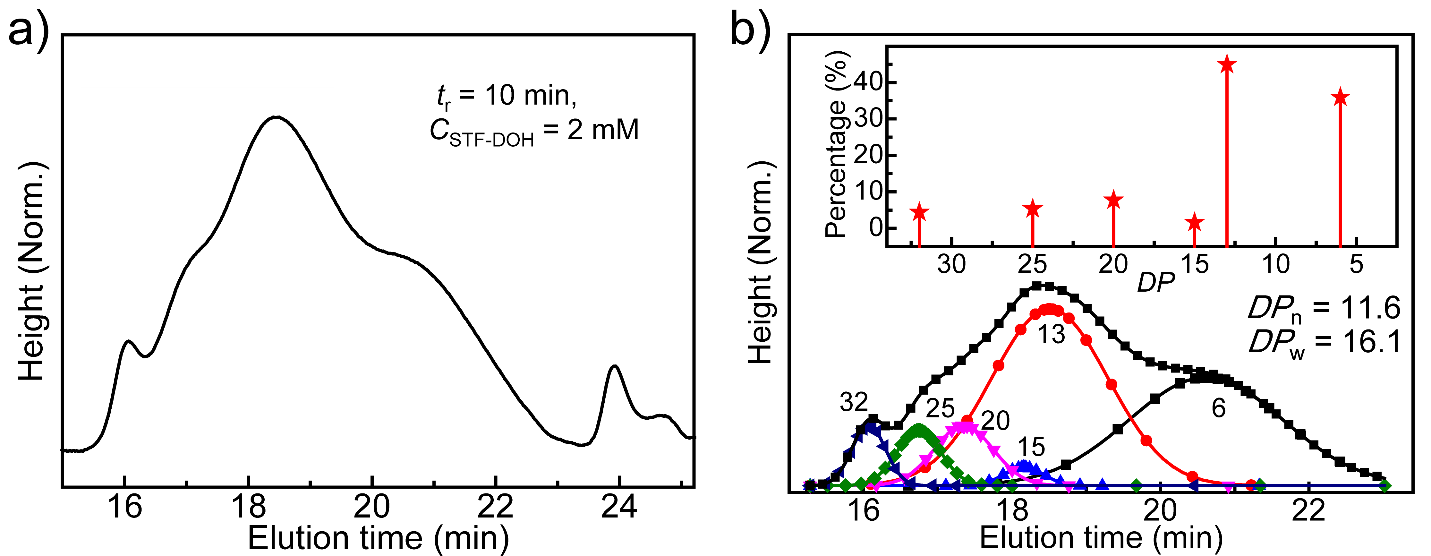


**Figure S16. The GPC spectra of NPSG (from the polygridization under the conditions of *t*_r_ = 10 min and *C*_STF-DOH_ = 2 mM).** (a) Raw data. (b) Detailed analysis. The total signal peak is divided into gauss peaks with individually pure values, based on the extrapolation of calibration equation (Table S2). The height of red stars is defined as the percentage of individual *DP* values. The integration of each *DP* (with individual percentage) enables to afford *DP*_n_ and *DP*_w_ respectively, which are also provided. **It is noted that the above *DP* values are average values, not the monodisperse values**.


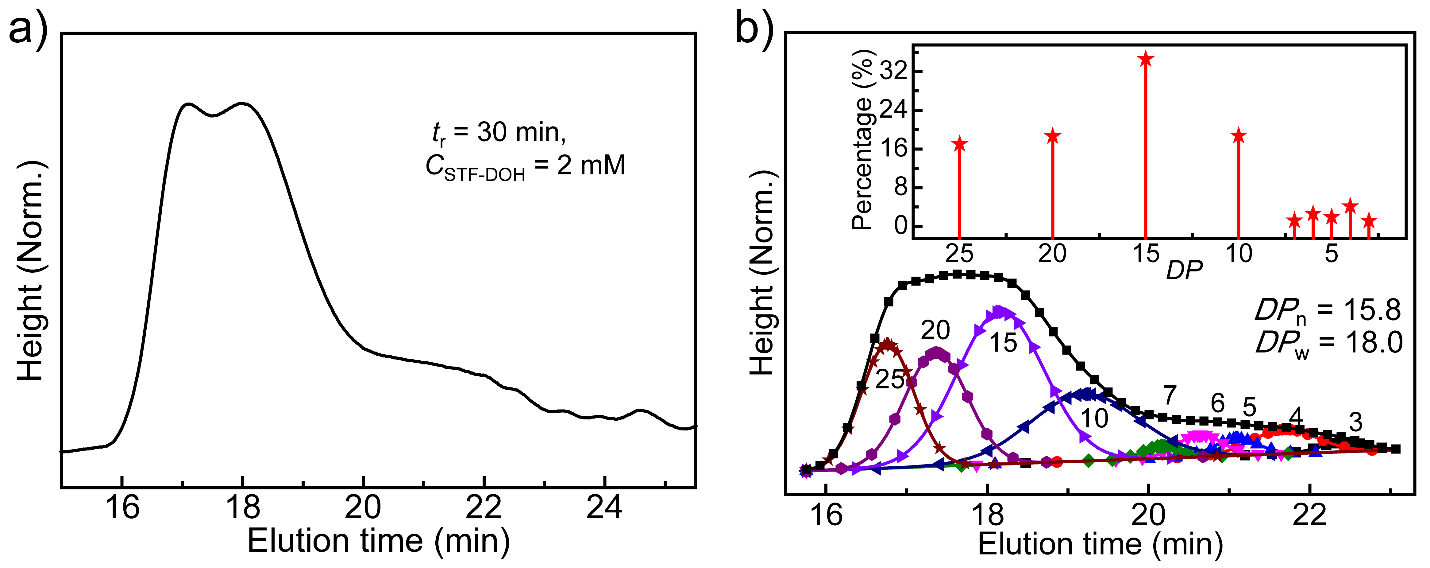


**Figure S17. The GPC spectra of NPSG (from the polygridization under the conditions of *t*_r_ = 30 min and *C*_STF-DOH_ = 2 mM).** (a) Raw data. (b) Detailed analysis. The total signal peak is divided into gauss peaks with individually pure values, based on the extrapolation of calibration equation (Table S2). The height of red stars is defined as the percentage of individual *DP* values. The integration of each *DP* (with individual percentage) enables to afford *DP*_n_ and *DP*_w_ respectively, which are also provided. **It is noted that the *DP* values of 10, 15, 20 and 25 are average values, not the monodisperse values**.


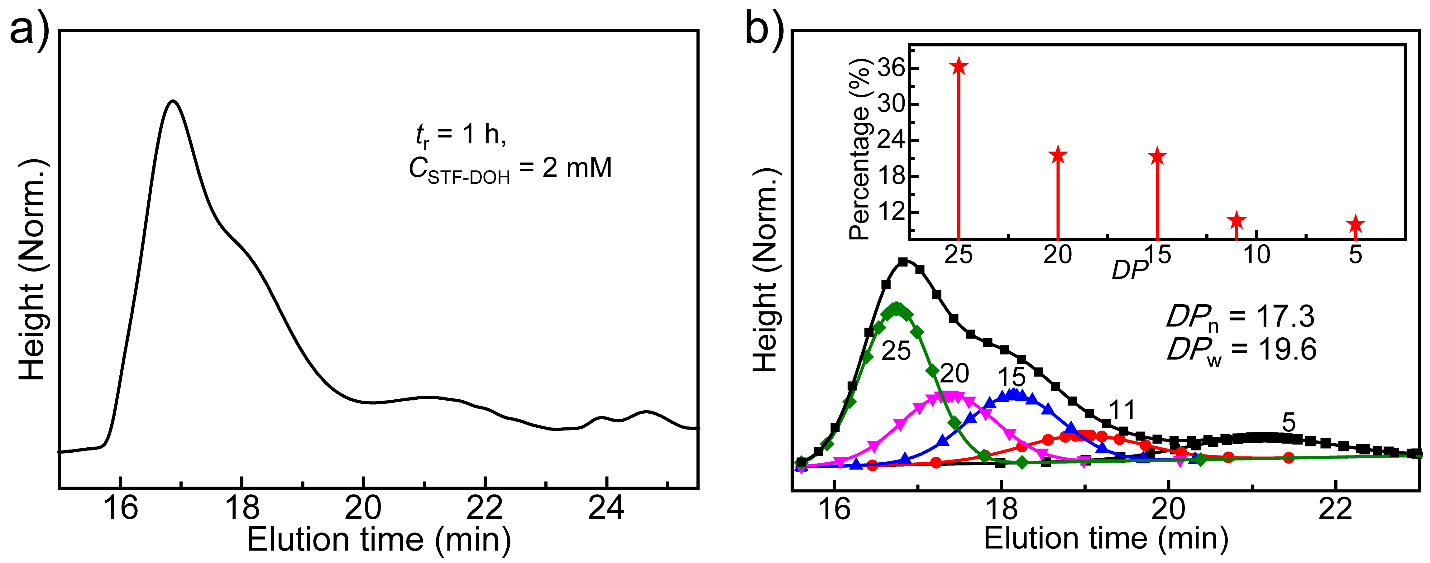


**Figure S18. The GPC spectra of NPSG (from the polygridization under the conditions of *t*_r_ = 1 h and *C*_STF-DOH_ = 2 mM).** (a) Raw data. (b) Detailed analysis. The total signal peak is divided into gauss peaks with individually pure values, based on the extrapolation of calibration equation (Table S2). The height of red stars is defined as the percentage of individual *DP* values. The integration of each *DP* (with individual percentage) enables to afford *DP*_n_ and *DP*_w_ respectively, which are also provided. **It is noted that the *DP* values of 5, 11, 15, 20 and 25 are average values, not the monodisperse values**.


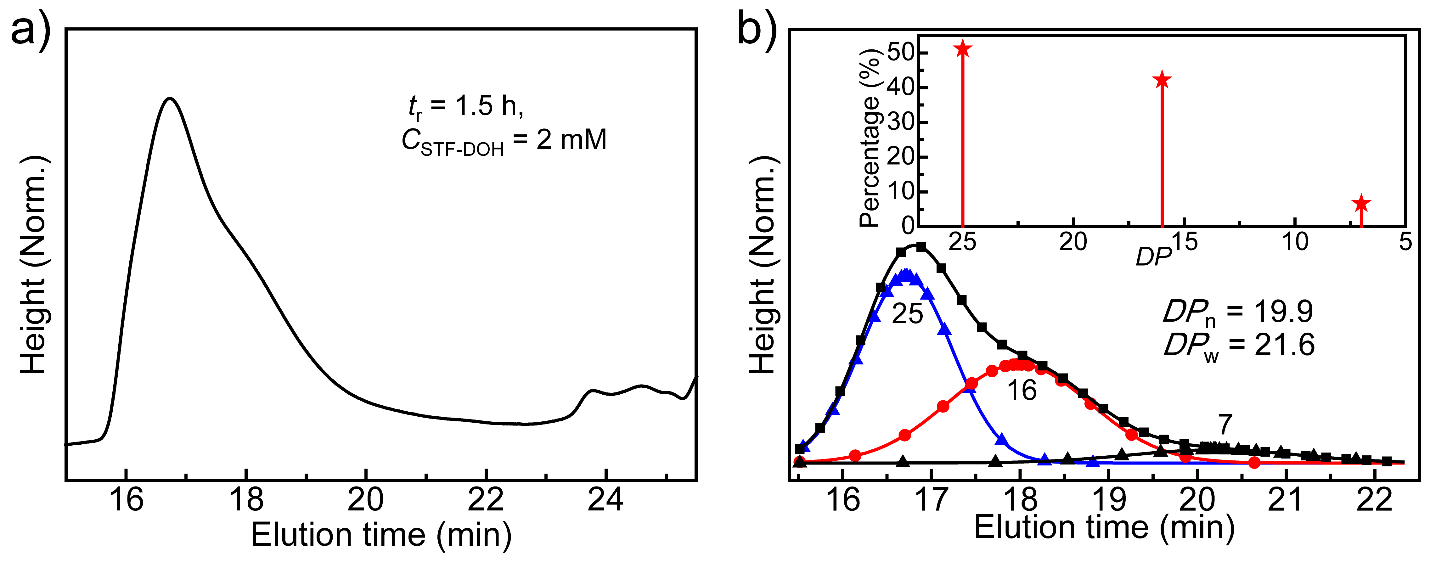


**Figure S19. The GPC spectra of NPSG (from the polygridization under the conditions of *t*_r_ = 1.5 h and *C*_STF-DOH_ = 2 mM).** (a) Raw data. (b) Detailed analysis. The total signal peak is divided into gauss peaks with individually pure values, based on the extrapolation of calibration equation (Table S2). The height of red stars is defined as the percentage of individual *DP* values. The integration of each *DP* (with individual percentage) enables to afford *DP*_n_ and *DP*_w_ respectively, which are also provided. **It is noted that the *DP* values of 25, 16 and 7 are average values, not the monodisperse values**.


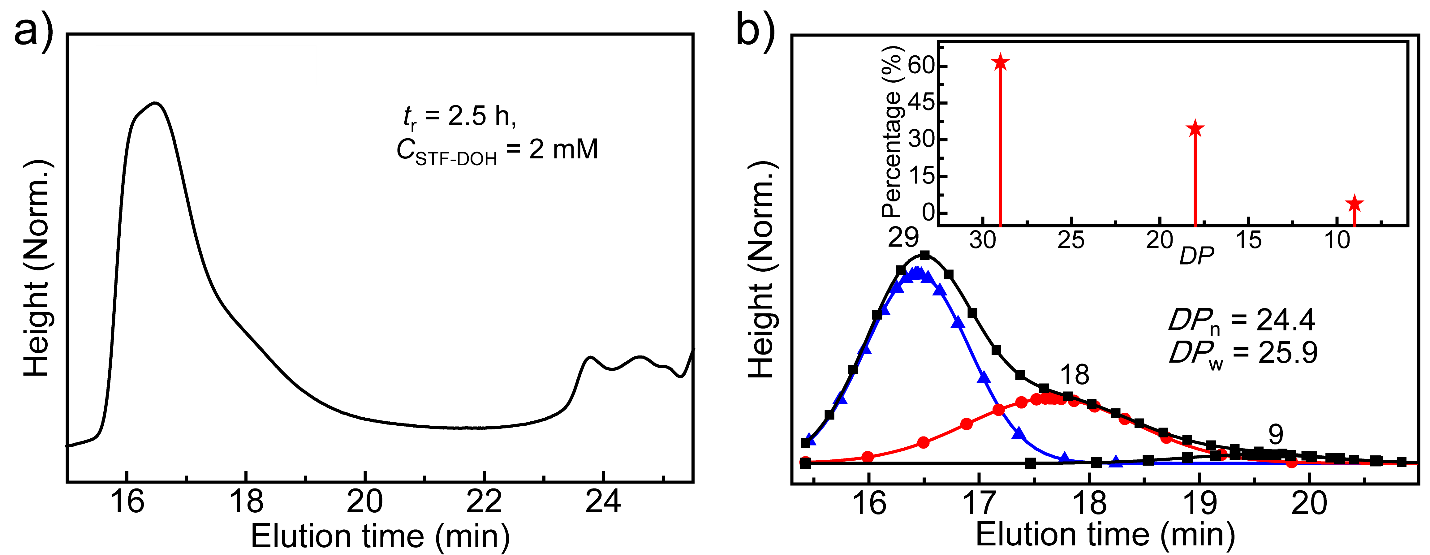


**Figure S20. The GPC spectra of NPSG (from the polygridization under the conditions of *t*_r_ = 2.5 h and *C*_STF-DOH_ = 2 mM).** (a) Raw data. (b) Detailed analysis. The total signal peak is divided into gauss peaks with individually pure values, based on the extrapolation of calibration equation (Table S2). The height of red stars is defined as the percentage of individual *DP* values. The integration of each *DP* (with individual percentage) enables to afford *DP*_n_ and *DP*_w_ respectively, which are also provided. **It is noted that the *DP* values of 29, 18 and 9 are average values, not the monodisperse values**.


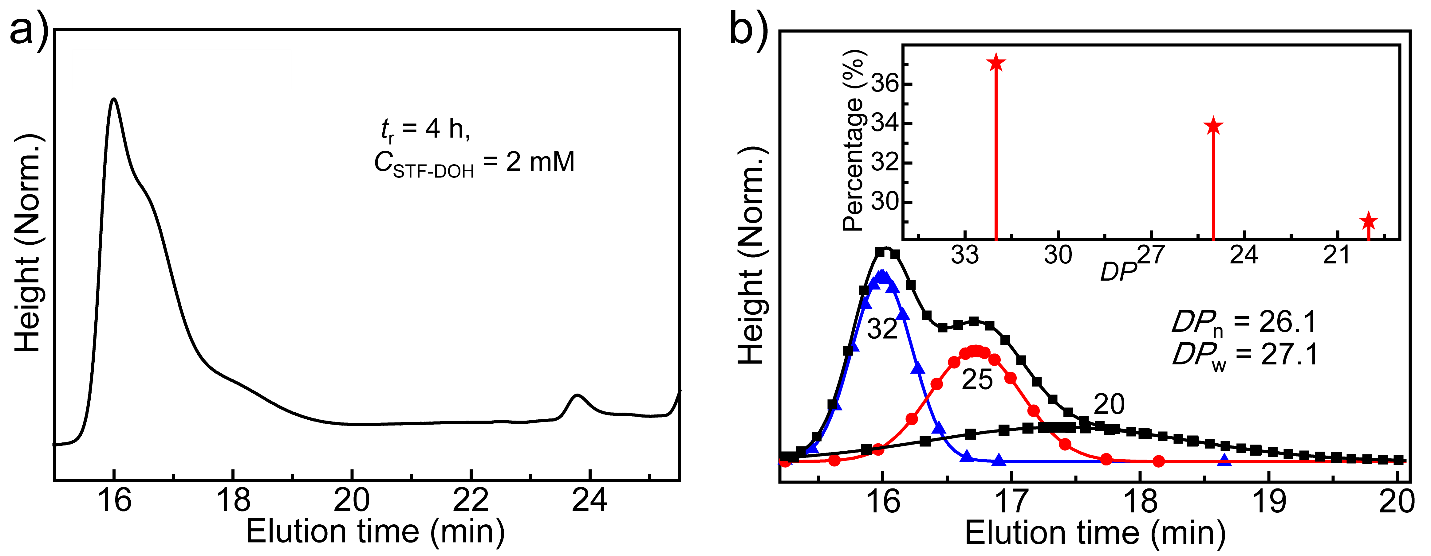


**Figure S21. The GPC spectra of NPSG (from the polygridization under the conditions of *t*_r_ = 4 h and *C*_STF-DOH_ = 2 mM).** (a) Raw data. (b) Detailed analysis. The total signal peak is divided into gauss peaks with individually pure values, based on the extrapolation of calibration equation (Table S2). The height of red stars is defined as the percentage of individual *DP* values. The integration of each *DP* (with individual percentage) enables to afford *DP*_n_ and *DP*_w_ respectively, which are also provided. **It is noted that the *DP* values of 32, 25 and 20 are average values, not the monodisperse values**.


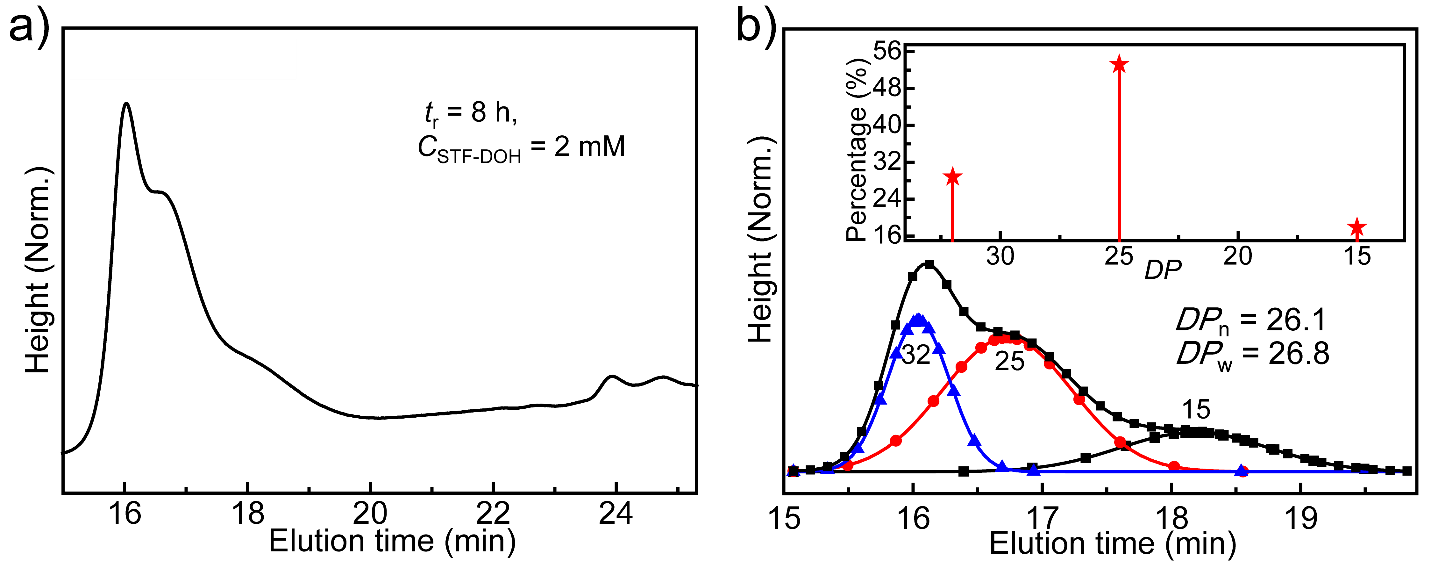


**Figure S22. The GPC spectra of NPSG (from the polygridization under the conditions of *t*_r_ = 8 h and *C*_STF-DOH_ = 2 mM).** (a) Raw data. (b) Detailed analysis. The total signal peak is divided into gauss peaks with individually pure values, based on the extrapolation of calibration equation (Table S2). The height of red stars is defined as the percentage of individual *DP* values. The integration of each *DP* (with individual percentage) enables to afford *DP*_n_ and *DP*_w_ respectively, which are also provided. **It is noted that the *DP* values of 32, 25 and 15 are average values, not the monodisperse values**.


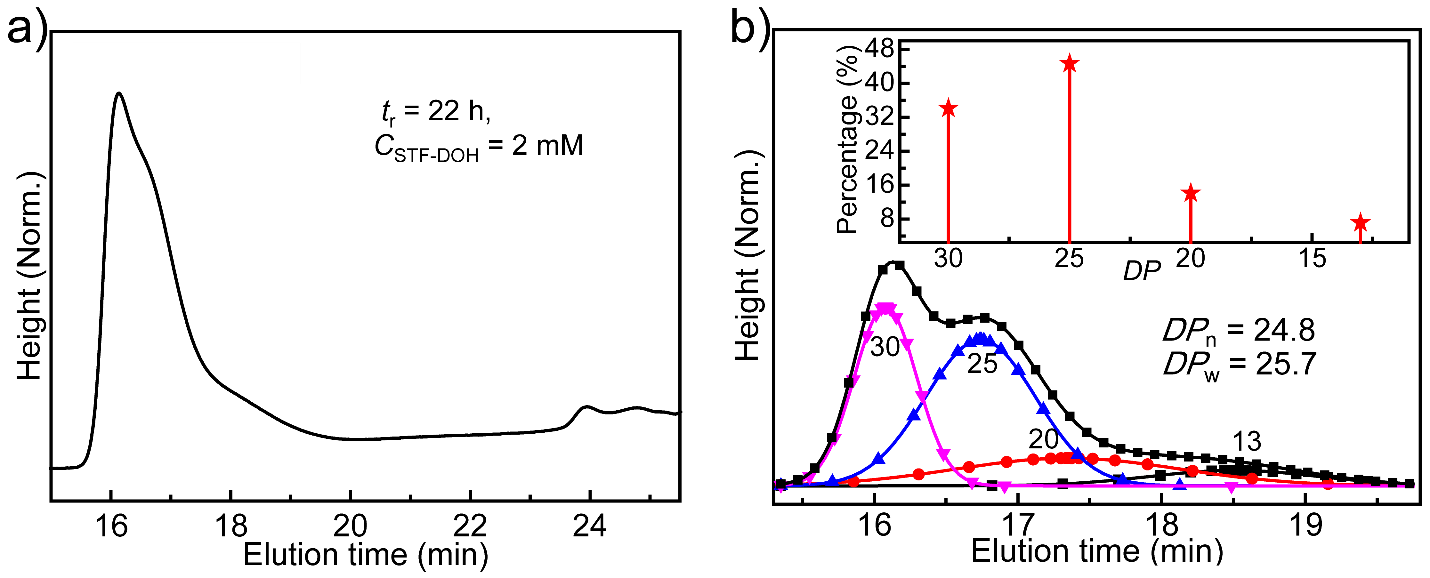


**Figure S23. The GPC spectra of NPSG (from the polygridization under the conditions of *t*_r_ = 22 h and *C*_STF-DOH_ = 2 mM).** (a) Raw data. (b) Detailed analysis. The total signal peak is divided into gauss peaks with individually pure values, based on the extrapolation of calibration equation (Table S2). The height of red stars is defined as the percentage of individual *DP* values. The integration of each *DP* (with individual percentage) enables to afford *DP*_n_ and *DP*_w_ respectively, which are also provided. **It is noted that the *DP* values of 30, 25, 20 and 15 are average values, not the monodisperse values**. **In fact, for this sample, its hydrodynamic radius is evidently unmatched with this GPC results. Maybe its molecular weight excess the scope of GPC technology.**


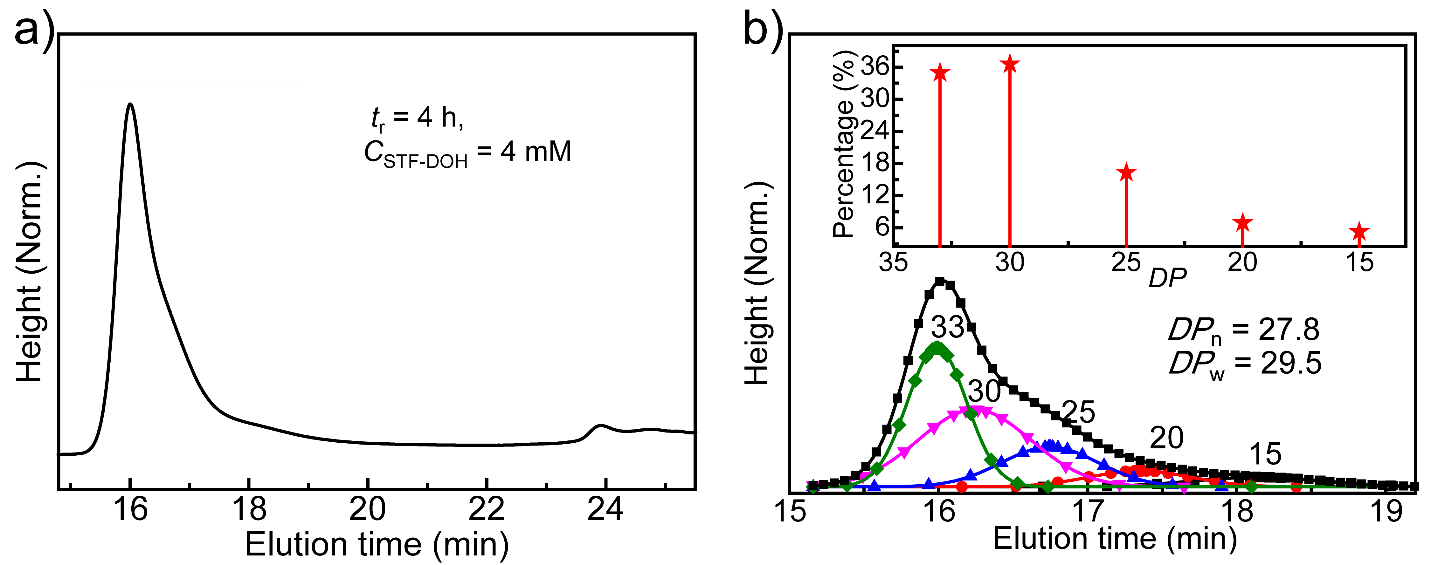


**Figure S24. The GPC spectra of NPSG (from the polygridization under the conditions of *t*_r_ = 4 h and *C*_STF-DOH_ = 4 mM).** (a) Raw data. (b) Detailed analysis. The total signal peak is divided into gauss peaks with individually pure values, based on the extrapolation of calibration equation (Table S2). The height of red stars is defined as the percentage of individual *DP* values. The integration of each *DP* (with individual percentage) enables to afford *DP*_n_ and *DP*_w_ respectively, which are also provided. **It is noted that the *DP* values of 33, 30, 25, 20 and 15 are average values, not the monodisperse values**.


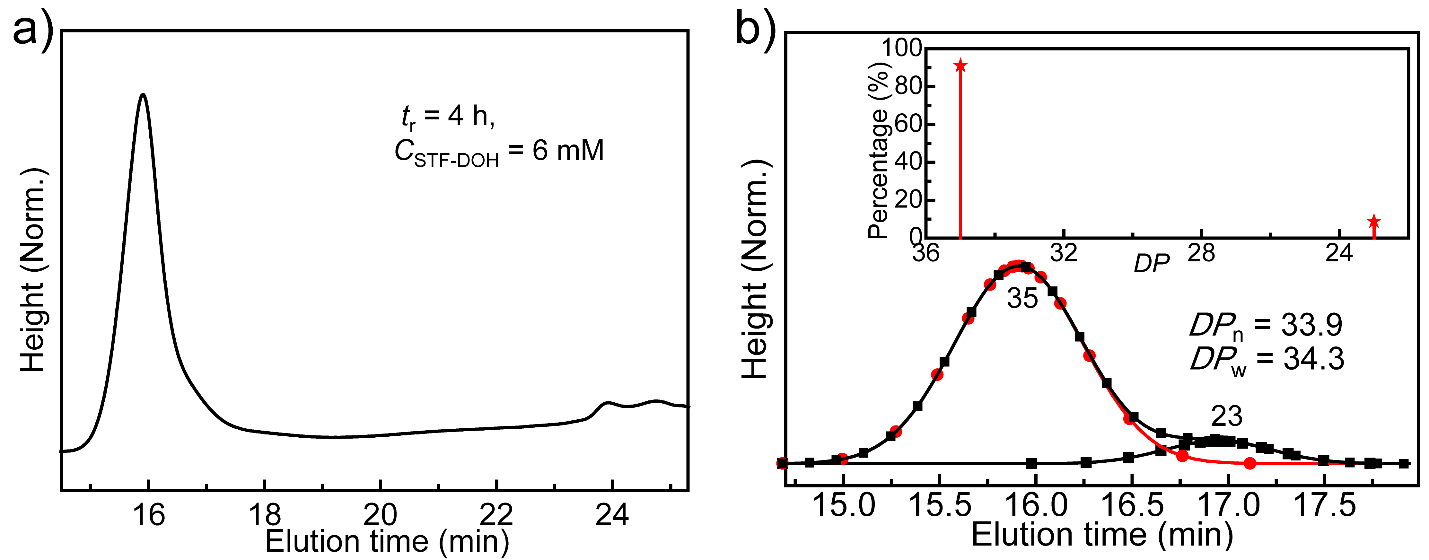


**Figure S25. The GPC spectra of NPSG (from the polygridization under the conditions of *t*_r_ = 4 h and *C*_STF-DOH_ = 6 mM).** (a) Raw data. (b) Detailed analysis. The total signal peak is divided into gauss peaks with individually pure values, based on the extrapolation of calibration equation (Table S2). The height of red stars is defined as the percentage of individual *DP* values. The integration of each *DP* (with individual percentage) enables to afford *DP*_n_ and *DP*_w_ respectively, which are also provided. **It is noted that the *DP* values of 35 and 23 are average values, not the monodisperse values**. **In fact, for this sample, its hydrodynamic radius is evidently unmatched with this GPC results. Maybe its molecular weight excess the scope of GPC technology.**


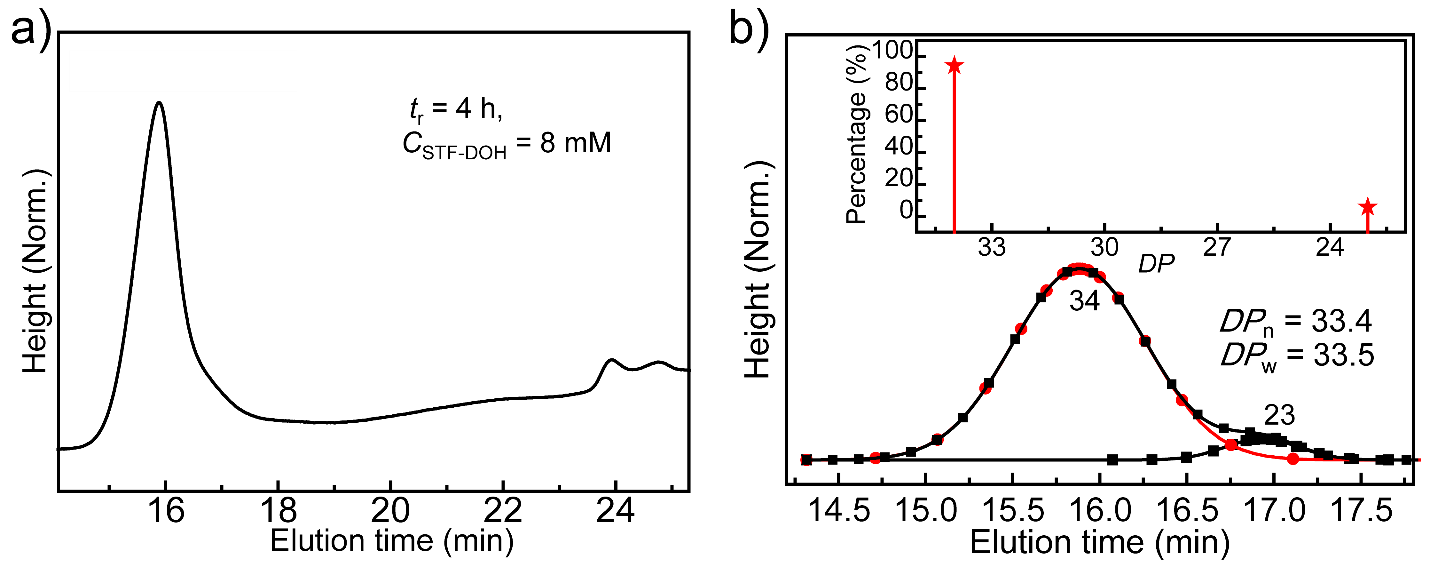


**Figure S26. The GPC spectra of NPSG (from the polygridization under the conditions of *t*_r_ = 4 h and *C*_STF-DOH_ = 8 mM).** (a) Raw data. (b) Detailed analysis. The total signal peak is divided into gauss peaks with individually pure values, based on the extrapolation of calibration equation (Table S2). The height of red stars is defined as the percentage of individual *DP* values. The integration of each *DP* (with individual percentage) enables to afford *DP*_n_ and *DP*_w_ respectively, which are also provided. **It is noted that the *DP* values of 34 and 23 are average values, not the monodisperse values**. **In fact, for this sample, its hydrodynamic radius is evidently unmatched with this GPC results. Maybe its molecular weight excess the scope of GPC technology.**

**6. Characterizations of NPSG chains**


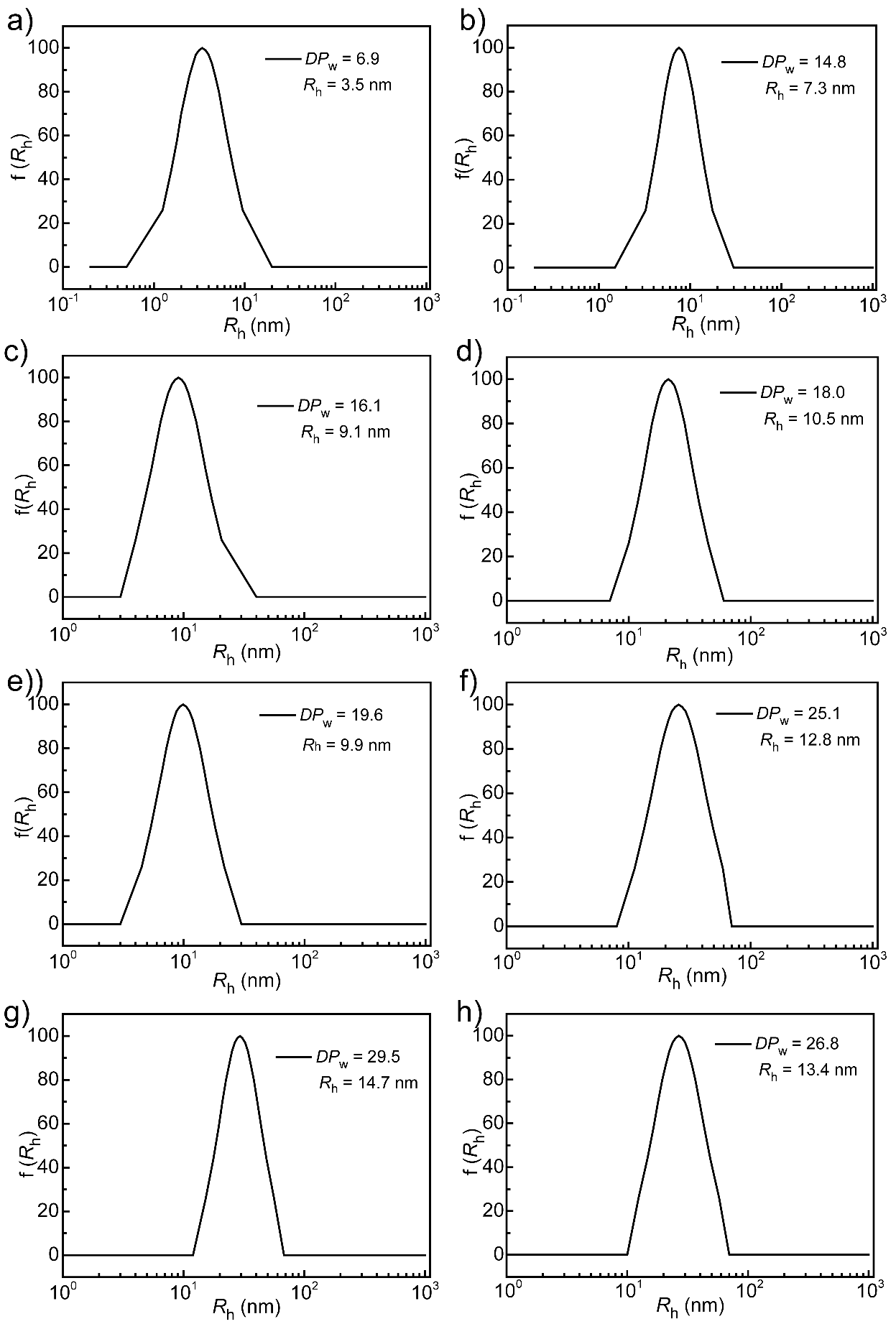


**Figure S27. The hydrodynamic radius (*R*_h_) of NPSG with various *DP*_w_.** It is noted that the chain sizes *L* are evaluated via *L* = 2 *R*_h_ in CHCl_3_ solvent.


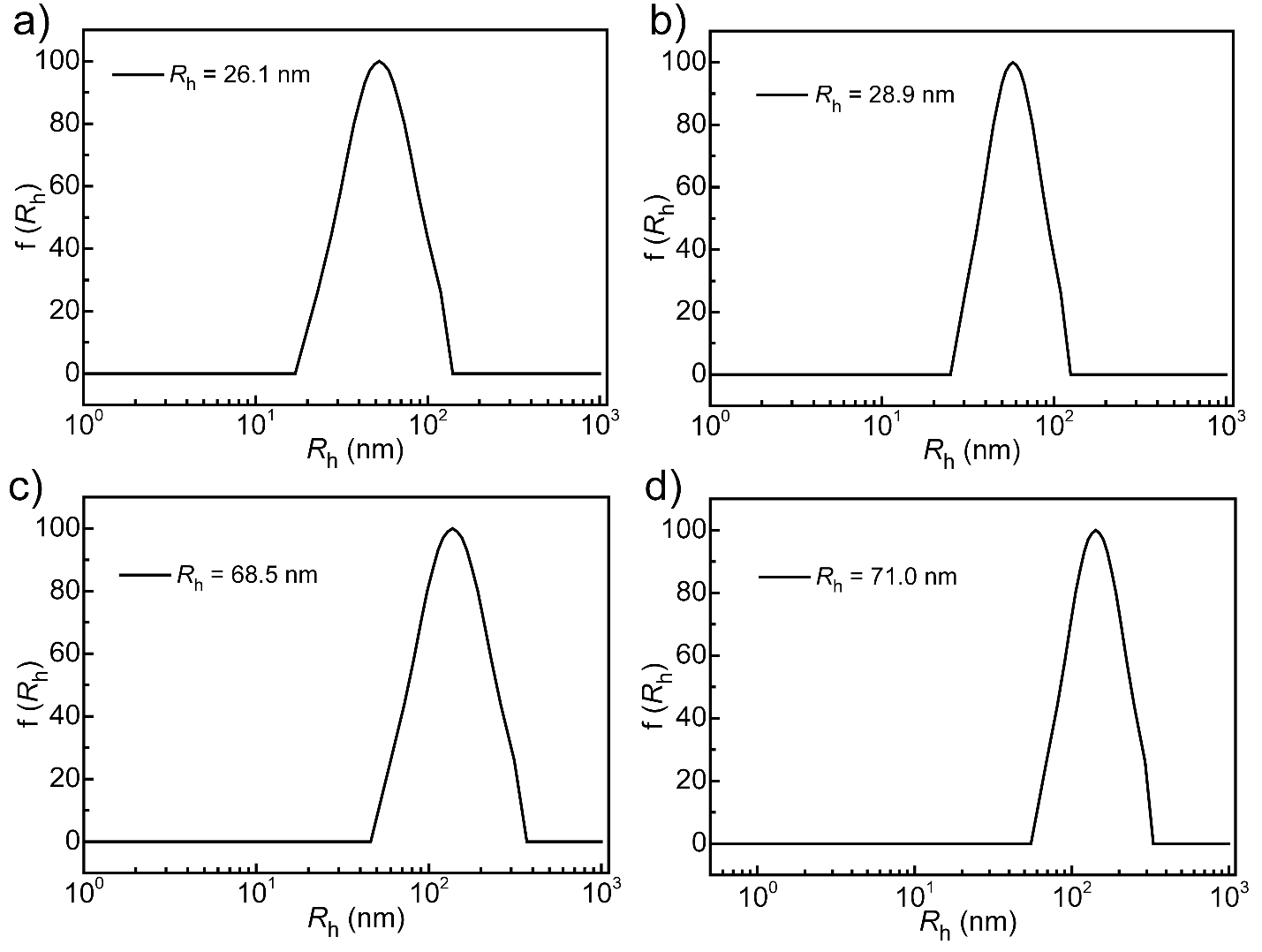


**Figure S28. The hydrodynamic radius (*R*_h_) of NPSG that evidently larger than the chain sizes which calculated from the GPC analysis.** (a) This sample originates from the *t*_r_ = 22 h after the polygridization (its GPC spectra is shown in Figure S22). By further study (See below), this backbone is the branched HBPG. (b) This sample is obtained from *C*_STF-DOH_ = 6 mM during the polygridization (Figure S24). The deep study (see below) reveals the linear rod-like configurations (ultralong NPSG chains). (c) and (d) are derived from *C*_STF-DOH_ = 7 mM and 8 mM, respectively, during the polygridization. We only observed their chain length can be up to 140 nm but we cannot testify their linear configurations due to the evidently lowered solubility. It is noted that even further diluting the concentration of above samples, their detected *R*_h_ values were maintained as well, which suggests the intrinsic sizes rather than the aggregation.


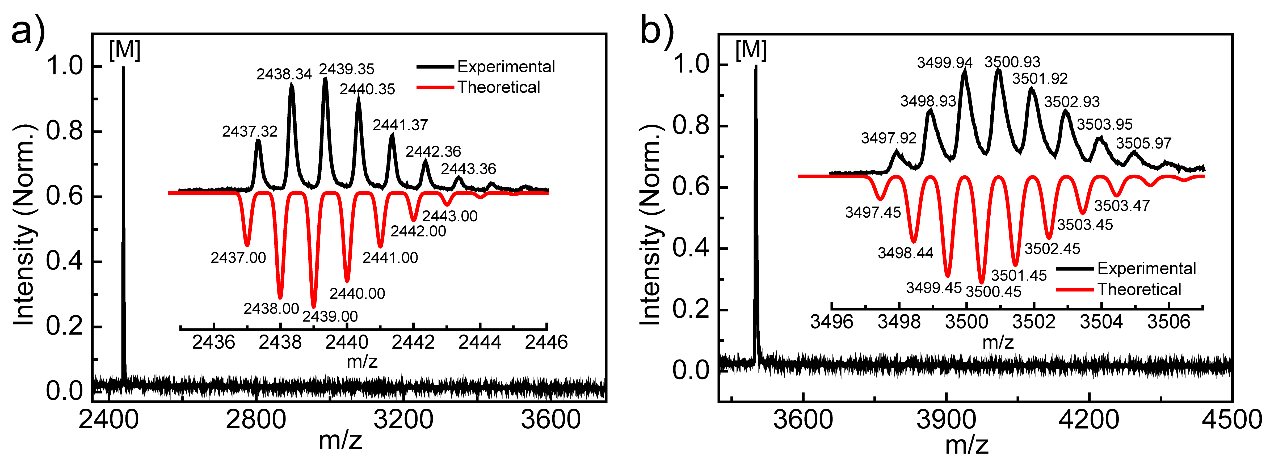


**Figure S29. MS spectra of SDG (a) and spirotrigrid (b)**. The simulation of isotopic distributions (in red lines) are also provided.


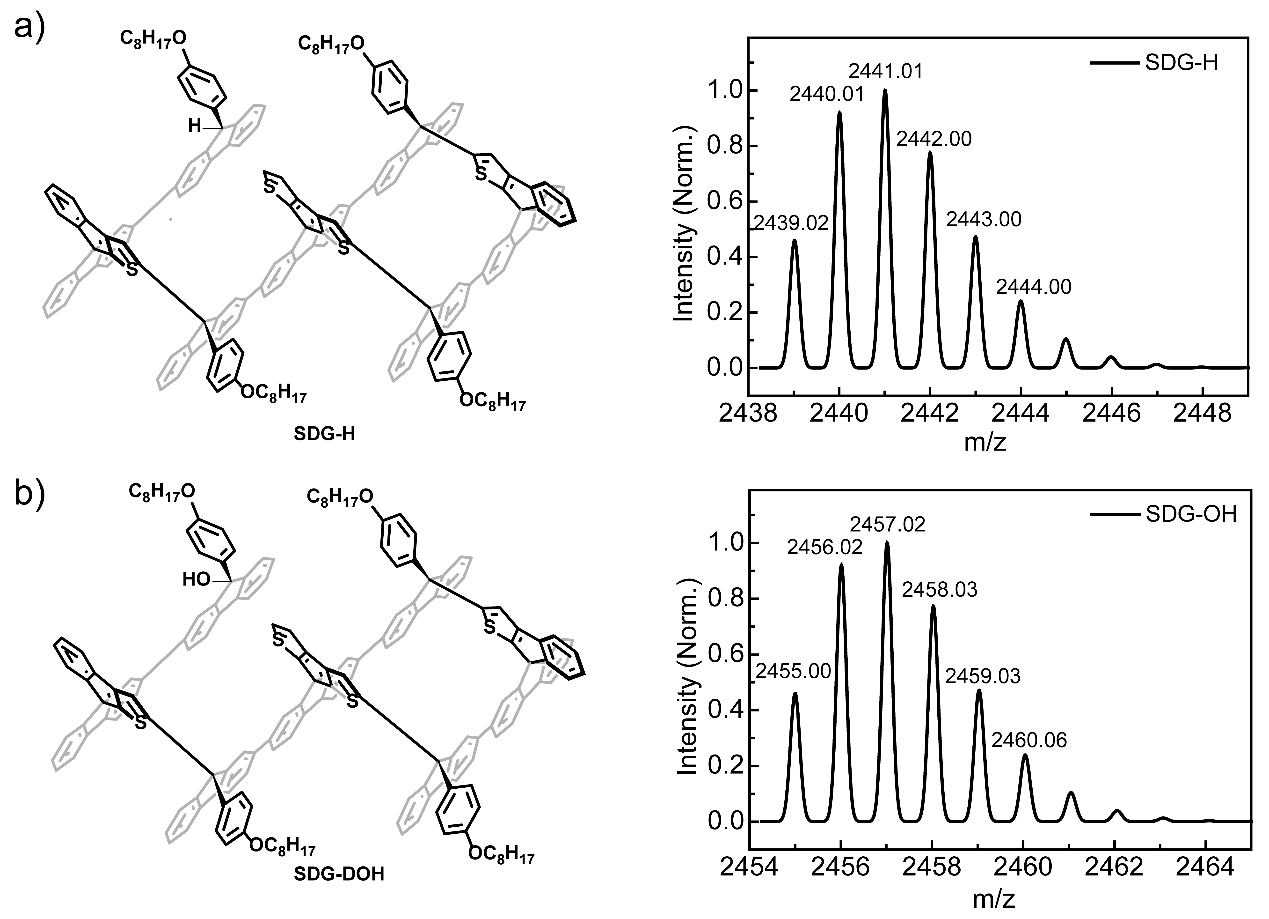


**Figure S30. MS spectra simulation of ungridized defective SDG structures.** (a) dehydroxylated byproduct SDG-H. (b) hydroxyl-containing byproduct SDG-OH.


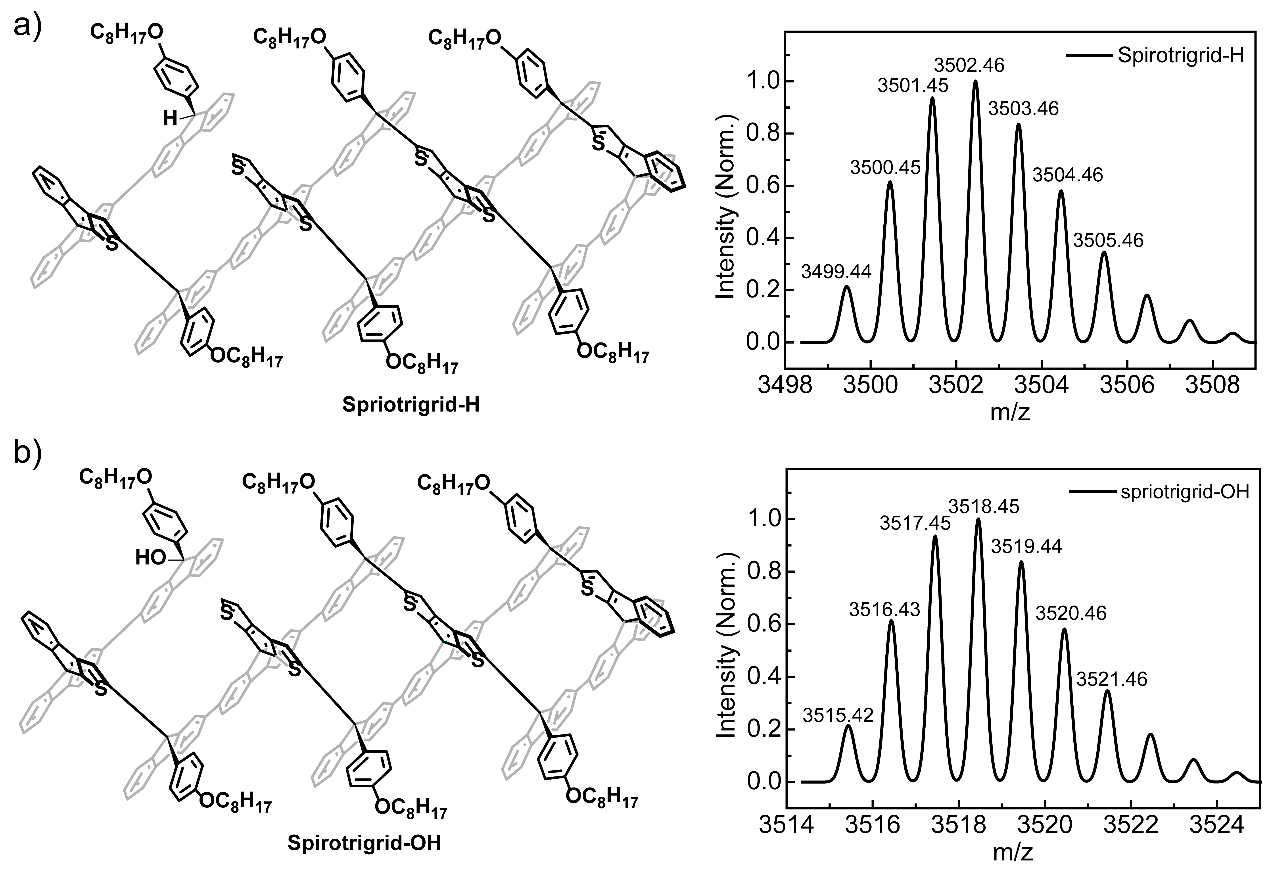


**Figure S31. MS spectra simulation of ungridized defective Spriotrigrid structures.** (a) dehydroxylated byproduct Spriotrigrid-H. (b) hydroxyl-containing byproduct spirotrigrid-OH.

The mass spectra of SDG (DP = 2) and spirotrigrid (DP = 3) are shown in Figure S29a and S29b, respectively, through the characterization of MALDI-ToF-MS. Their simulated isotopic distributions are also provided, as the standard. In Figure S29a, the mass spectra of SDG was measured as 2437.32, approximate to the theoretical results of 2437.00 (calculated from the formula of C_175_H_144_O_4_S_4_). Moreover, the measured isotopic distributions of SDG is also perfectly consistent with the theoretical simulation, confirming the SDG structures. It is noted that we also consider their corresponding products such as the ungridized products (SDG-H and SDG-OH, show in Figure S30a and S30b, respectively). However, the experimental mass spectra (m/z = 2437.32) is drastically different from SDG-H with m/z = 2439.02 (Figure S30a) and SDG-OH with m/z = 2455.00 (Figure S30b). Thus, the ungridized products are ruled out.

Although we cannot afford the exact mass spectra of oligomers with DP ≥ 4 based on the reflectance mode of MALDI-ToF. We luckily obtained the exact mass spectra of spirotrigrid (DP = 3), as shown in Figure S29b. The measured exact molecular mass of spirotrigird is 3497.92, approximate to the theoretical simulation of 3497.45 (although the higher measured molar mass unavoidably causes larger deviation, such deviation is also within the accepted scope of less than 0.8). Further, the characterized isotopic distributions of spirotrigrid is also perfectly in agreement with the theoretical calculation. In addition, such experimental results are also drastically distinct from ungridized defects such as spirotrigrid with m/z = 3499.44 (Figure S31a) and spirotrigrid-OH with m/z = 3515.42 (Figure S31b), which rules out the ungridized products. Thus, the molecular mass results supports the formation of NPSG, at least for their oligomers.


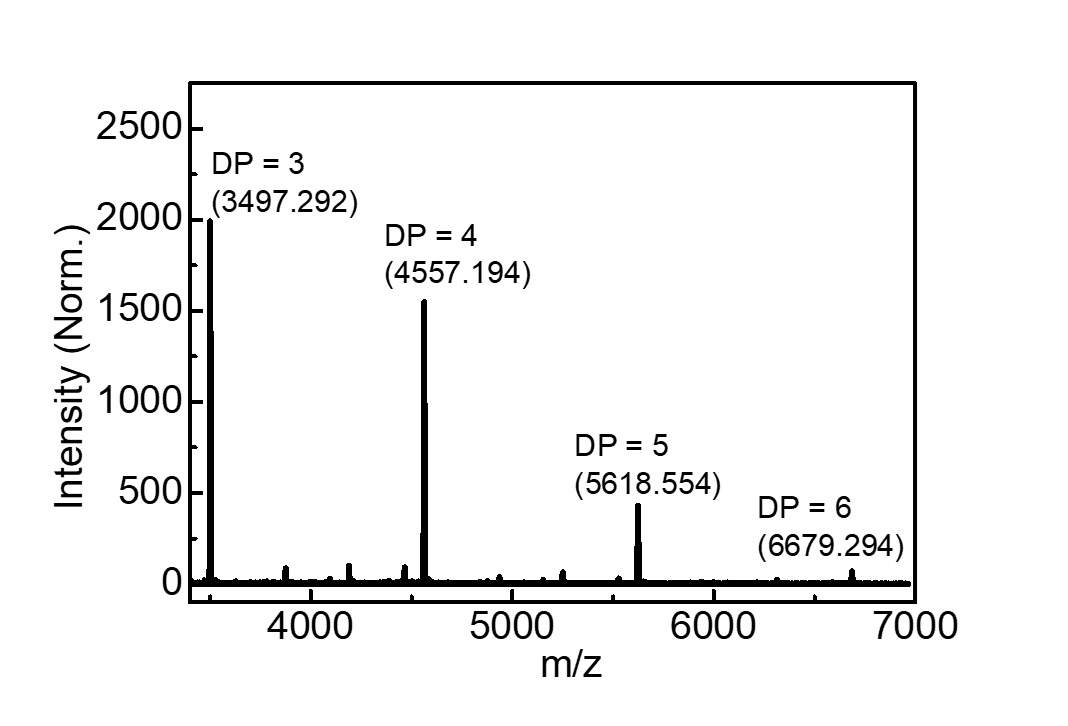


Figure S32. MS spectra of NPSG oligomers. The end-capping reagent ML (25% molar amount) were added after 1 min of polygridization reaction to ensure the low degree of polymerization DP = 3~7. We observed a series of ion peaks of NPSG oligomers: m/z = 3947.29 for DP = 3 (theoretical exact mass: 3947.43), m/z = 4557.19 for DP = 4 (theoretical exact mass: 4557.86), m/z = 5618.55 for DP = 5 (theoretical exact mass: 5618.30) and m/z = 6679 for DP = 6 (theoretical exact mass: 6678.73), respectively. Each molecular weight difference between adjacent DP values is about 1060, which is consist with the structural feature.

**Figure S33. ^13^C NMR spectra of STF-DOH, STF-DOH-2H (dehydroxylated STF-DOH, as the byproduct) SDG and NPSG.** The signal at 83 ppm in STF-DOH is assigned to the 9-position of fluorenols, where the electron-withdrawing effect of hydroxyl groups leads to a downshift by ~30 ppm (from 53~54 ppm on 9*H*-phenylfluorenyl groups [*11*]). When successful polygridization is performed, the hydroxyl groups are eliminated and the dehydrpxylation process does not occur. Correspondingly, the signals were shift to 63 ppm rather than 83 or 54 ppm. It is noted that the byproduct STF-DOH-2H was afforded by the addition of triethylsilane and BF_3_OEt_2_ to eliminate the hydroxyl groups.


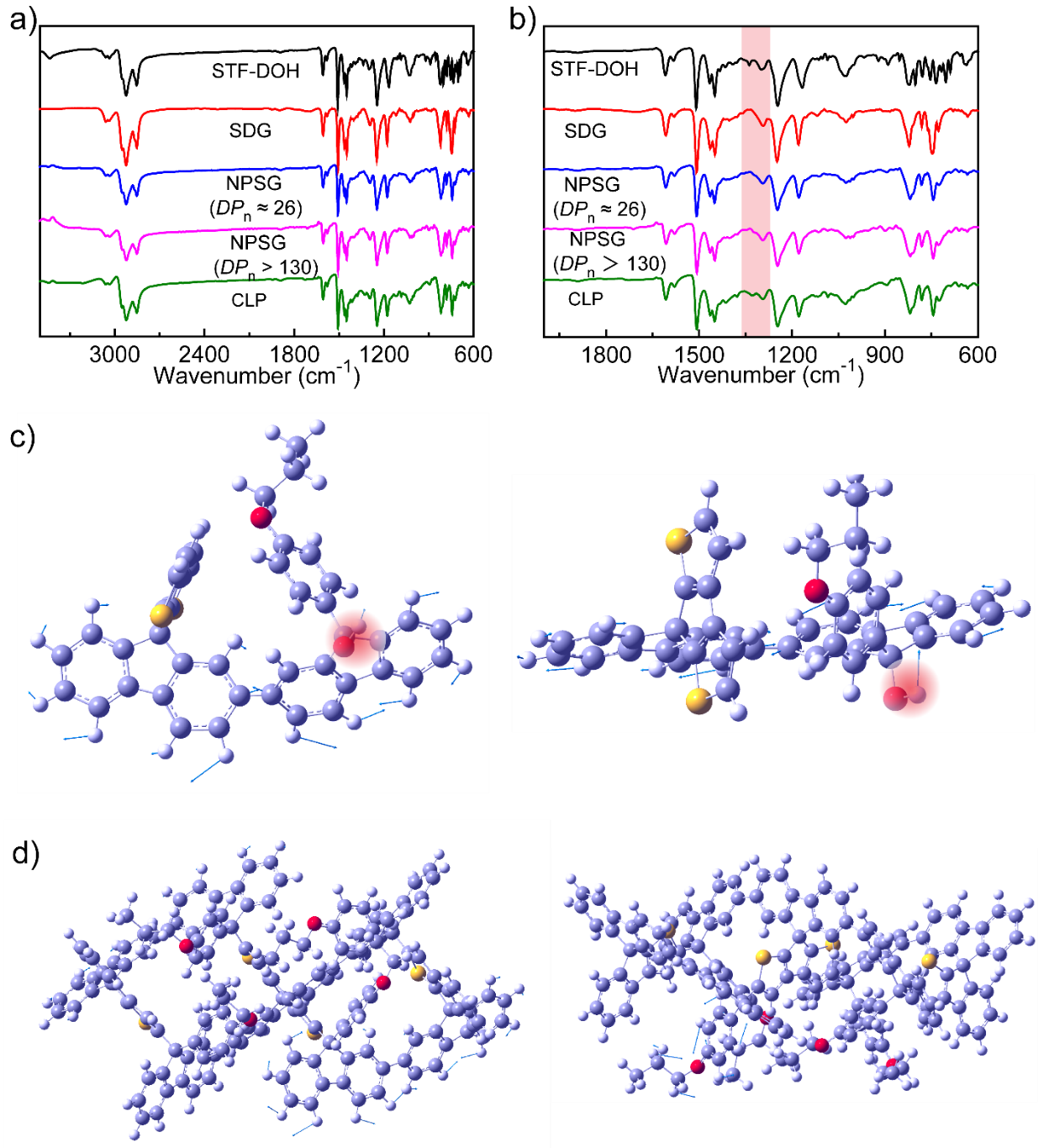


**Figure S34. FT-IR spectra of NPSG**. (a) Full spectra. (b) Partial spectra ranges from 2000 to 600 cm^-1^. The sample of NPSG (*DP*_n_ > 130) was derived from the spiro-polygridization under the STF-DOH concentration of 8 mM. According to its hydrodynamic radius (*R*_h_) of 70 nm on the hypothesis of maintaining linear configuration, we roughly evaluated *DP*_n_ > 130. The red region represents the difference between STF-DOH and NPSG, which probably belongs to the O-H scissoring vibration exhibited in the vibration simulation in (c). The blue lines are vibration trajectory of the vibrational motions. (d) The simulated vibration modes of SDG at 1335 cm^-1^. It is noted that the cross-linking polymer CLP possesses the weak IR absorption at 1340~1333 cm^-1^, which reveals the existence of hydroxyl groups on the chain-ends.

**7. Single-chain models and properties of NPSG**


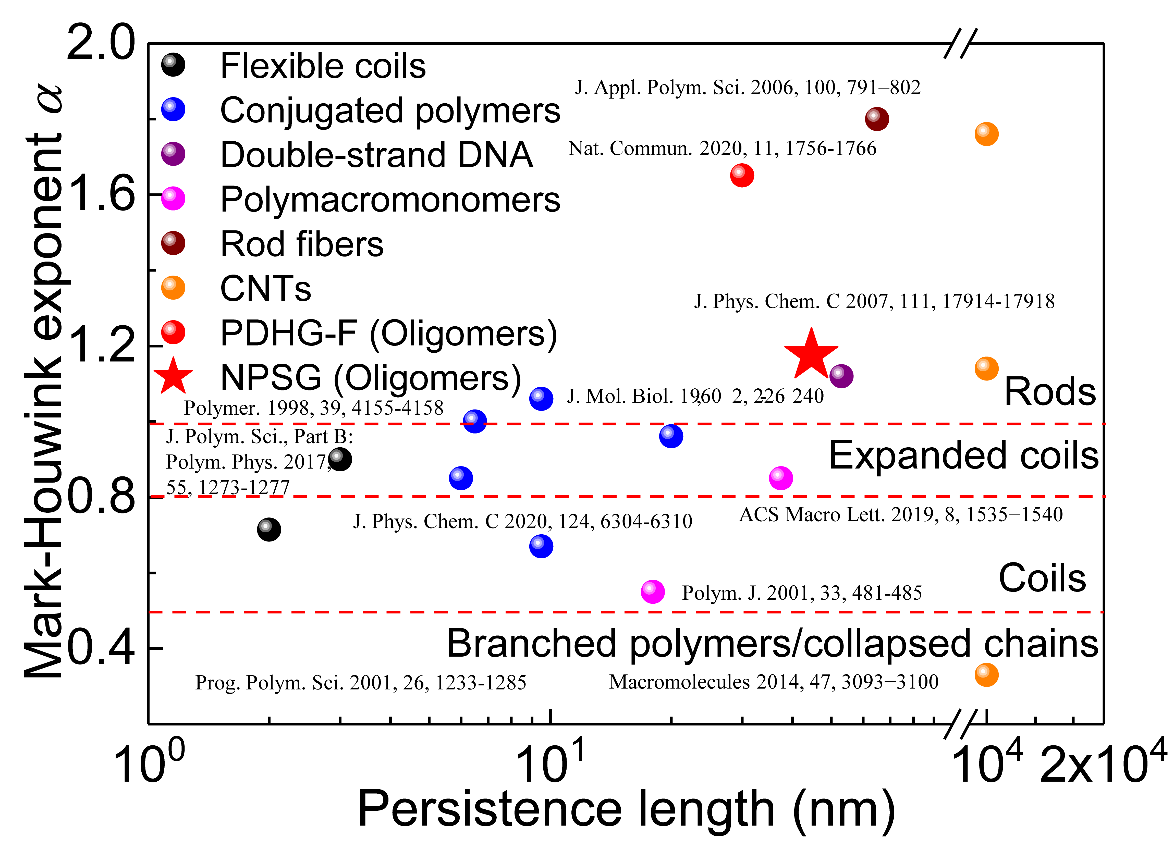


**Figure S35. The relationship between Mark-Houwink exponent *α* and persistence length (in *θ* or stretched state).** The NPSG is marked as a red star. These data were referred to the literatures [*3, 12-21*]. It is noted that the rod-like polymeric chains exhibit an Mark-Houwink *α* of 1.0~1.7; the coil-like chains exhibit an *α* of 0.5 (ideal coils in *Θ* state) ~ 0.8 (expaned coils in good solvent); the branched polymers can display an ultralow *α* < 0.5, because of the most structural compactness. As NPSG exhibits *α* = 1.175 that belongs to one-dimensional rod-like configuration, which is similar to double-stranded DNA. Further, Such *α* of NPSG is relatively lower than that of PDHG-F with *α* = 1.651, probably because of the more compactly structure.


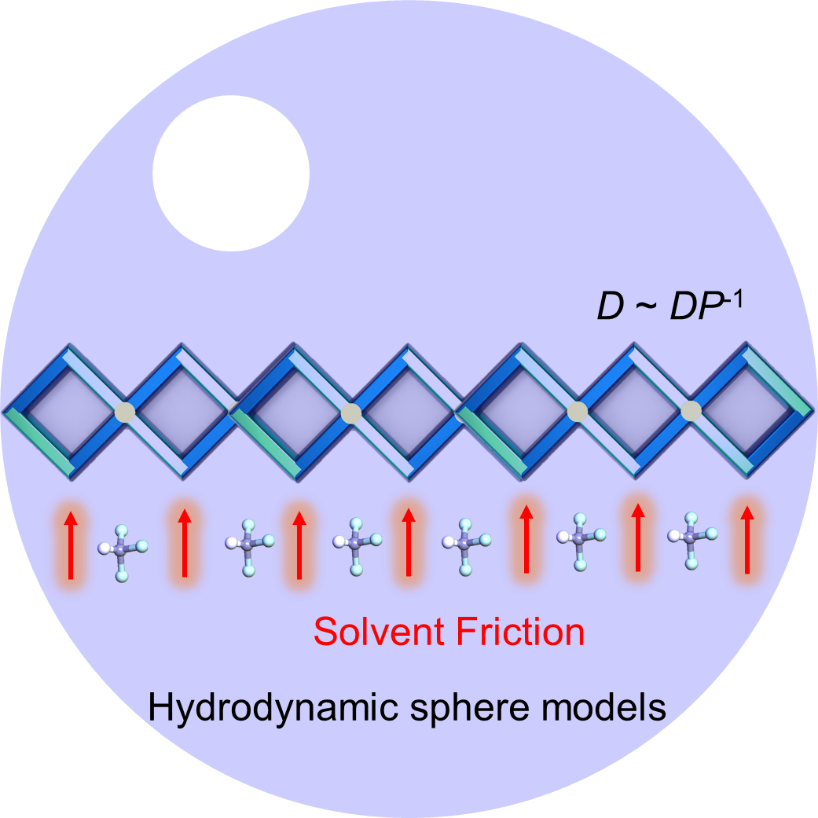


**Figure S36. The hydrodynamic radius models of the NPSG chain (*DP* = 8 as an example).** The blue sphere represents the hydrodynamic sphere model of the NPSG chain in which the main-chain acts like the diameter of the hydrodynamic sphere, as demonstrated by their hydrodynamic radius (*R*_h_) values that are approximately equal to the half of their contour length. The scaling laws of *R*_h_ ~ *DP*^1^ ~ *M*^1^ can be transformed into their diffusional coefficient laws as *D* ~ *DP*^-1^, based on the Stokes equation *D* = *kT*/6π*ηR*_h_, where *k* is the Boltzmann constant; *T* is the temperature and *η* is the solvent viscosity [*22*]. This model belongs to the free-draining type [*23*] in which each repeat unit RG suffers from the identical friction (marked in red arrows) from the CHCl_3_ solvent molecules (shown in atomistic models between red arrows). In contrast, in coil-like chains with partly non-free draining features (on repeat units), they exhibit the scaling law *R*_h_ ~ *M*^0.5^ for ideal chains and *R*_h_ ~ *M*^0.6^ for expanded coils. Therefore, the scaling laws *R*_h_ ~ *DP*^0.99^ of NPSG confirms the one-dimensional rod-like nanochains.


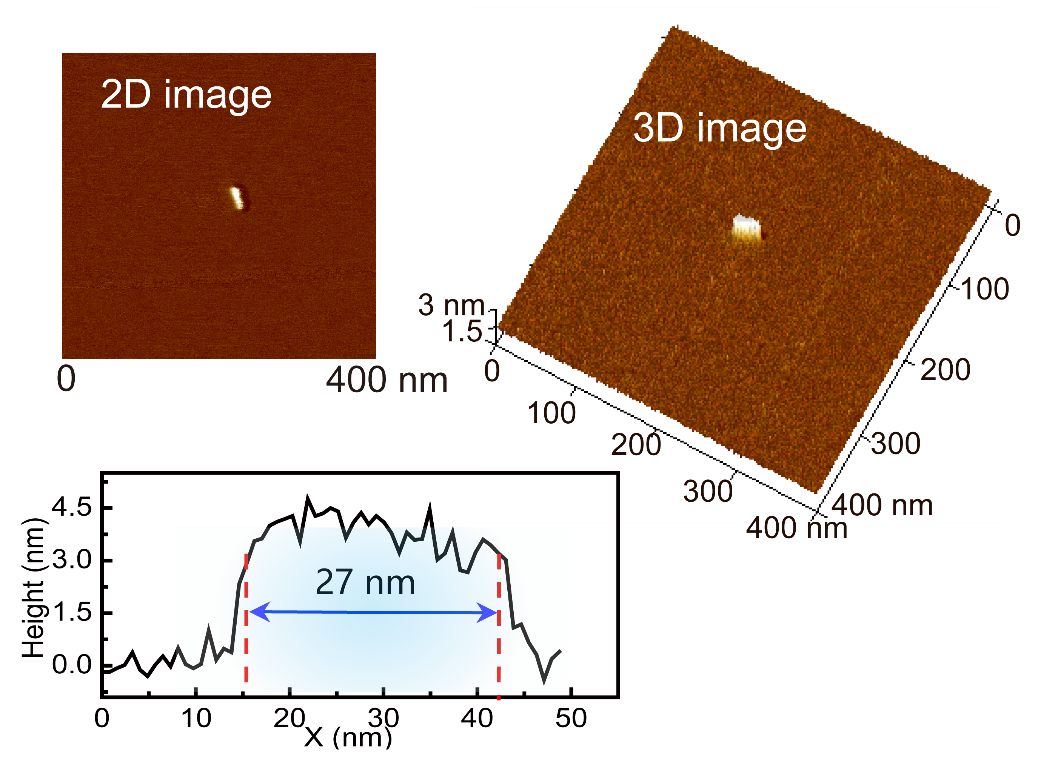


**Figure S37. The NPSG single-chain image from Atomic Force Microscopy (AFM), with the height profiles (in blue region).**


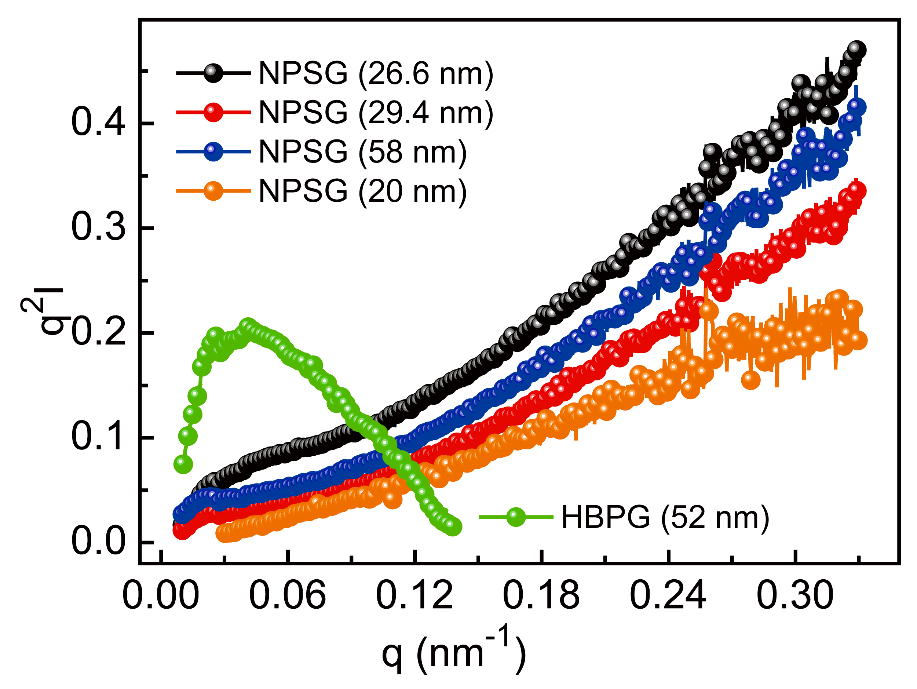


**Figure S38. The Kratky plots of NPSG (not green lines) and BSG (green lines, in the green region of branch with chain folding) in toluene.** The average contour length of NPSG (in the brackets) were transformed from individual *R*_h_ or *DP*_w_ values. Here, NPSG nanochains with various chain lengths exhibit the approximately linear relationships consistent with the rod-like chain modes [*24*], rather than the plateau that belongs to coil-like conformation [*25*]. Moreover, no arch pattern (with the maximum value) were observed on NPSG’s kratky plots, which firmly rules out the intrachain folding [*26*]. In contrast, the arch pattern with the peak is present in the branched byproduct HBPG, which indicates the existence of intrachain folding, consistent with the branching backbones [*27*].

**Analysis of the scattering law [*28*]**

The scattering law *I* ~ *q^-d^* provides the mass fractal dimension *d* that is related to the scaling laws *R* ~ *M*^1/^*^d^*, where *R* is the length of polymer chains and *M* can be defined as the molecular weight (or degree of polymerization) of polymers. For example, the random coil-like chains (in *Θ* solvent) exhibit *I* ~ *q*^-2^ and *R* ~ *M*^1/2^ from *d* = 2, which indicates the ideal two-dimensional conformations. Similarly, the expanded coils (in good solvent) display *I* ~ *q*^-5/3^ and *R* ~ *M*^0.6^ from *d* = 5/3, which suggests more structural anisotropy toward one-dimensional conformations. Analogously, the higher backbone anisotropy are shown in rigid rod-like chains (within a Kuhn length) with *I* ~ *q*^-1^ and *R* ~ *M*^1^ from *d* = 1 for ideal one-dimensional conformations. The collapsed polymer chains possess the compact spherical conformations with *I* ~ *q*^-3^ and *R* ~ *M*^1/3^ from *d* = 3 for ideal three-dimensional backbones, which covers the branched polymers as well


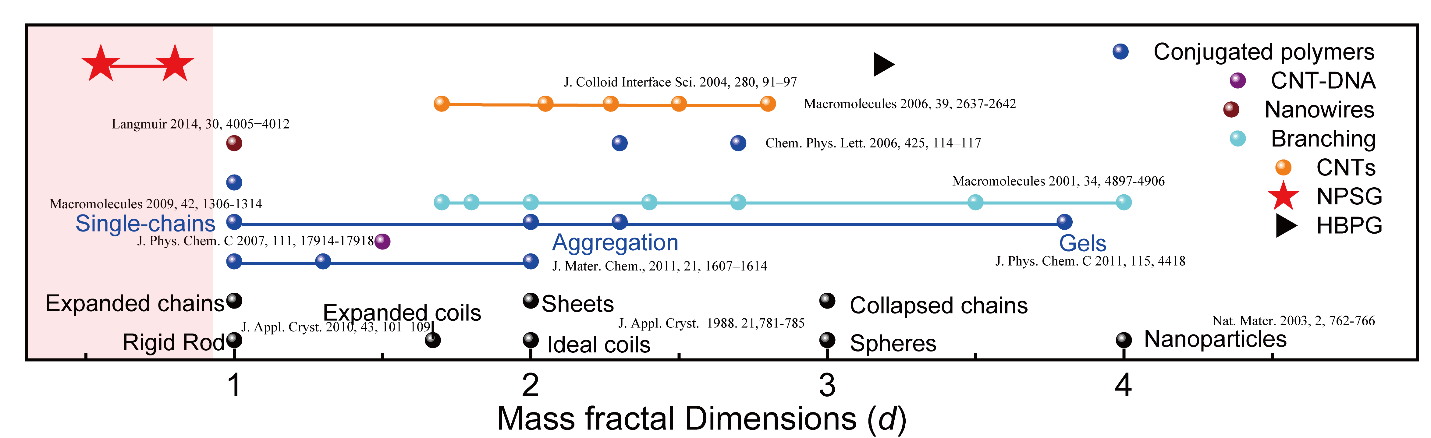


**Figure S39. The special mass fractal dimension of NPSG and other polymers.** The corresponding literatures are referred to the literatures [*17, 25, 27, 29-37*]. NPSG with various average chain lengths exhibit the scattering laws *I* ~ *q*^-0.55~-0.8^ that shows the ultralow mass fracture dimension approximate to rigid rod-like chains with ultrahigh structural anisotropy. In contrast, the branched byproducts HBPG exhibits the scaling law *I* ~ *q*^-3.18^ that reveals the high mass fracture dimension approximate to spherical conformation. These results significantly rules out the significant amount of branched byproducts in the NPSG samples.

The distance distribution function *P*(*r*) is related to the distribution probability of the distance that two repeat units are separated, which calculated from the indirect Fourier transform methods [*26*]:

$P\left( r \right)= \frac{r^{2}}{2\pi^{2}} \int_{0}^{\infty} q^{2}I\frac{\sin qr}{qr} dq$ ES3

For random coil-like chains or compact spherical conformations, their distance distribution functions *P*(*r*) exhibit the unimodal shape in which the maximum is distributed at the middle of *P*(*r*). Such maximum peak can be shifted to low *r*-region in expanded coil-like chains and rod-like polymers. The periodically nanoscale repeat units can even afford a series of peaks where the distance of two peaks is approximate to the distance of their monomers, like in polyporphyrins [*38*].


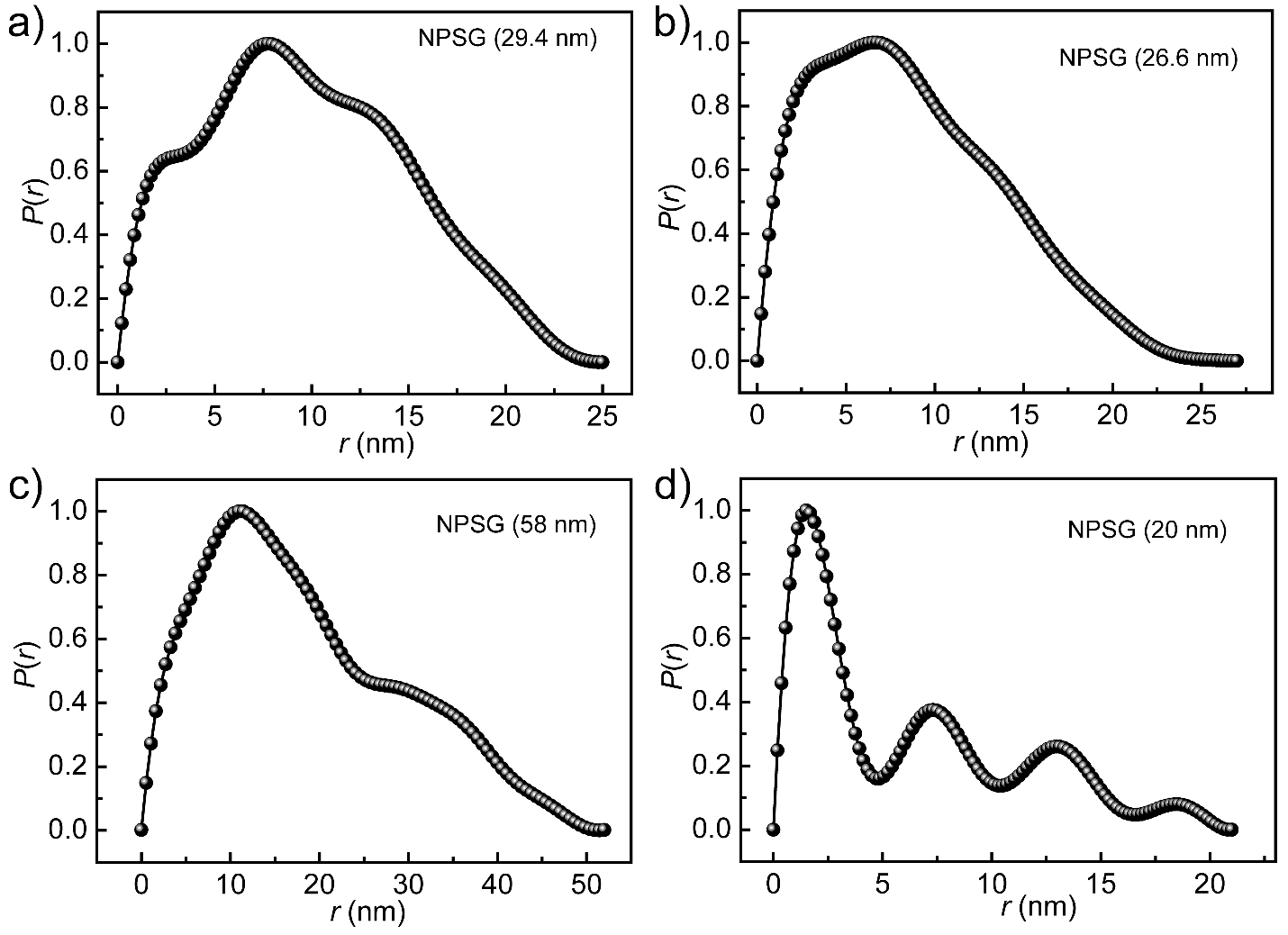


**Figure S40. The distance distribution function *P*(*r*) of NPSG with various average chain length.** The length marked in the blanks are the average value for NPSG chains, which are transformed from *R*_h_ values or their *DP*_w_ values.


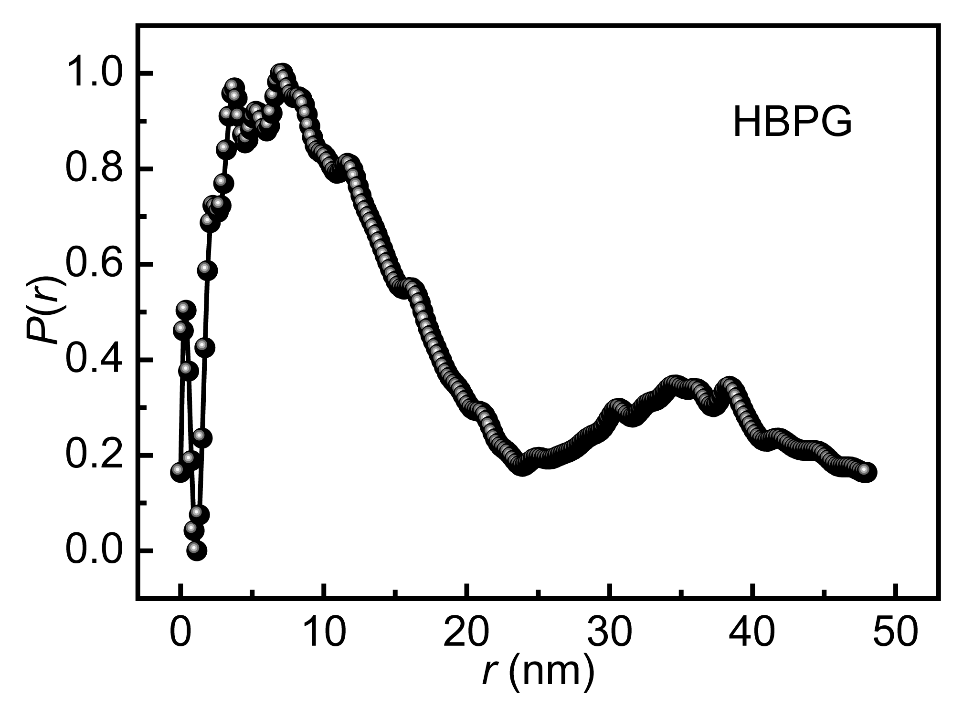


**Figure S41. The distance distribution function *P*(*r*) of HBPG**

**Radius of gyration (*R*_g_)**

The radius of gyration (*R*_g_) is the root-mean-square distance of the polymeric segments (or repeat units) from its center of mass on the main-chains. The *R*_g_ value is significant to evaluate the main-chain sizes and backbone flexibility (or rigidity) in which the higher *R*_g_ value suggests the stiffer main-chains if under the same contour length.

Using SLS characterization [*39*], *R*_g_ values can be obtained from the equation:

$\frac{Kc}{R_{\theta}}= \frac{1}{M_{w}} (1+ \frac{q^{2}R_{g}^{2}}{3}+2A_{2}M_{w}c)$ ES4

where *R_θ_* is the Rayleigh ratio that indicates the excess absolute scattering with the angular dependence (determined by scattering angles); *c* is the solution concentration; *M*_w_ is the weight-average molecular weight that is absolutely provided by the ALV/CGS-3 instrument rather than the calibrated value; *q* is defined as the scattering vector that is decided by the specific scattering angles. *A*_2_ is defined as the second virial coefficient. *K* is the constant that is related to the scattering light wavelength and the refractive index of polymer solution. During the measurements, we obtained a series of *K*c/*R*_θ_ values that corresponds to specific *q* values. The linear fitting of *K*c/*R*_θ_ ~ *q*^2^ affords their slopes *R*_g_^2^/(3*M*_w_), which then calculated the *R*_g_ values.

The other method to calculate *R*_g_ values is based on the Guinier laws [*40*] via the SAXS characterizations:

$I=I_{0}\exp(\frac{-R_{g}^{2}q^{2}}{3})$ ES5

where *I* is the scattering intensity of polymers; *I*_0_ is the forward scattering intensity. According to equation ES5, the linear fitting of ln *I* ~ *q*^2^ obtains their slopes (-*R*_g_^2^/3) that affords the *R*_g_ values. It is noted that this method should be on the prerequisite *qR*_g_ < 1.3. However, in the NPSG system, the scope of 1.3 < *qR*_g_ < 1.7 still allows for good linear fitting. Thus, to give more sufficient datum to linear fitting, we guaranteed the limited scope *qR*_g_ < 1.7 and we do not excess the scope *qR*_g_ > 2 that fails to generate accurate *R*_g_ values. It is noted that we do not use the equation ES6:

$I=I_{0}\exp(\frac{-R_{g}^{2}q^{2}}{2})$ ES6

to calculate the cross-section radius of gyration because of the poor fitting (the coefficient of determination *R*^2^ < 0.90)


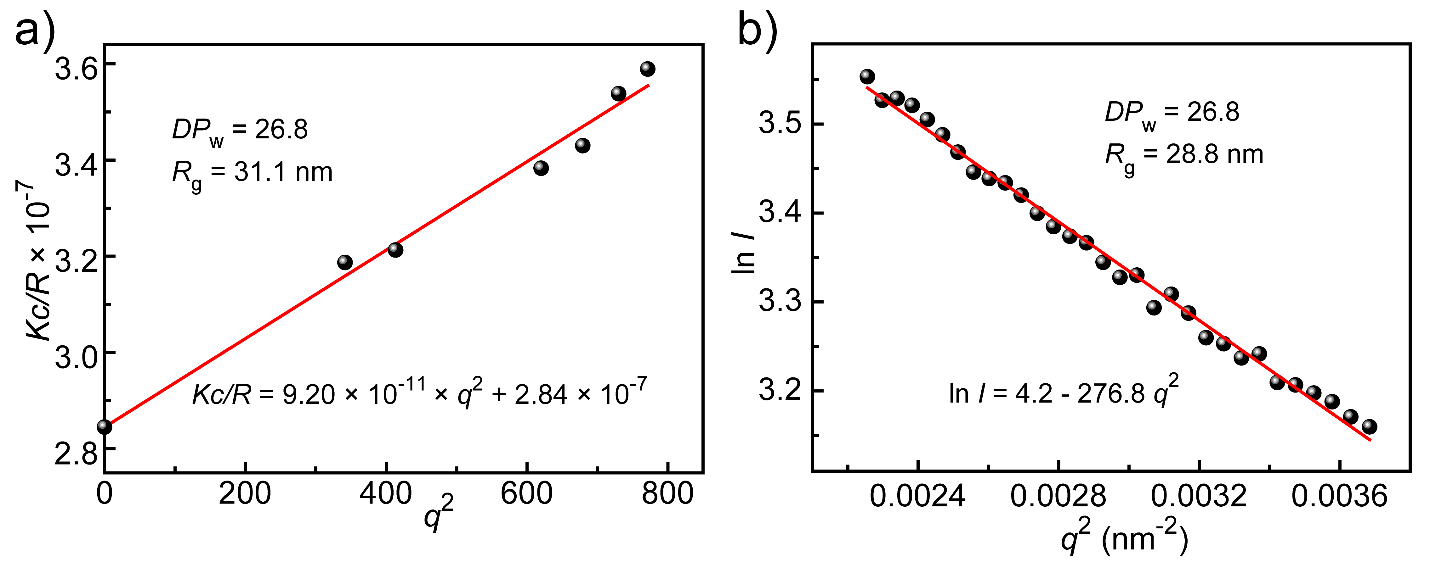


**Figure S42. The radius of gyration of NPSG with *DP*_w_ = 26.8.** (a) Calculated from the static light scattering. (b) Calculated from the Guinier laws via the SAXS characterizations.


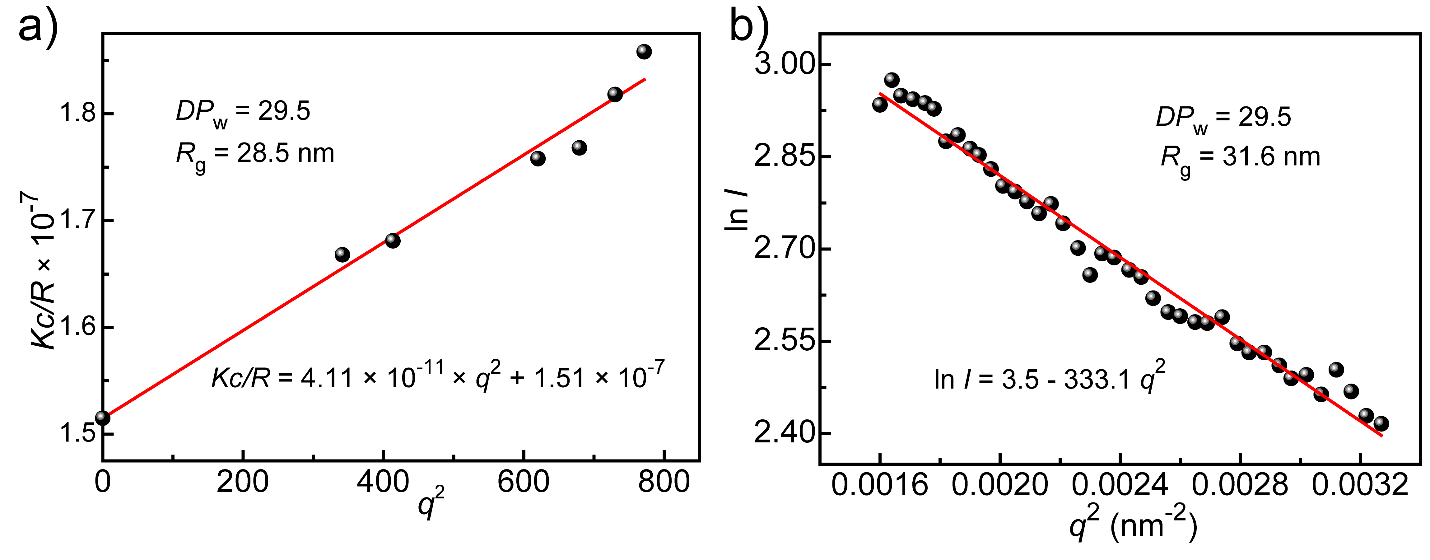


**Figure S43. The radius of gyration of NPSG with *DP*_w_ = 29.5.** (a) Calculated from the static light scattering. (b) Calculated from the Guinier laws via the SAXS characterizations.


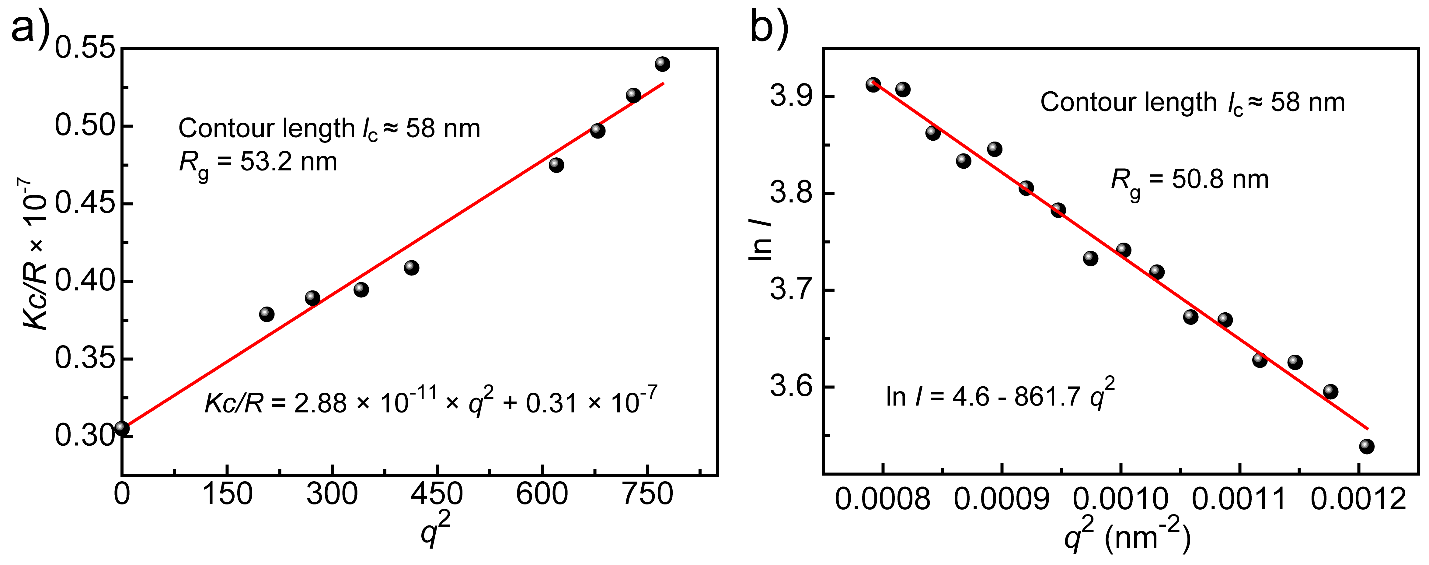


**Figure S44. The radius of gyration of NPSG with average contour length *l*_c_ ≈ 58 nm (roughly evaluated from their *R*_h_ values).** (a) Calculated from the static light scattering. (b) Calculated from the Guinier laws via the SAXS characterizations.


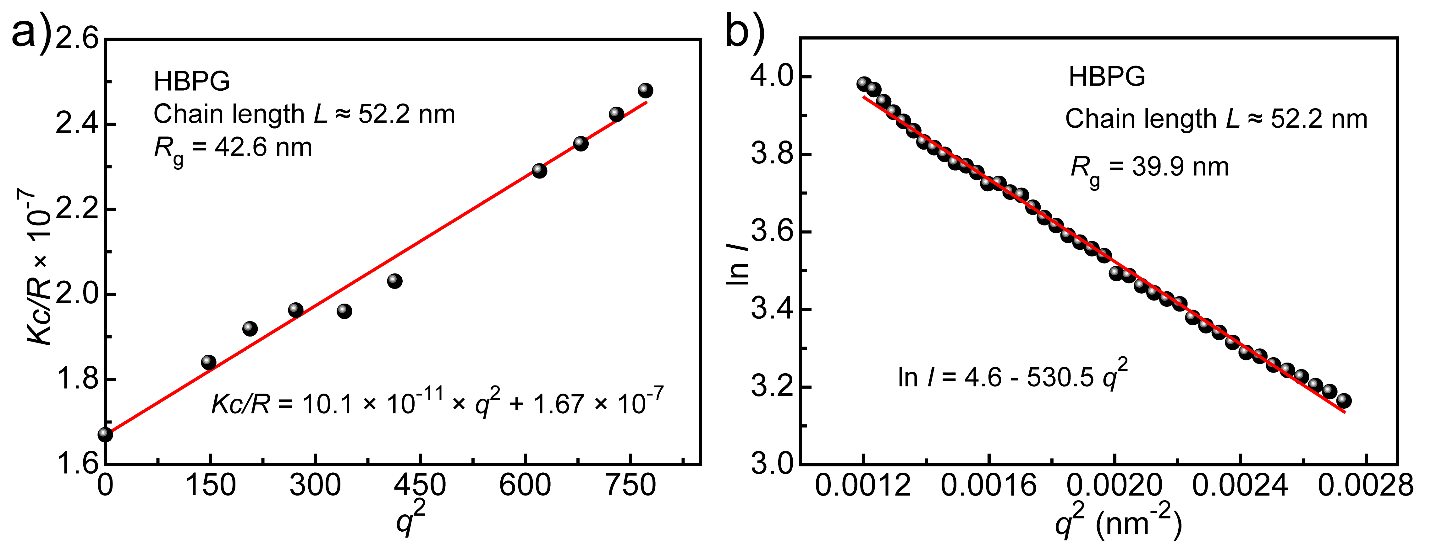


**Figure S45. The radius of gyration of HBPG with average chain length *L* ≈ 52.2 nm (transformed from *R*_h_ values).** (a) Calculated from the static light scattering. (b) Calculated from the Guinier’s laws via the SAXS characterizations.


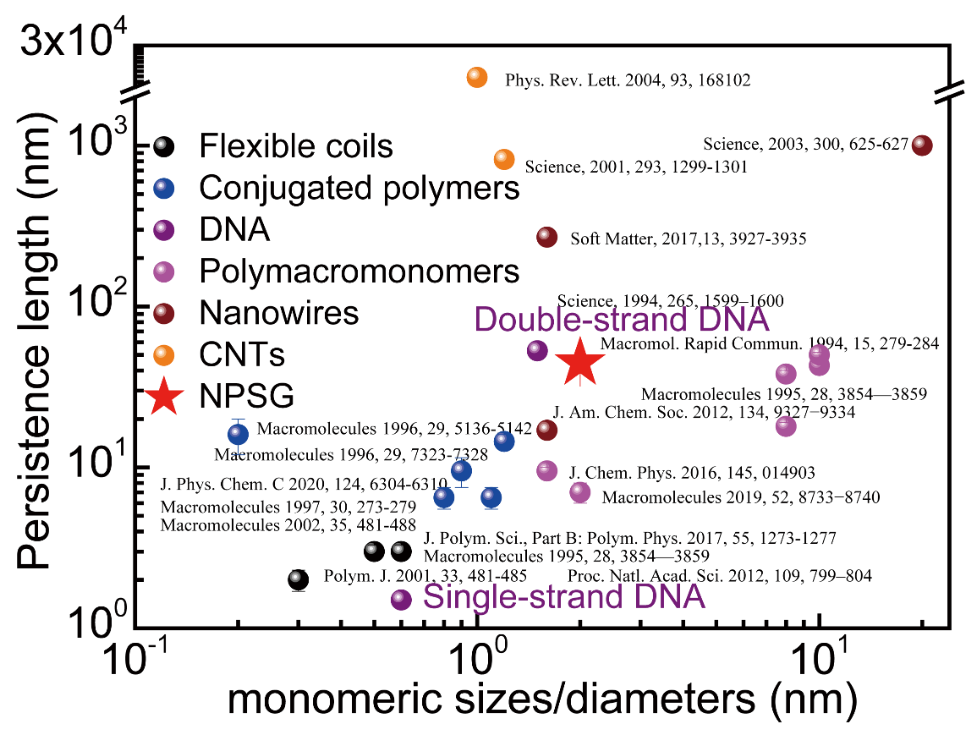


**Figure S46. The NPSG position (marked in red stars) in the statistical graph of persistence length and monomeric sizes.** The persistence length of other polymers (in *Θ* or good solvents) were referred to the literatures [*10, 14, 19, 21, 41-54*].

**8. Calculating the persistence length via molecular dynamic simulations**


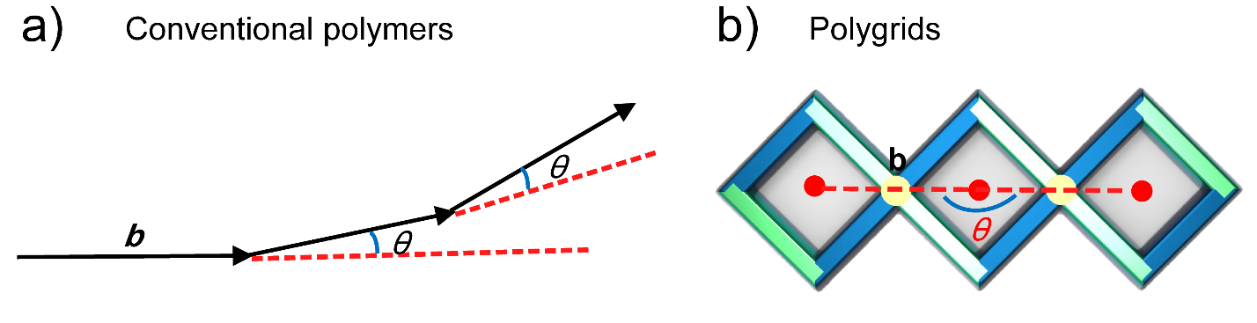


**Figure S47. The models of calculating persistence length.** (a) Conventional polymers. (b) Polygrids. The NPSG as the example, but the single-bond-linked polygrids (SBPGs) can be included as well. The *θ* is defined as the bending angle between two linkers, within the scope of 0~90^o^. As the polygrid model can provide the obtuse angle (marked as the 180^o^ - *θ*), we transform it into the complementary acute angle *θ*.

The persistence length value can be obtained from the inner product of segment vectors [*55, 56*] (shown in Figure S46):

$l_{p}= \sum_{i} b {cos}^{i} \theta$, *i* = 0, 1, 2, 3… ES7

In this equation, *b* is defined as the length of linked covalent bond and *θ* is the linked bond angles. For π-conjugated rigid polymers, *b* can be representative as the length of planar repeat units and <*θ*> is defined as the average bending angles between adjacent two repeat units. Considering its feature of geometric progression, we obtained:

$l_{p}=\frac{b (1 - {cos}^{n}<\theta>)}{1-\cos<\theta>}$ ES8

As 0 < cos *θ* <1, we considered:

$l_{p}=\frac{b}{1-\cos<\theta>}$ ES9

If <*θ*> is so small that we can use the approximation *cos* <*θ*> *=* 1 *–* <*θ*>*^2^/*2, equation ES9 can be transformed into equation ES10:

$l_{p}= \frac{2b}{<{\theta>}^{2}}$ ES10

However, the above processing of *b* and <*θ*> is not suitable for conjugation-interrupted polygrids that exhibit larger *θ* values and thus afford evidently lower *l*_p_. Herein, we construct such linked models for polygrids (trigrids, *DP* = 3 of polygrids, as the example, Figure S47b) in which three centroids of corresponding grid units afford the bending angle (for <*θ*> parts) and the distance of adjacent centroids (for *b* parts). Compared with other bending angle models, this construction effectively diminishes the influence of chiral diarylfluorene groups on the larger <*θ*> values. In this model (Figure S47b), as <*θ*> cannot be approximate to 0, we use the equation ES9 to calculate the *l*_p_ of NPSG chains.

We performed the molecular dynamic calculation of trigrids [including spirotrigrids and single-bond-linked rhombus-type trigrids (STRGs)] in vacuum that facilitates faster conformational relaxations and transitions in the picosecond scale. Nevertheless, the vacuum environment also supports the process of chain collapse [3], which sharply decreases the *l*_p_ values. Therefore, we only construct trigrids without alkyl chains to suppress chain collapse, for the simulation of conformational relaxations. In this work, the molecular dynamic simulation is performed under the NVT ensemble, the pcff forcefield and the time-step of 0.2 fs (total time of 100 ps, longer dynamic times also leads to chain collapse). Each conformational information (especially for the bending angle *θ*) was obtained at the interval of 0.2 ps.


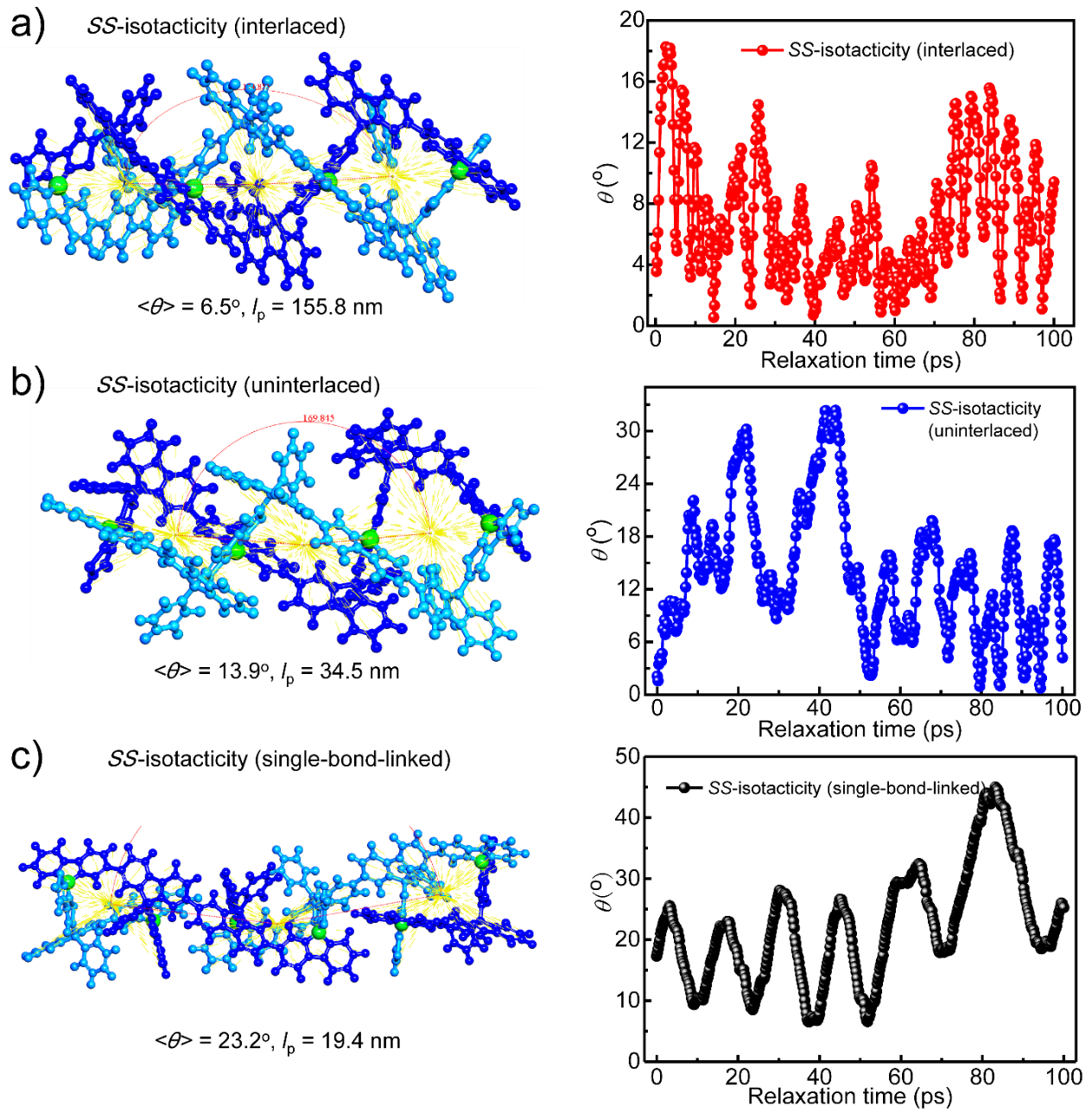


**Figure S48. The conformational relaxations of *SS*-isotactic spirotrigrids and single-bond-linked STRG.** (a) The *SS*-isotactic interlaced spirotrigrid with the bending angle *θ* during the dynamics (0~100 ps). (b) The dynamics of *SS*-isotactic uninterlaced spirotrigrid during the time scale of 0~100 ps. (c) The dynamics of *SS*-isotactic single-bond-linked STRG (0~100 ps). The average bending angles (<*θ*>, obtained from the dynamics 20~100 ps) and persistence length (*l*_p_) are provided as well.


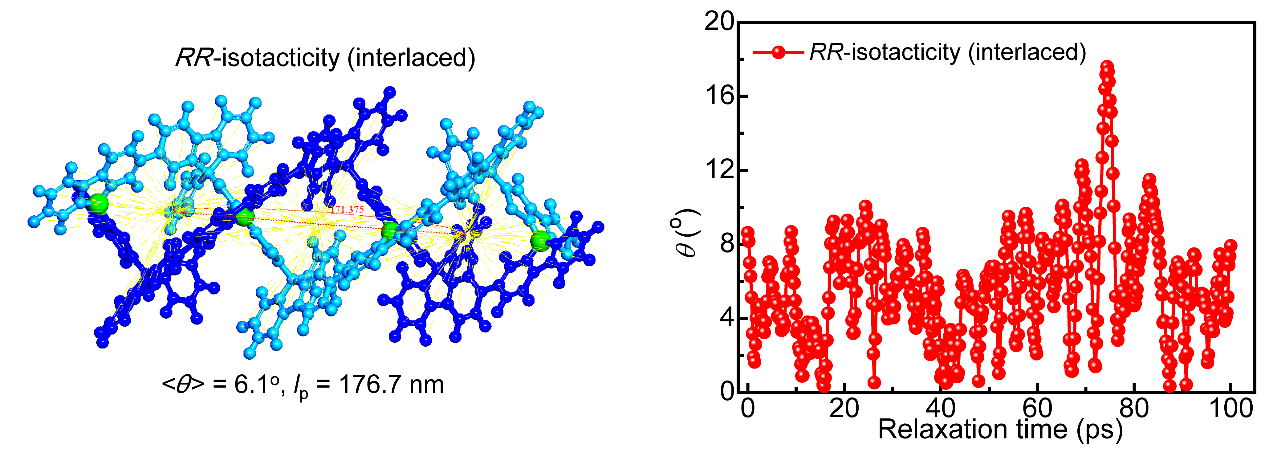


**Figure S49. The conformational relaxation of *RR*-isotactic spirotrigrid.** The average bending angle <*θ*> = 6.1° is approximately identical to *SS*-isotacticity, which indicates the almost same backbone rigidity.


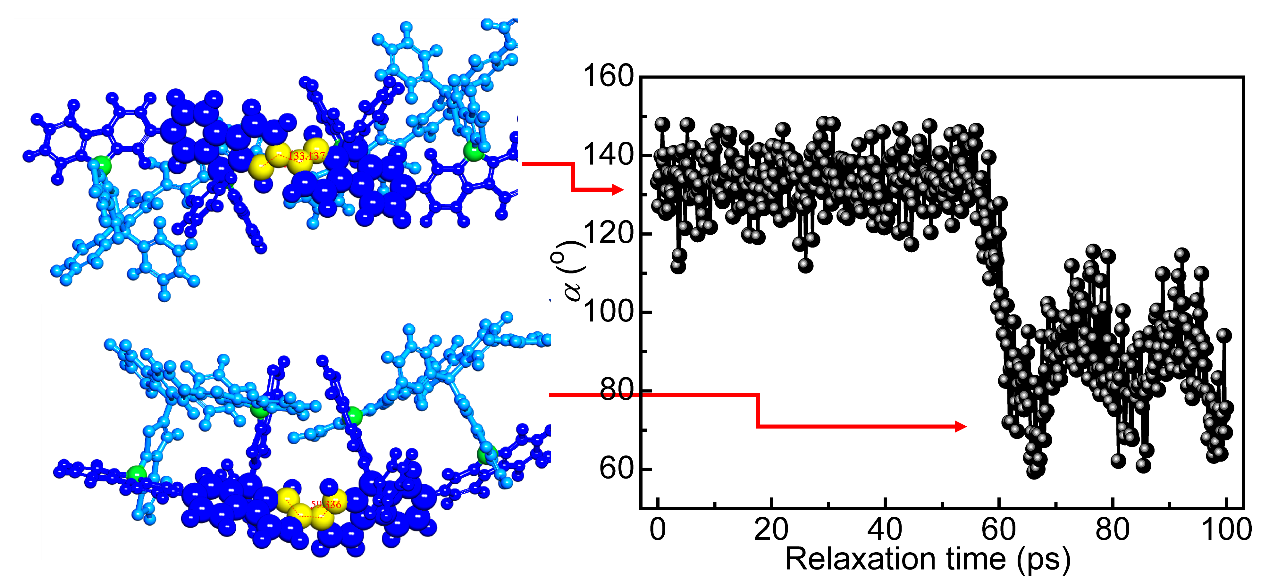


**Figure S50. The conformational transition of *SS*-isotactic single-bond-linked STRG.** The black dots represent the dihedral angle *α* between bifluorene moieties (marked as yellow spheres). The conformational transition from *anti* (*α* > 90°) to *syn* (*α* < 90°) occurs at about 60 ps, which is consistent with the beginning of the increase in bending motions.


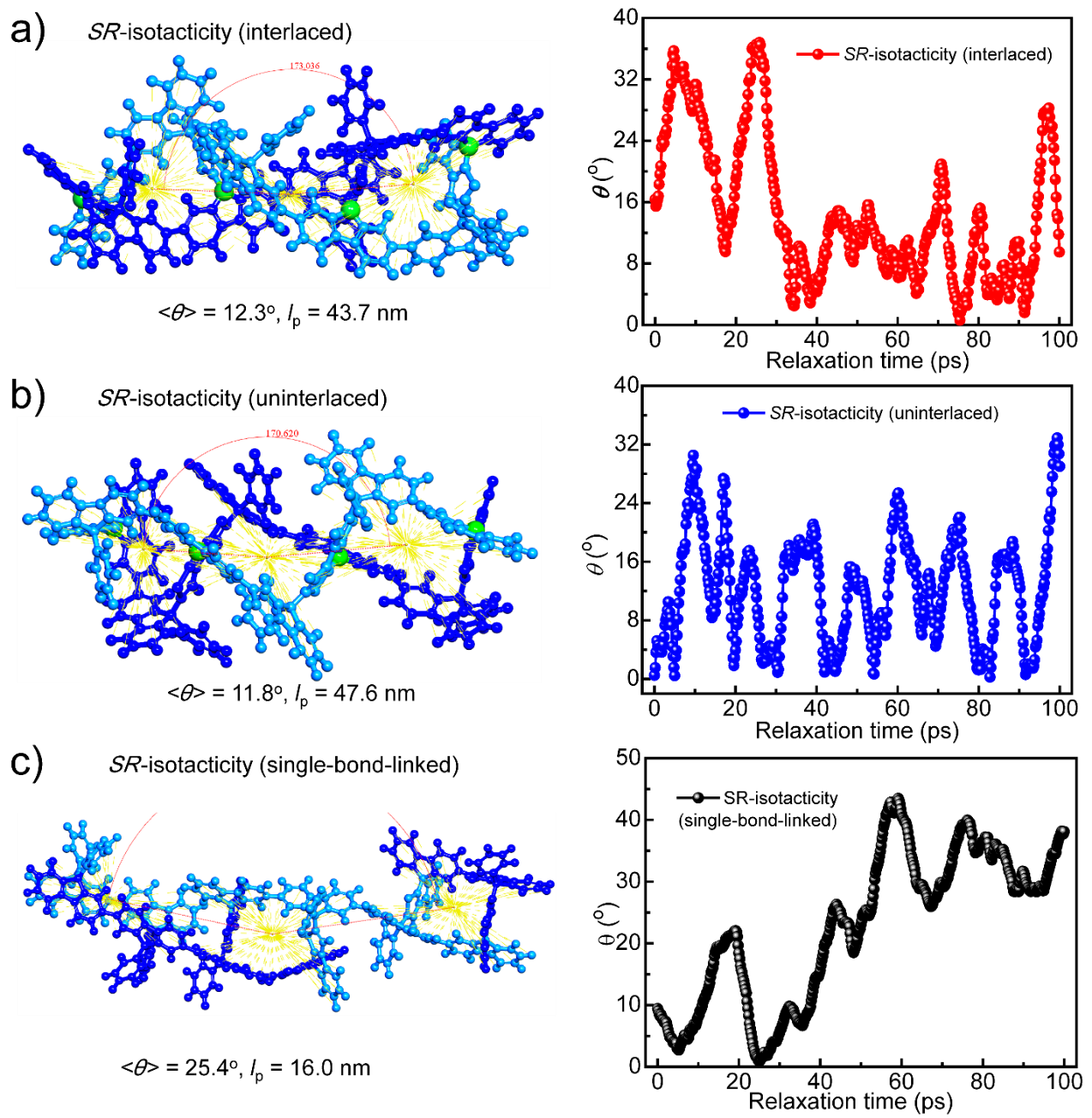


**Figure S51. The conformational relaxations of *SR*-isotactic spirotrigrids and single-bond-linked STRG.** (a) The *SR*-isotactic interlaced spirotrigrid with the bending angle *θ* during the dynamics (0~100 ps). (b) The dynamics of *SR*-isotactic uninterlaced spirotrigrid during the time scale of 0~100 ps. (c) The dynamics of *SR*-isotactic single-bond-linked STRG during the time scale of 0~100 ps. The <*θ*> (obtained from the dynamics 20~100 ps) and *l*_p_ are provided as well.


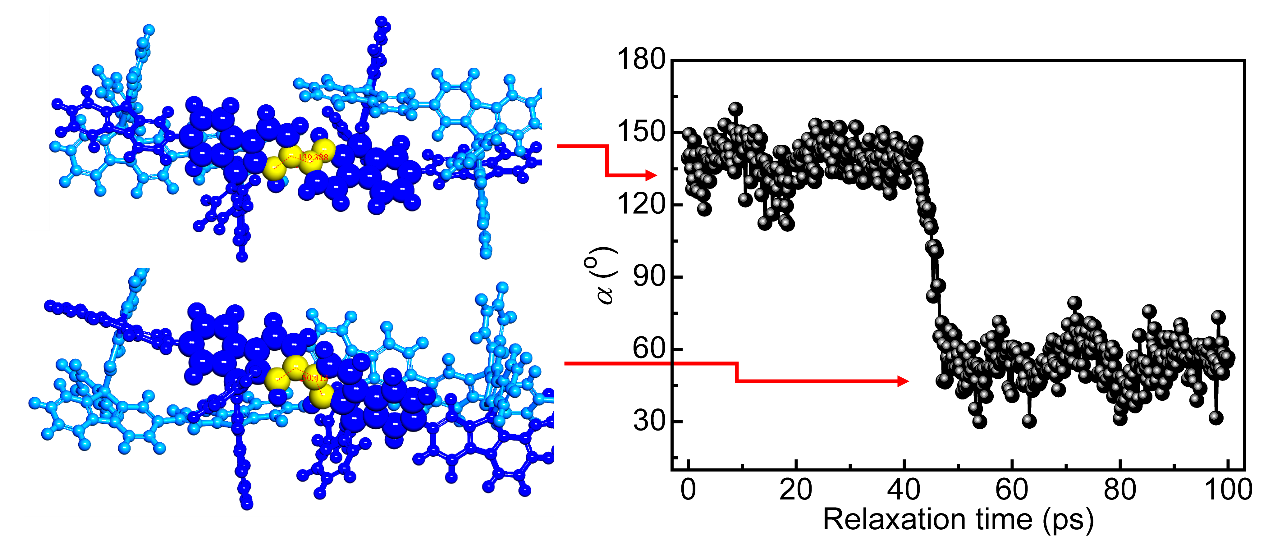


**Figure S52. The conformational transition of *SR*-isotactic single-bond-linked STRG.** The black dots represent the dihedral angle α between bifluorene moieties (marked as yellow spheres). The conformational transition from anti (*α* > 90°) to syn (*α* < 90°) occurs at about 50 ps, which is consistent with the beginning of the increase in bending motions.


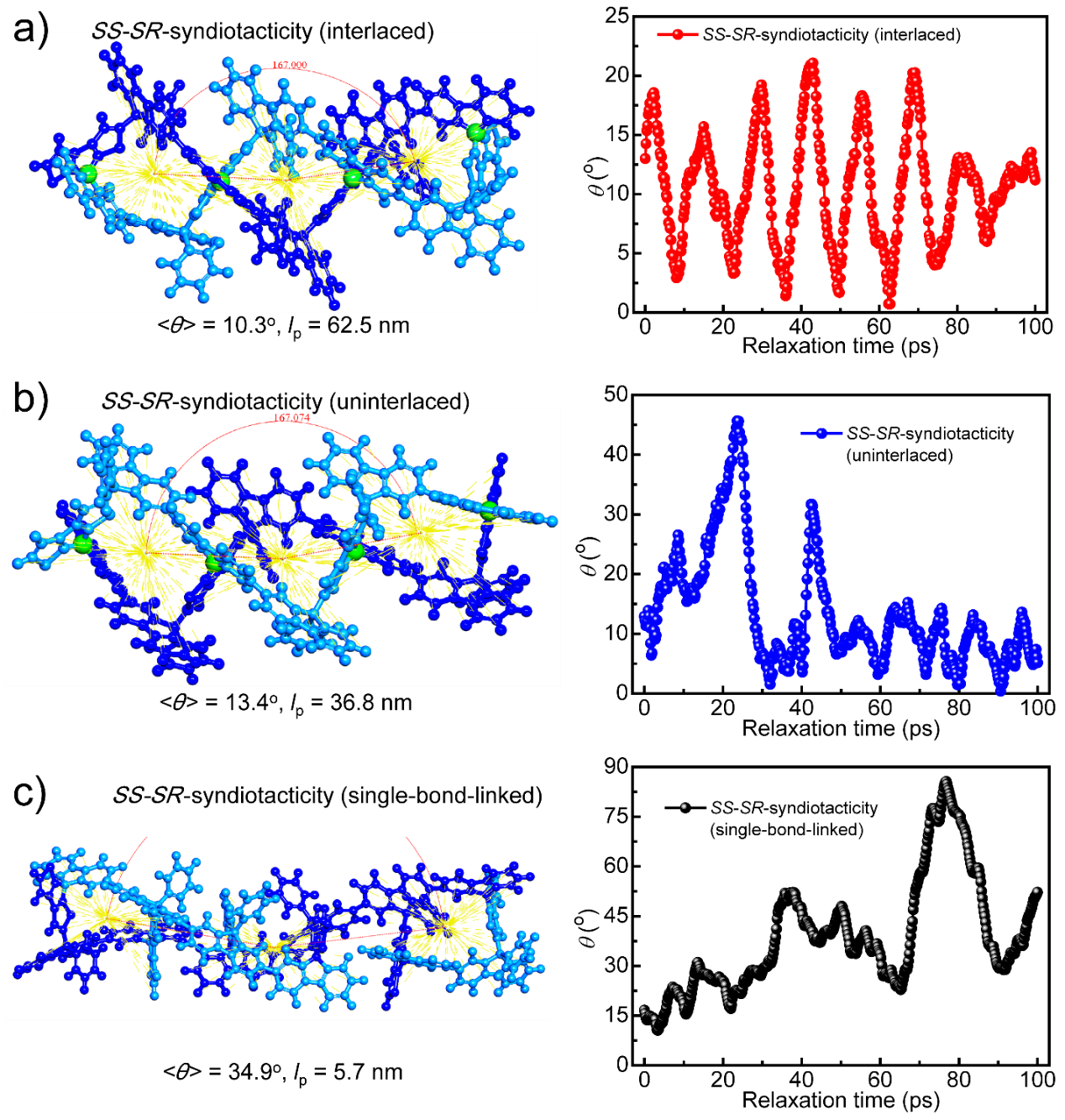


**Figure S53. The conformational relaxations of *SS*-*SR*-syndiotactic spirotrigrids and single-bond-linked STRG.** (a) The *SS-SR*-syndiotactic interlaced spirotrigrid with the bending angle *θ* during the dynamics (0~100 ps). (b) The dynamics of *SS-SR*-syndiotactic uninterlaced spirotrigrid during the time scale of 0~100 ps. (c) The dynamics of *SS-SR*-syndiotactic single-bond-linked STRG during the time scale of 0~100 ps. The <*θ*> (obtained from the dynamics 20~100 ps) and *l*_p_ are provided as well.


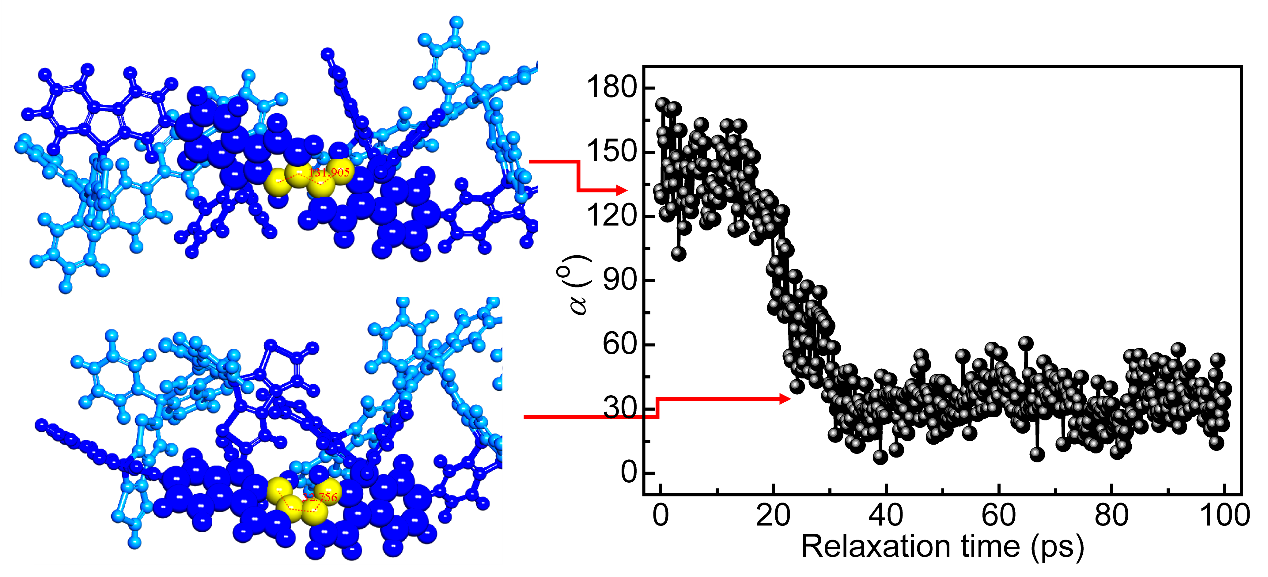


**Figure S54. The conformational transition of *SS-SR*-syndiotactic single-bond-linked STRG.** The black dots represent the dihedral angle α between bifluorene moieties (marked as yellow spheres). The conformational transition from anti (*α* > 90°) to syn (*α* < 90°) occurs at about 20 ps, which is consistent with the beginning of the increase in bending motions. As such transition (unequilibrium in the vacuum and toward chain collapse) is faster than that of *SS*-isotacticity and *SR*-isotacticity, this configuration exhibits evidently lower persistence length for the single-bond-linked SBPG.


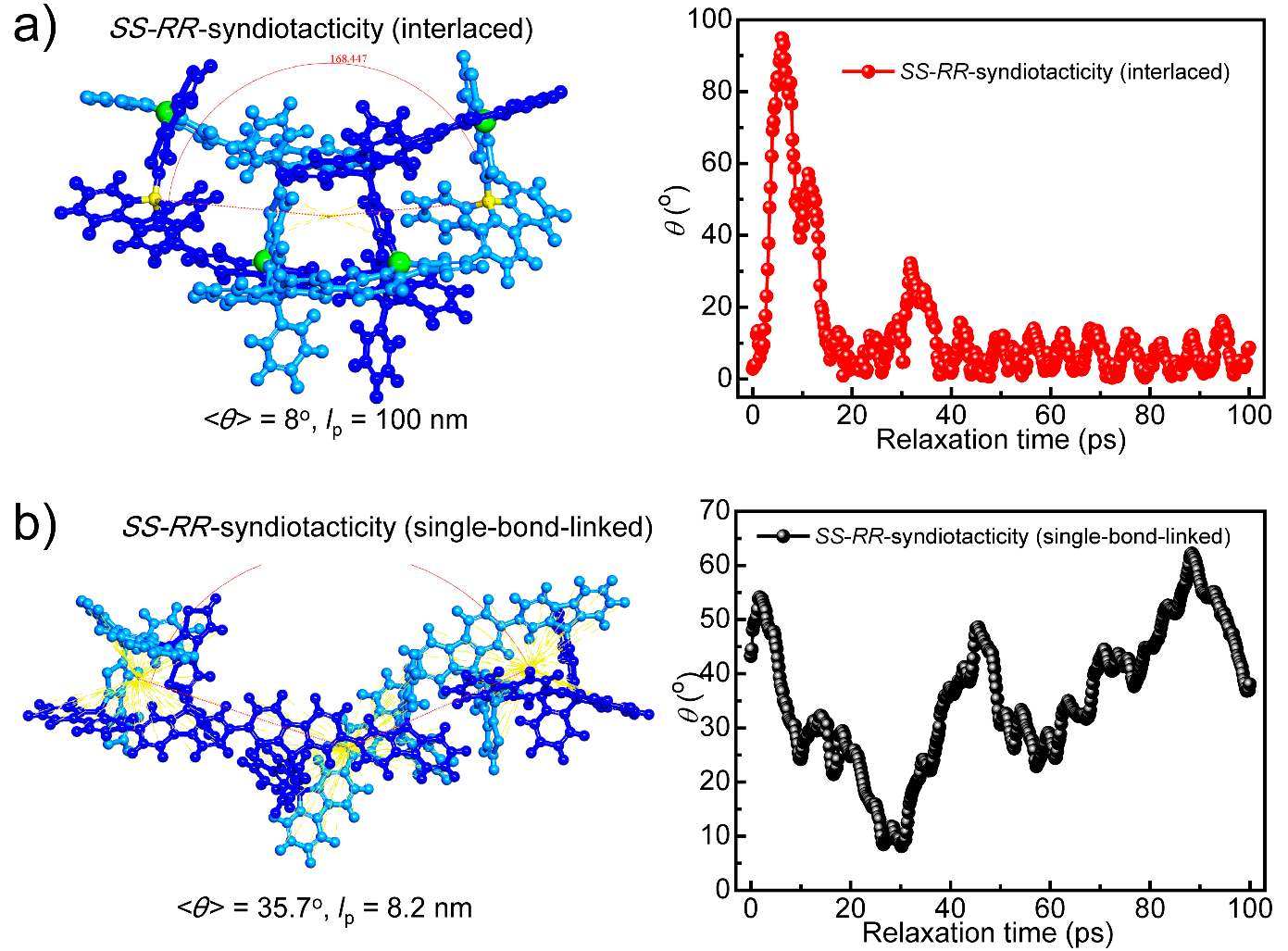


**Figure S55. The conformational relaxations of *SS-RR*-syndiotactic spirotrigrid and single-bond-linked STRG.** (a) The *SS-RR*-syndiotactic interlaced spirotrigrid with the bending angle *θ* during the dynamics (0~100 ps). (b) The dynamics of *SS-RR*-syndiotactic single-bond-linked STRG in the time scale of 0~100 ps. The <*θ*> (obtained from the dynamics 20~100 ps) and *l*_p_ are provided as well. The bending angles consist of two centroids of the RG units and one centroid between two dithiophene-planes.


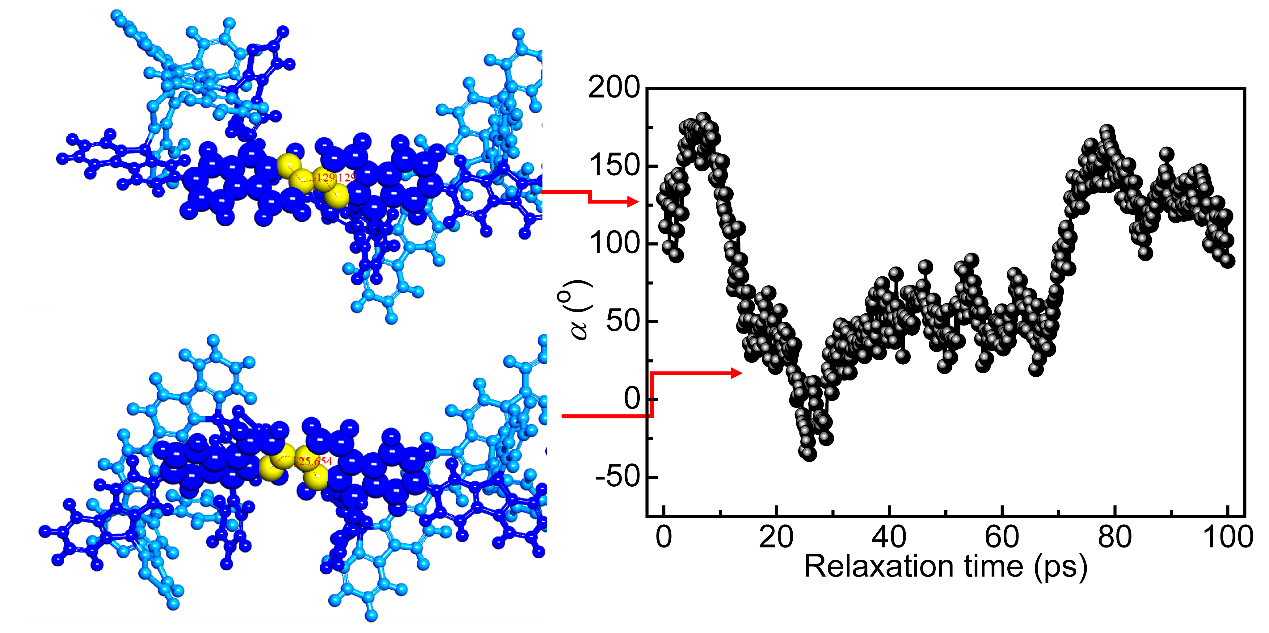


**Figure S56. The conformational transition of *SS-RR*-syndiotactic single-bond-linked STDG.** The black dots represent the dihedral angle *α* between bifluorene moieties (marked as yellow spheres). The conformational transition from *syn* (*α* < 90°) to *anti* (*α* > 90°) occurs at about 75 ps.


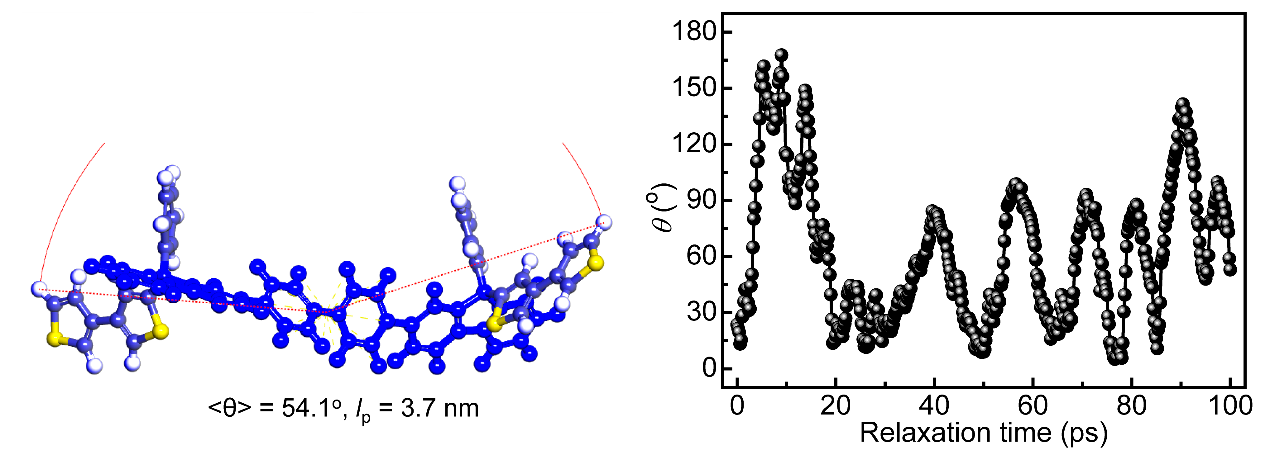


**Figure S57. The conformational relaxation of single-stranded ungridized oligomers whose polymers originates from the deletion of the spiro-carbon atoms on NPSG backbones (SSIP).** The bending angles consist of the thiophene-based chain-ends and the centroid of the biphenyl groups.

**Incorporation of ungridized defects**

The *l*_p_ vapue can also reflect the NPSG structure. The spiro-polygridized backbones with few ungridized defects should exhibit a long persistence length because of the conformational constraint from covalently double-bonded linkage. If the synthesized NPSG would have significant ungridized defects (as the other linear byproduct) with large amount, its persistence length should be much shorter in the scope of < 10 nm because of massive flexible single bonds on the main-chains [*45, 57*]. Inspired by this, we calculated the *l*_p_ value of defective NPSG backbones (with single-bond-linked moieties) through molecular dynamic simulation.


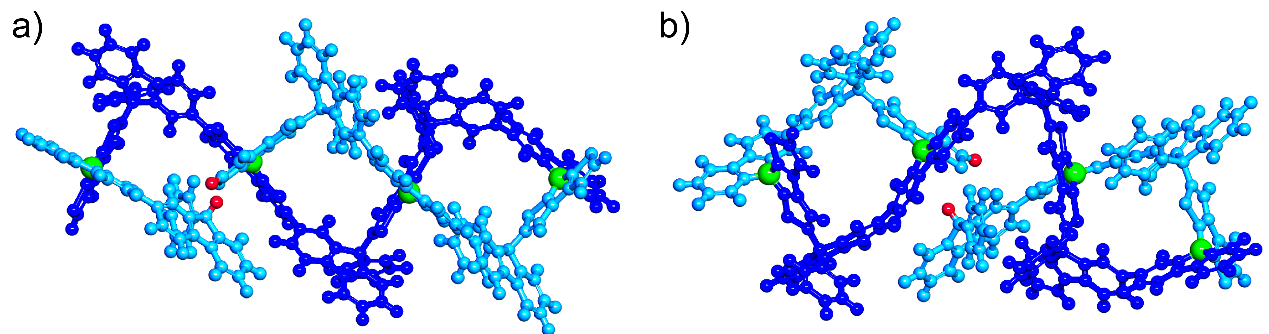


**Figure S58. The ungridized defective NPSG oligomer model.** (a) The ungridized defective moiety is set on the side nanogrid, which is denoted as the NPSG-DE1. (b) The ungridized defective moiety set on the middle nanogrids is denoted as the NPSG-DE2.


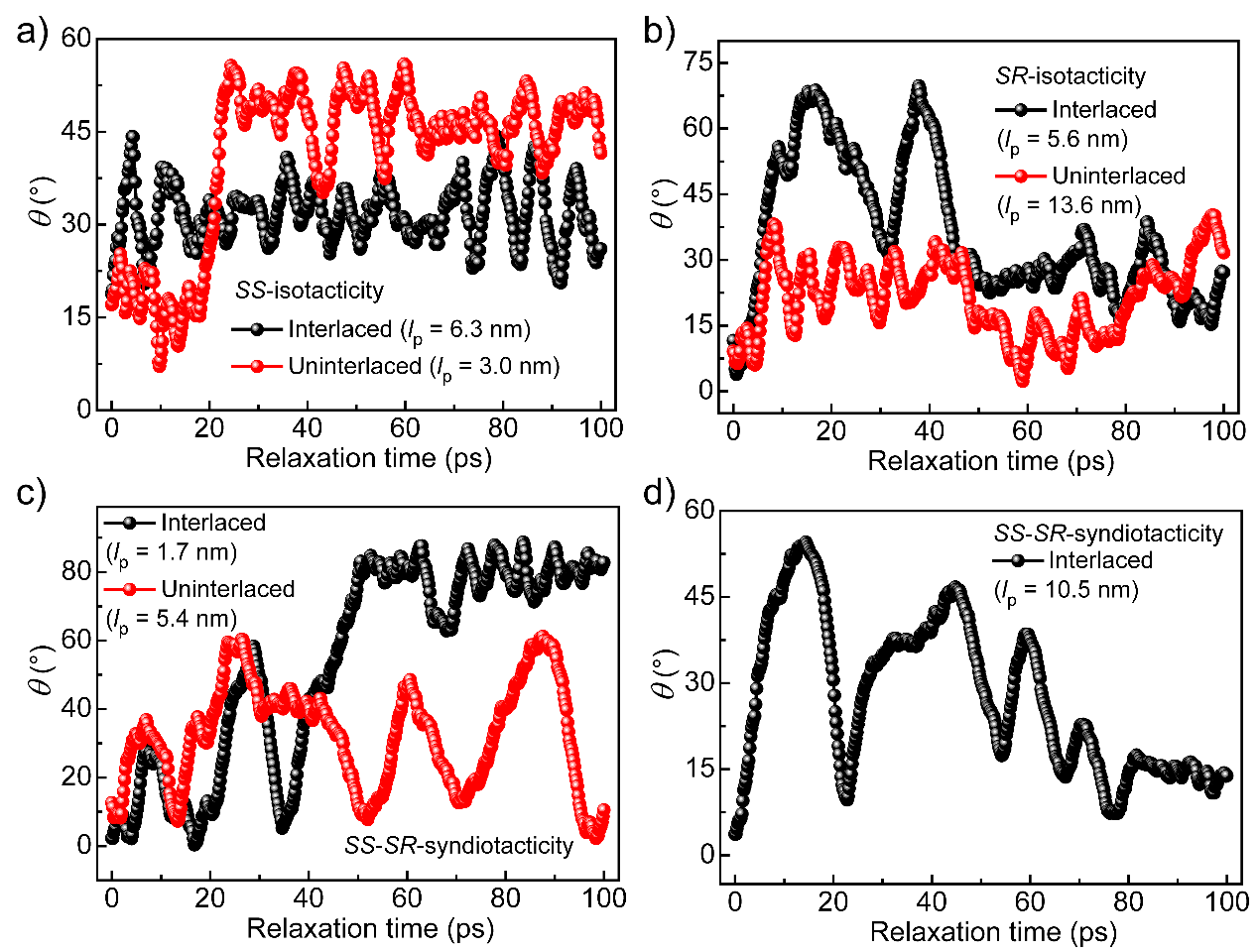


**Figure S59. The conformational relaxations of ungridized defective spirotrigrid (NPSG-DE1) with various tacticities.** (a) *SS-*isotacticity. (b) *SR-*isotacticity. (c) *SS-SR*-syndiotacticity. (d) *SS-RR*-syndiotacticity. All above tacticies also consider the interlaced (black dots and lines) and uninterlaced (red dots and lines) types. The *l*_p_ values are provided as well. We observed that the ungridized defective NPSG-DE1 structures possess much lower *l*_p_ values, including *l*_p_ = 6.3 nm for interlaced *SS*-isotacticity, *l*_p_ = 3.0 nm for uninterlaced *SS*-isotacticity, *l*_p_ = 5.6 nm for interlaced *SR*-isotacticity, *l*_p_ = 13.6 nm for uninterlaced *SR*-isotacticity, *l*_p_ = 1.7 nm for interlaced *SS*-*SR*-syndiotacticity, *l*_p_ = 5.4 nm for uninterlaced *SS*-*SR*-syndiotacticity and *l*_p_ = 10.5 nm for interlaced *SS*-*RR*-syndiotacticity, respectively. Thus, compared with NPSG nanochain chains without ungridized defects, the incorporation of ungridized defects sharply reduces the main-chain rigidity in 4~10 times.


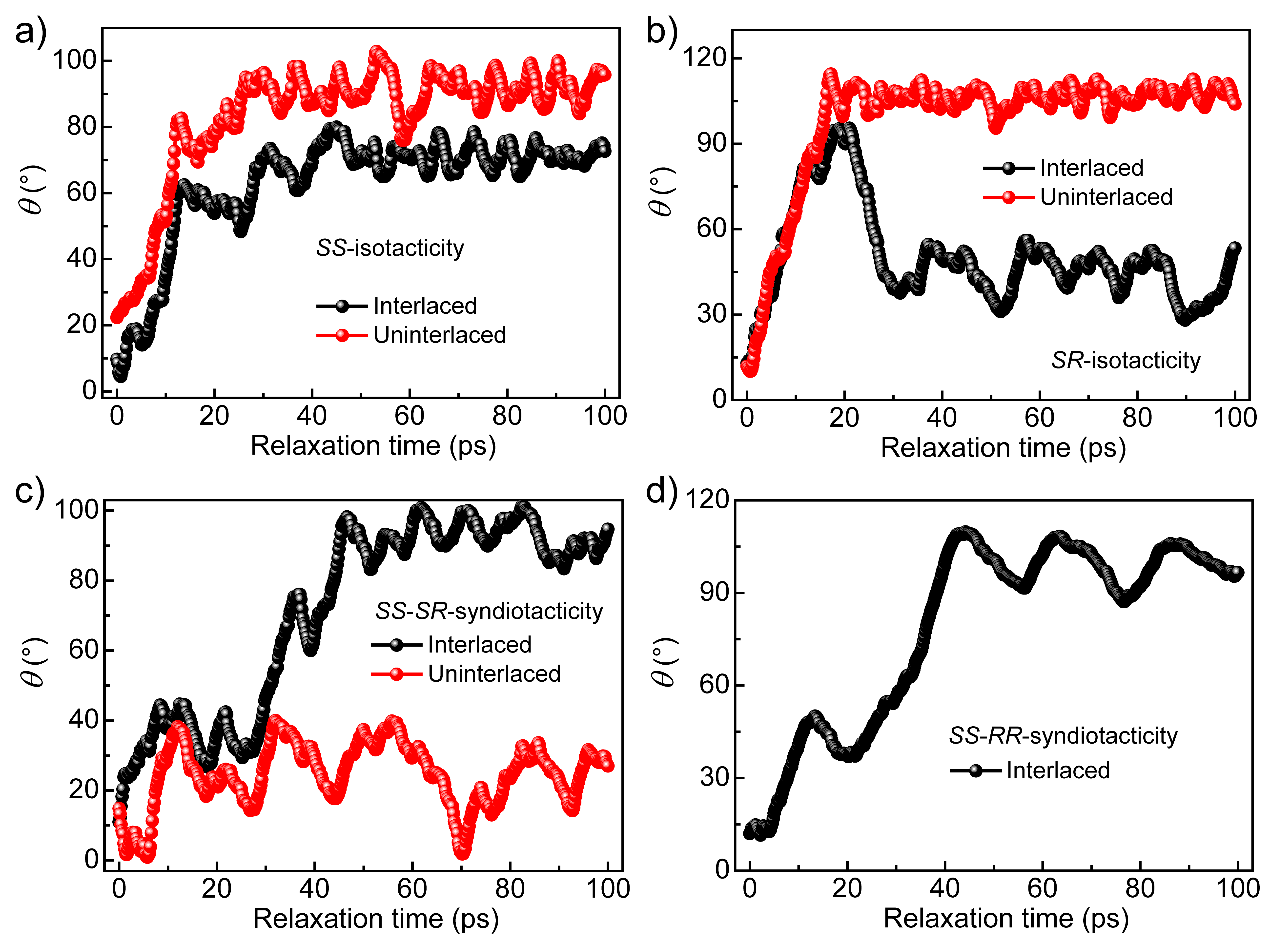


**Figure S60. The conformational relaxations of ungridized defective spirotrigrid (NPSG-DE2) with various tacticities.** (a) *SS-*isotacticity. (b) *SR-*isotacticity. (c) *SS-SR*-syndiotacticity. (d) *SS-RR*-syndiotacticity. All above tacticies also consider the interlaced (black dots and lines) and uninterlaced (red dots and lines) types. We observed that these NPSG-DE2 are much easier to perform chain collapse (with bending angle θ > 90°) that cannot afford *l*_p_ values. Even for partial uncollapsed cases, the *l*_p_ value is shorter than 10~15 nm. These results firmly confirm that the significantly defective NPSG should have flexible single-chains with short *l*_p_ values. Thus, our synthesized NPSG nanochains with an ultralong *l*_p_ = 41 nm should demonstrate the dominantly double-bond-linked backbones without significant ungridized defects on the main-chain.

**9. Dielectric properties**


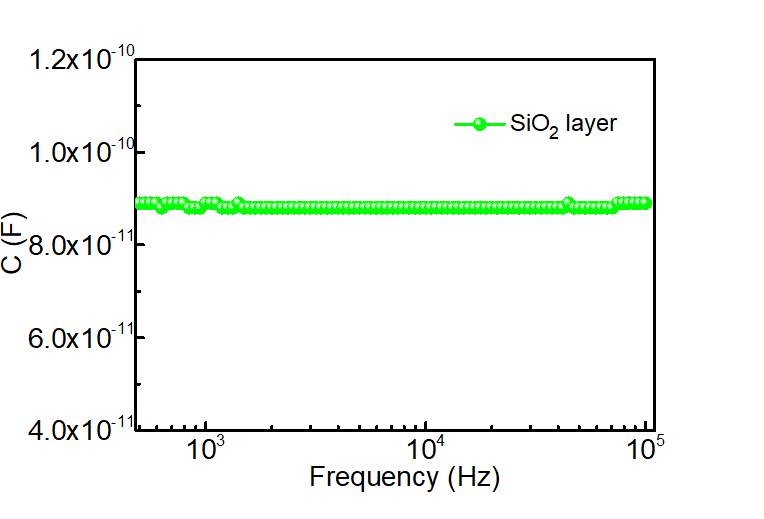


**Figure S61. The capacity of SiO_2_ layer.** For such device, the thickness of SiO_2_ oligomer film (*d*) was about 300 nm. The electrode area was 0.77 mm^2^.


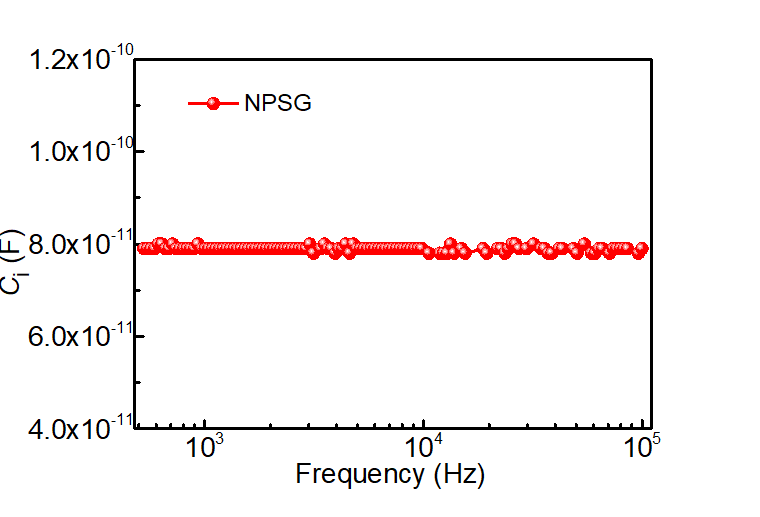


**Figure S62. The capacity of NPSG-based diode device.** The device fabrication process is similar to that of NPSG. For such device, the thickness of NPSG film (*d*) was about 73 nm; the thickness of SiO_2_ layer was 300 nm; the electrode area was 0.77 mm^2^.


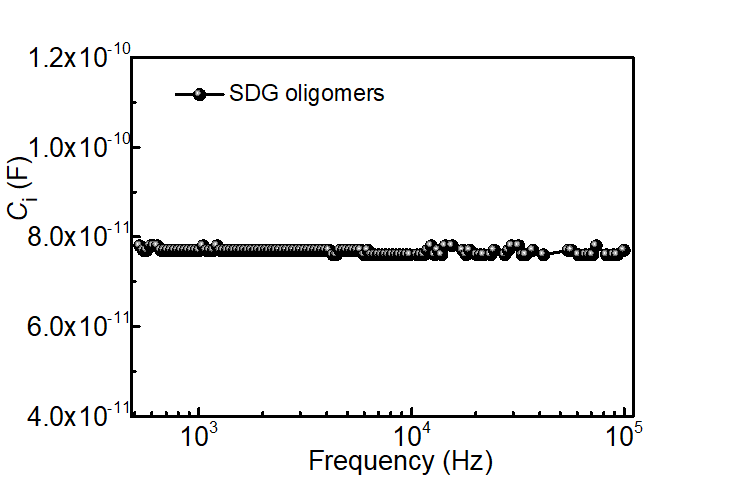


**Figure S63. The capacity of SDG oligomers-based diode device.** The device fabrication process is similar to that of SDG. For such device, the thickness of SDG oligomer film (*d*) was about 48 nm; the thickness of SiO_2_ layer was 300 nm; the electrode area was 0.77 mm^2^.


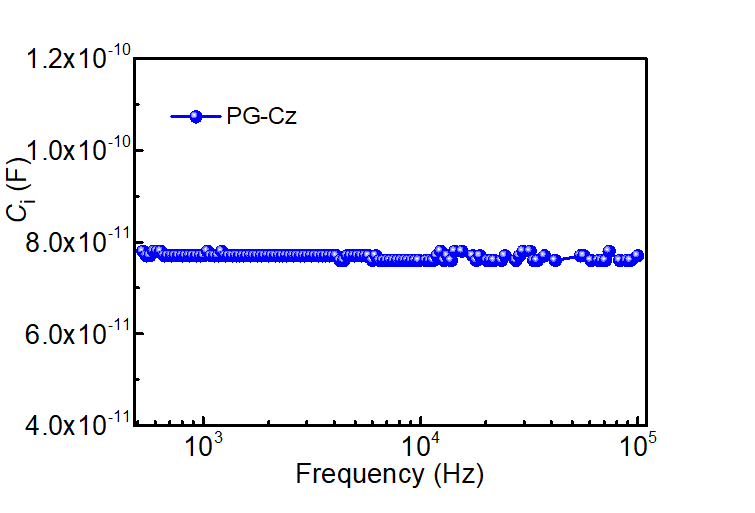


**Figure S64. The capacity of diode device with PG-Cz.** The synthesis of these single-bond-linked polygirds was referred to the literature [*58*]. The device fabrication process is similar to that of NPSG. For such device, the thickness of SDG oligomer film (*d*) was about 65 nm; the thickness of SiO_2_ layer was 300 nm; the electrode area was 0.77 mm^2^.


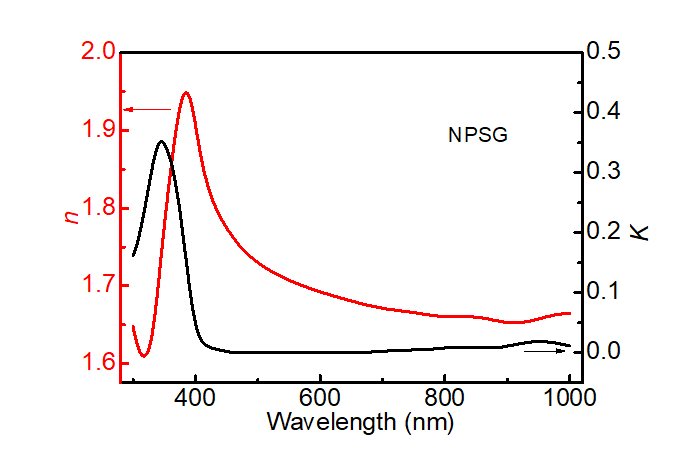


**Figure S65. The refractive index (*n*) and the extinction coefficient (*K*) of NPSG in the wavelength range of 300~1000 nm.**


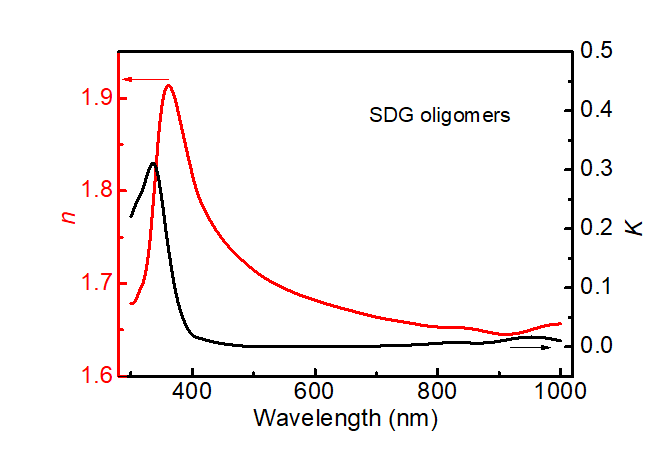


**Figure S66. The refractive index (*n*) and the extinction coefficient (*K*) of SDG in the wavelength range of 300~1000 nm.**


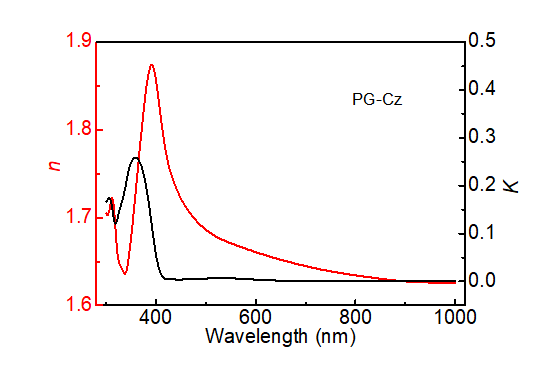


**Figure S67. The refractive index (*n*) and the extinction coefficient (*K*) of PG-Cz in the wavelength range of 300~1000 nm.**


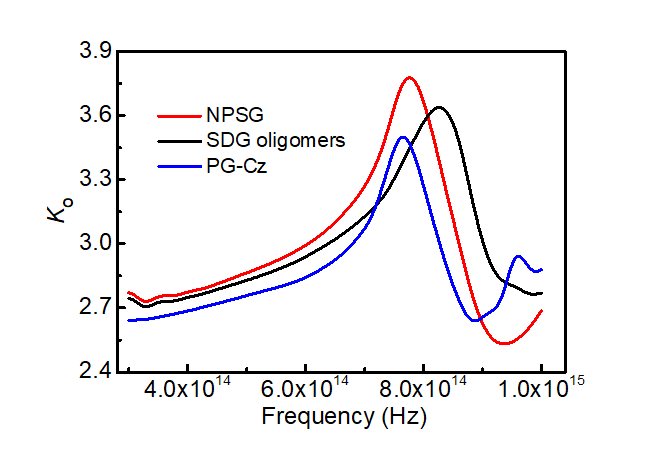


**Figure S68. The optical dielectric constant (*k*_o_) of NPSG, SDG oligomers and PG-Cz in the high frequency range of 3 × 10^14^ ~ 1 × 10^15^ Hz.** The calculation of *k*_o_ was based on the **equation ES11**: *k*_o_ = *n*^2^ – *K*^2^.


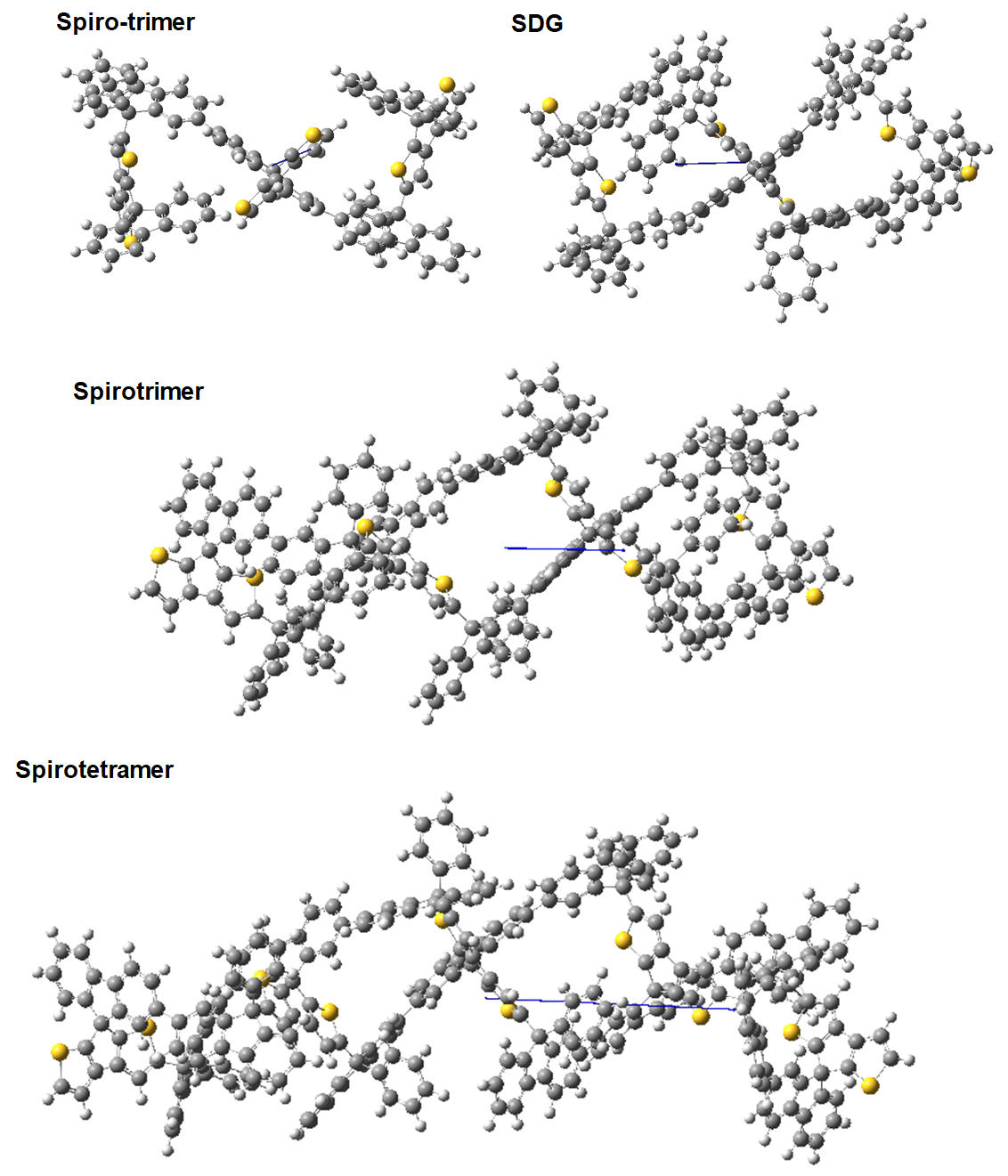


**Figure S69. The direction of dipole moment (marked in blue lines) of spiro-trimer, SDG, spirotrimer and spirotetramer.** Especially for spirotetramer, the dipole direction is approximately along the polygrid main-chains. Although we cannot simulate the dipole direction of NPSG, it is deduced that the dipole direction can be maintained when increasing the degree of polymerization, based on the prerequisite of ultralong persistence length that can sustain the single conformation in rigid rod-like mode rather than the random coil-like chains.


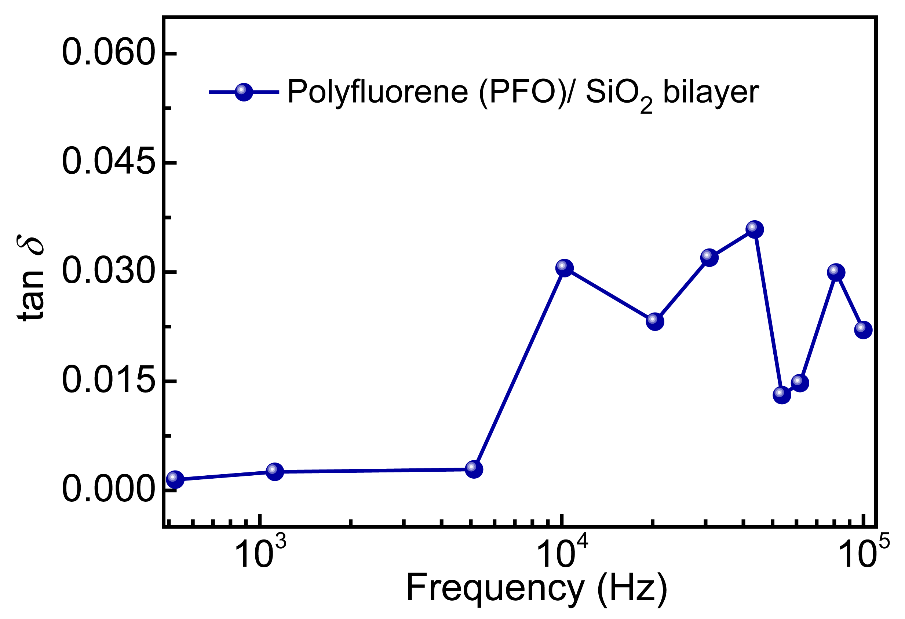


**Figure S70. The dielectric loss tangent (tan *δ*) of polyfluorene/SiO_2_ bilayer.**

**Figure S71.** **AFM image of polystyrene (PS) and polystyrene-NPSG doping films.** (a) Height image of pure PS layer. (b) Height image of pentacene layer deposited onto the pure PS layer, whose phase image is shown in (c). (d) Height image of PS-NPSG doping layer (PS:NPSG = 10:1). (e) Height image of pentacene layer deposited onto such doping layer (PS:NPSG = 10:1), whose phase image is shown in (f). (g) Height image of PS-NPSG doping layer (PS:NPSG = 5:1). (h) Height image of pentacene layer deposited onto such doping layer (PS:NPSG = 5:1), whose phase image is shown in (i).

In Figure S71a, the pure polystyrene (PS) film shows a smooth surface with a little particles, which displays a low root-of-square roughness (*R*_q_) of 0.54 nm. Further depositing pentacene layer onto polystyrene layers through physical vapour deposition technology, we observed the polycrystalline morphology of pentacene with a relative lower *R*_q_ = 4.58 nm (Figure S71b). When doping NPSG in PS layer (NPSG:PS = 1:10), we observed the generation of nanoparticles (Figure S71d) that suggest the presence of phase separation between NPSG and PS. The roughness is increased to *R*_q_ = 3.44 nm. Based on such film morphology, the deposition of pentacene layers can relatively maintain the crystalline domains but increase the roughness to *R*_q_ = 6.90 nm (Figure S71e). Further increasing the NPSG-doping amount to PS:NPSG = 5:1 results in the formation of more and larger nanoparticles, which also causes higher roughness *R*_q_ = 7.70 nm (Figure S71g). Meanwhile, if depositing pentacene layer, the larger particle morphology was observed (Figure S71h), as confirmed by its phase image (Figure S71i). The roughness of film morphology containing pentacene layer is also increased to 12.10 nm. According to above results, doping NPSG results in the higher roughness that is not favorable to the enhancement of carrier mobility. Thus, the improvement of carrier mobility may be not related to the improved film morphology.

**10. Optoelectronic properties** **of NPSG**

| Molecules | Absorption  (nm) | Emission  (0-0)  (nm) | Emission  (0-1)  (nm) | Stokes shift (eV) | Optical Bandgap  (eV) |
| --- | --- | --- | --- | --- | --- |
| STF-DOH | 356 | 404 | 426 | 0.41 | 3.18 |
| SDG | 335 | 397 | 413 | 0.58 | 3.33 |
| NPSG  (*DP*_n_ ≈ 5) | 337 | 407 | 421 | 0.62 | 3.15 |
| NPSG  (*DP*_n_ ≈ 16) | 341 | 408 | 426 | 0.60 | 3.13 |
| NPSG  (*DP*_n_ ≈ 26) | 343 | 415 | 429 | 0.62 | 3.11 |
| Terfluorene | 350 | 394 | 416 | 0.39 | 3.20 |
| Ladder digrid | 341 | 395 | 416 | 0.50 | 3.26 |
| Ladder grid | 310, 330 | 371 | 404 | 0.42 | 3.39 |
| Tetrafluorene | 364 | 406 | 428 | 0.36 | 3.10 |
| Polygrid PG-Cz | 359 | 404 | 428 | 0.38 | 3.10 |
| Polyfluorene | 390 | 419 | 443 | 0.22 | 2.99 |

**Table S3. The photophysical parameters of NPSG solutions.** The photophysical features of other molecules, including terfluorene [*59*], ladder digrid [*2*], ladder-type grid unit [*60*], tetrafluorene [*61*], polygrid PG-Cz [*58*] and polyfluorene [*36*], are also provided as the standards.


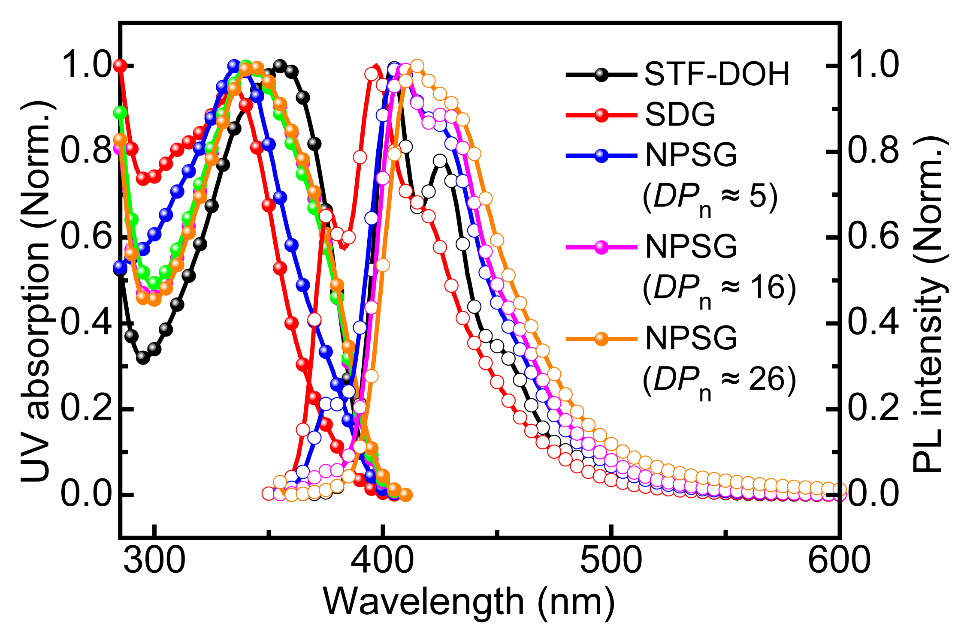


**Figure S72. UV-PL spectra of STF-DOH and NPSG (in CHCl_3_ solvent) with various *DP*_n_.**


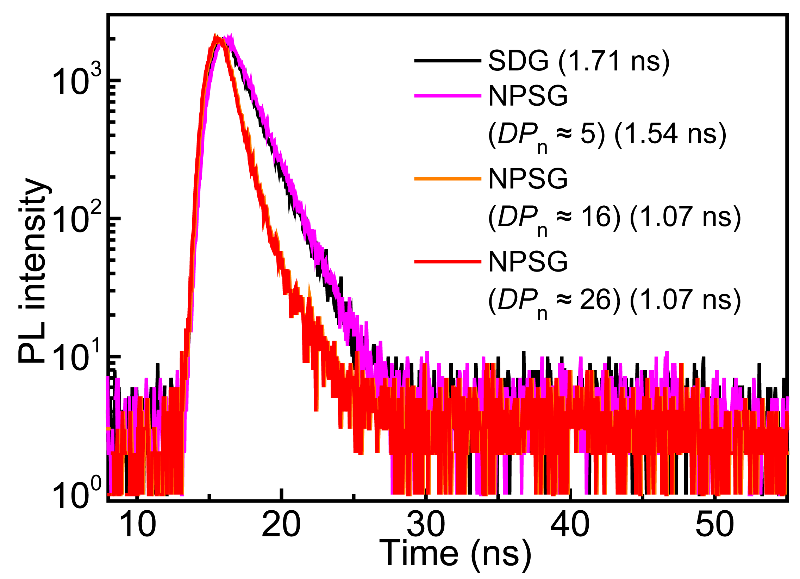


**Figure S73. Transition decay and lifetimes of NPSG in CHCl_3_ solvent.**


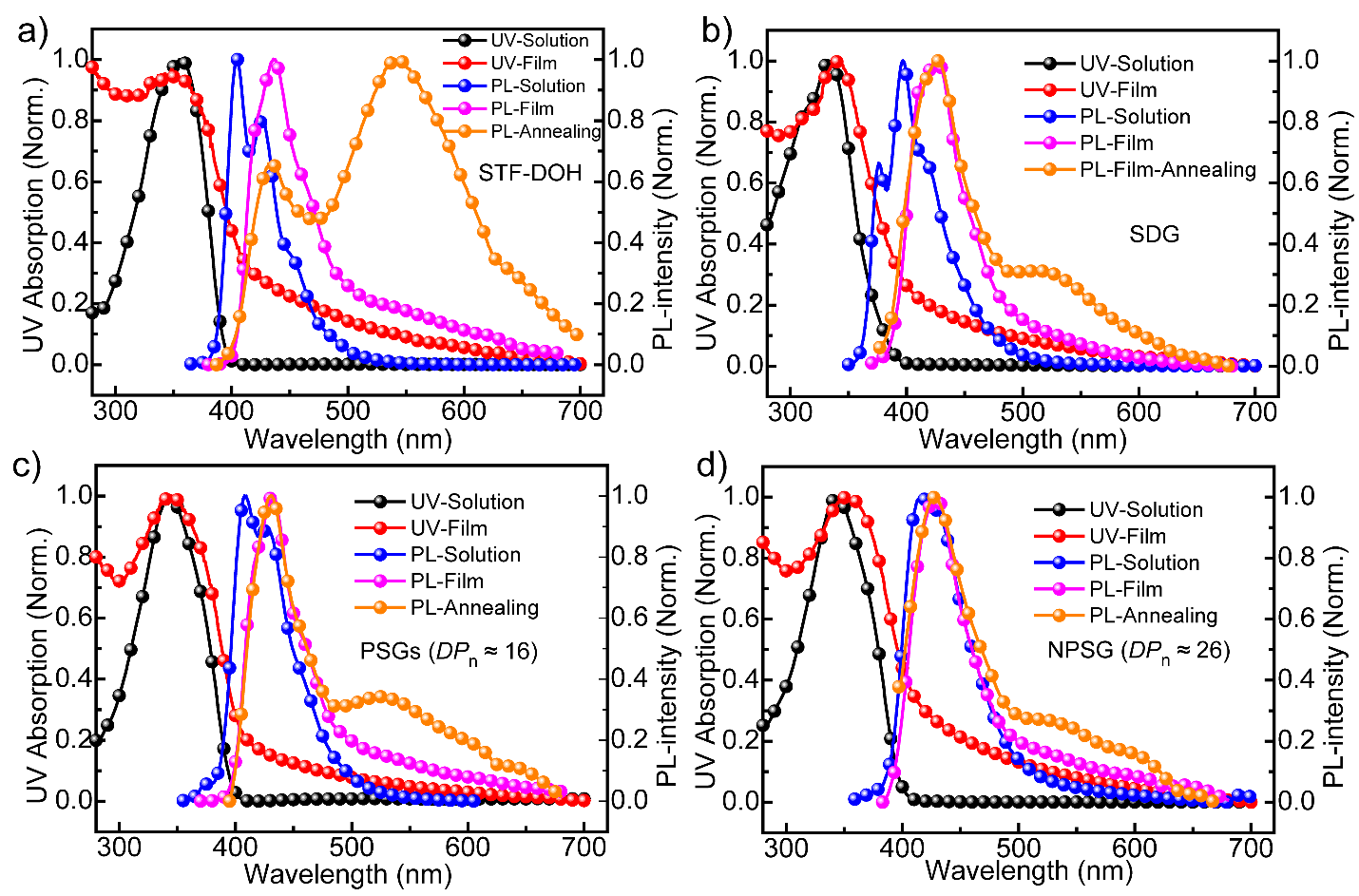


**Figure S74. Photophysical properties of STF-DOH, SDG and NPSG.** (a) STF-DOH; (b) SDG; (c) NPSG (*DP*_n_ ≈ 16); (d) NPSG (*DP*_n_ ≈ 26). The solvent was selected as DCE, where UV absorption spectra are shown in black dots and lines; PL spectra are shown in blue dots and lines. The films (their UV absorption spectra are shown in red dots and lines; PL spectra are shown in pink dots and lines) were prepared via spin-coating of solution (the concentration of 8 mg/ml) with DCE solvent. The PL-annealing spectra (marked in orange dots and lines) represent the photoluminescence of the annealed film under air environment (260 ^o^C, in 4 h).

| Molecules | Absorption  (nm) | Emission  (0-0)  (nm) | Emission  (0-1)  (nm) | Emission intensity  (> 500 nm) |
| --- | --- | --- | --- | --- |
| STF-DOH | 354 | 421 | 436 | 165% |
| SDG | 341 | 410 | 428 | 31% |
| NPSG  (*DP*_n_ ≈ 16) | 345 | 415 | 430 | 34.5% |
| NPSG  (*DP*_n_ ≈ 26) | 352 | 419 | 427 | 27% |

**Table S4. The photophysical parameters of NPSG film (Spin-coated from DCE solution).** The film was heated at 100 ^o^C (in 30 min) to remove DCE solvent. The intensity of emission (> 500 nm) after the annealing process (under 260 ^o^C in 40 min, under air atmosphere) were versus the intensity of intrinsic 0-1 emission.

**11. Amorphous properties of NPSG film**

**
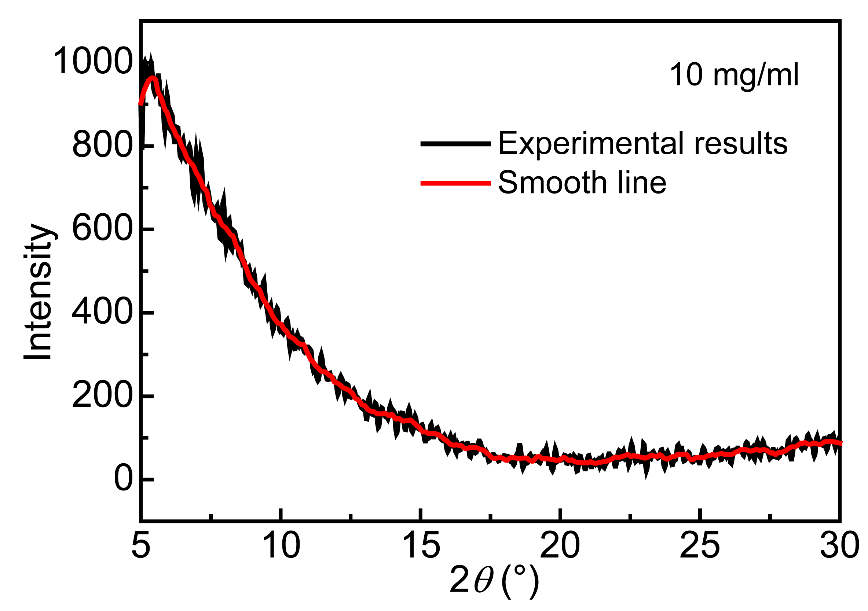
**

**Figure S75. The X-ray diffraction (XRD) of spin-coated NPSG film.** There is almost no crystalline diffraction peak. The film was spin-coated on the silicon wafer.

**
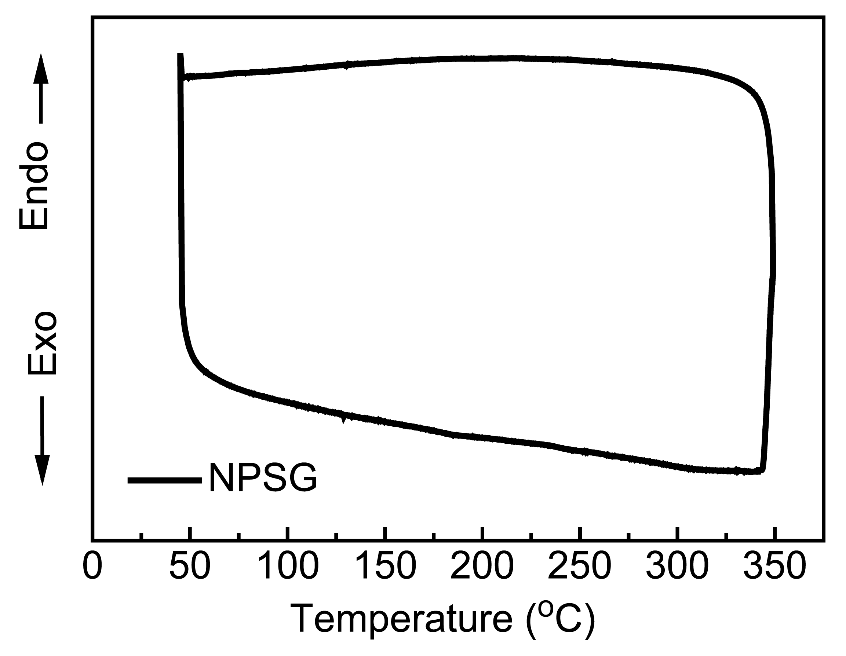
**

**Figure S76. The DSC characterization of NPSG**. There is no exothermic signals assigning to crystalline process and no endothermic signals for melting process. These results firmly indicate that no crystalline behaviors occur for NPSG nanochains, which are sustained in amorphous states.

**12. Electric properties of NPSG film**

**
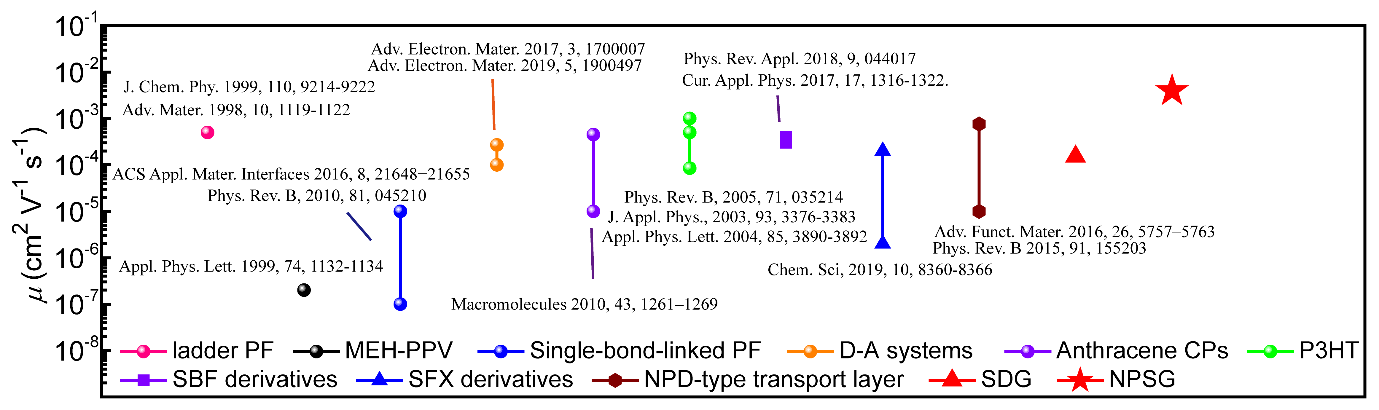
**

**Figure S77. The zero-field carrier mobility of various organic small molecules and polymers in amorphous state.** Ladder PF is defined as the conjugated ladder-type polyfluorenes [*62, 63*]; MEH-PPV is poly(-2-methoxy,5-(2′-ethyl-hexoxy)-*p*-phenylene vinylene) [*64*]; Single-bond-linked PF includes the copolyfluorenes and polydiarylfluorenes [*65, 66*]; D-A systems are blending systems containing donor and acceptor building blocks with thiophene groups [*6, 67*]; Anthracene CPs are anthracene-based conjugated polymers [*68*]. P3HT is the poly(3-hexylthiophene) (*69-71*); SBF derivatives are spirobifluorene-based derivatives [*72, 73*]; SFX derivatives are spirofluorenexanthene-based derivatives [*74*]; NPD-type transport layer [*75, 76*] covers the transport layer such as *N4,N4′*-di(biphenyl-3-yl)-*N4,N4′*-diphenylbiphenyl-4,4′-diamine (mBPD), *N1,N4*-di(naphthalen-1-yl)-*N1,N4*-diphenylbenzene-1,4-diamine (NNP), 1,1-bis-(4,4′-diethylaminophenyl)-4,4-diphenyl-1,3,butadinene (DEPB), *N,N′-*bis(1-naphthyl)-*N,N′-*diphenyl-1,1′-biphenyl-4,4′-diamine (NPD), *N,N′-*diphenyl- *N,N′-*bis-(3-methy-lphenylene)-1,10-diphenyl-4,40-diamine (TPD), *N,N′*-bis-[9,9-dimethyl-2-fluorenyl]-*N,N′*-diphenyl-9,9-dimethylfl uorene-2,7-diamine (pFFA), tetracene (TET) and pentacene (PEN).


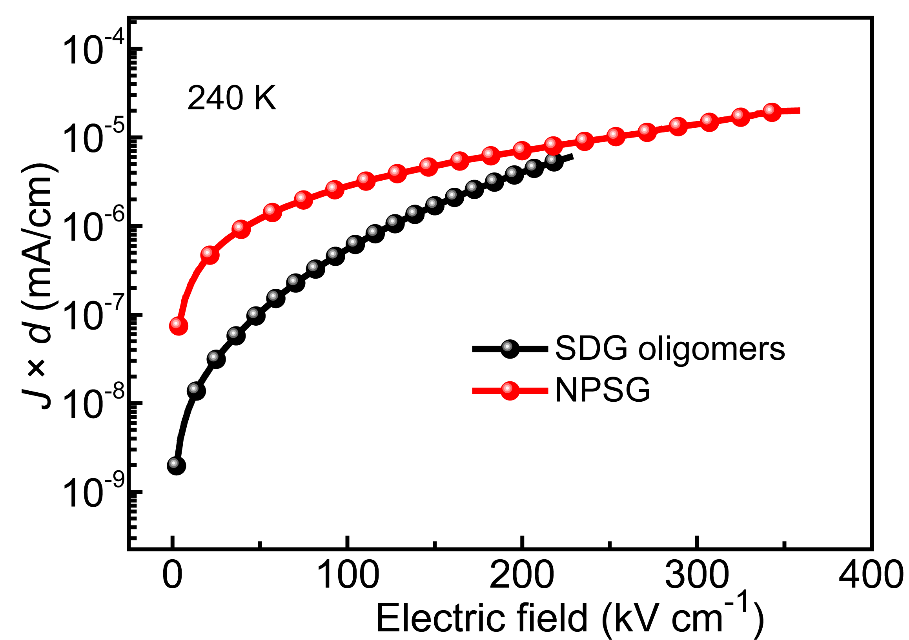


**Figure S78. The dependence of space-charged-limited current density (*J*) × (film thickness of NPSG/SDG) *d* on the electric field, under the temperature of 240 K.**


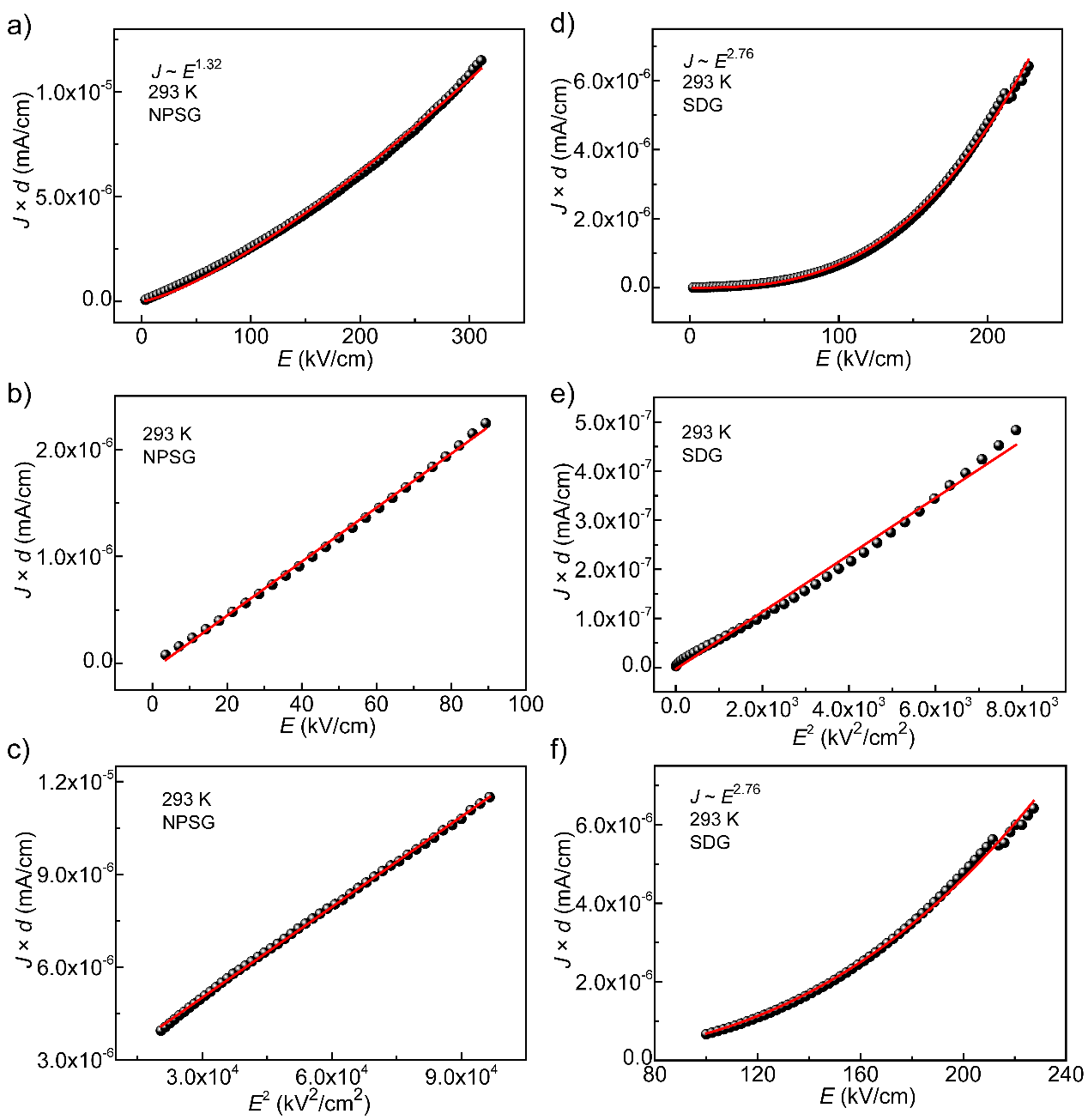


**Figure S79. Fitting of SCLC curve of NPSG and SDG under the temperature of 293 K.** (a) The total spectra of SCLC of NPSG. The fitting function is *J* ~ *E^v^*, where *J*, *E* and *v* are denoted as the current density, electric field and the power exponent, respectively. (b) The partial spectra of SCLC curve of NPSG within the small electric field region of *E* = 0~90 kV/cm, where the SCLC curve shows the scaling law *J* ~ *E^1^*, similar to the feature of Ohmic conductivity. (c) The partial spectra of SCLC curve of NPSG within the large electric field region of *E* = 150~350 kV/cm. The fitting function is used as the *J* ~ *E^2^*. (d) The total spectra of SCLC of SDG. The fitting function is used as the *J* ~ *E^v^*. (e) The partial spectra of SCLC curve of SDG within the small electric field region of *E* = 0~100 kV/cm, where the SCLC curve shows the scaling law *J* ~ *E^2^*. (f) The partial spectra of SCLC curve of SDG within the large electric field region of *E* = 100~230 kV/cm. The fitting function is used as the *J* ~ *E^v^*.

In Figure S79a, the current density curve of NPSG at 293 K exhibits a lower scaling law of *J* ~ *E*^1.32^ in the whole spectra, where *J* and *E* are denoted as the current density and electric field, respectively. According to the corresponding literature, the power exponent lower than 2 is partly attributed to the contribution of Ohmic conductivity behavior with the scaling low *J* ~ *E* [*77*], which is generally observed within the low *E* region. Thus, we separately fitted such current density curve at *E* = 0~90 kV/cm region (Figure S79b) and *E* > 150 kV/cm region (Figure S79c), respectively. In Figure S79b, the current density at *E* = 0~90 kV/cm does matches the Ohmic conductivity law with *J* ~ *E* relationships. In Figure S79c, the current density at *E* > 150 kV/cm also perfectly satisfies the linear fitting relationships between *J* and *E^2^*, with the coefficient of determination *R*^2^ = 0.995. These features are consistent with the transformation from Ohmic to trap-free space charge limited current mode [*77*]. For the calculation of hole mobility (*μ*_h_), we used trap-free mode of SCLC method (*J* ~ *E*^2^, the film thickness is also considered) on high *E* region (*E* > 150 kV/cm, to ensure the power exponent of 2), which can afford *μ*_h_ with relatively low deviations. In addition, we do not observed the power exponent higher than 2, which indicates the absence of deep-traps. In contrast, for SDG film under the temperature of 293 K, such current density meets the scaling law of *J* ~ *E*^2.76^ in the whole *E* region (Figure S79d). Especially, the low *E* region (*E* = 0~90 kV/cm) shows a scaling law of *J* ~ *E*^2^ (Figure S79e), as the trap-free region to calculate the zero-field hole mobility. The Figure S79e shows that the high E region (*E* > 100 kV/cm) has a high power exponent of 2.76 that suggests the existence of deep-traps, with a larger width of deep-traps in density of states [*78*].


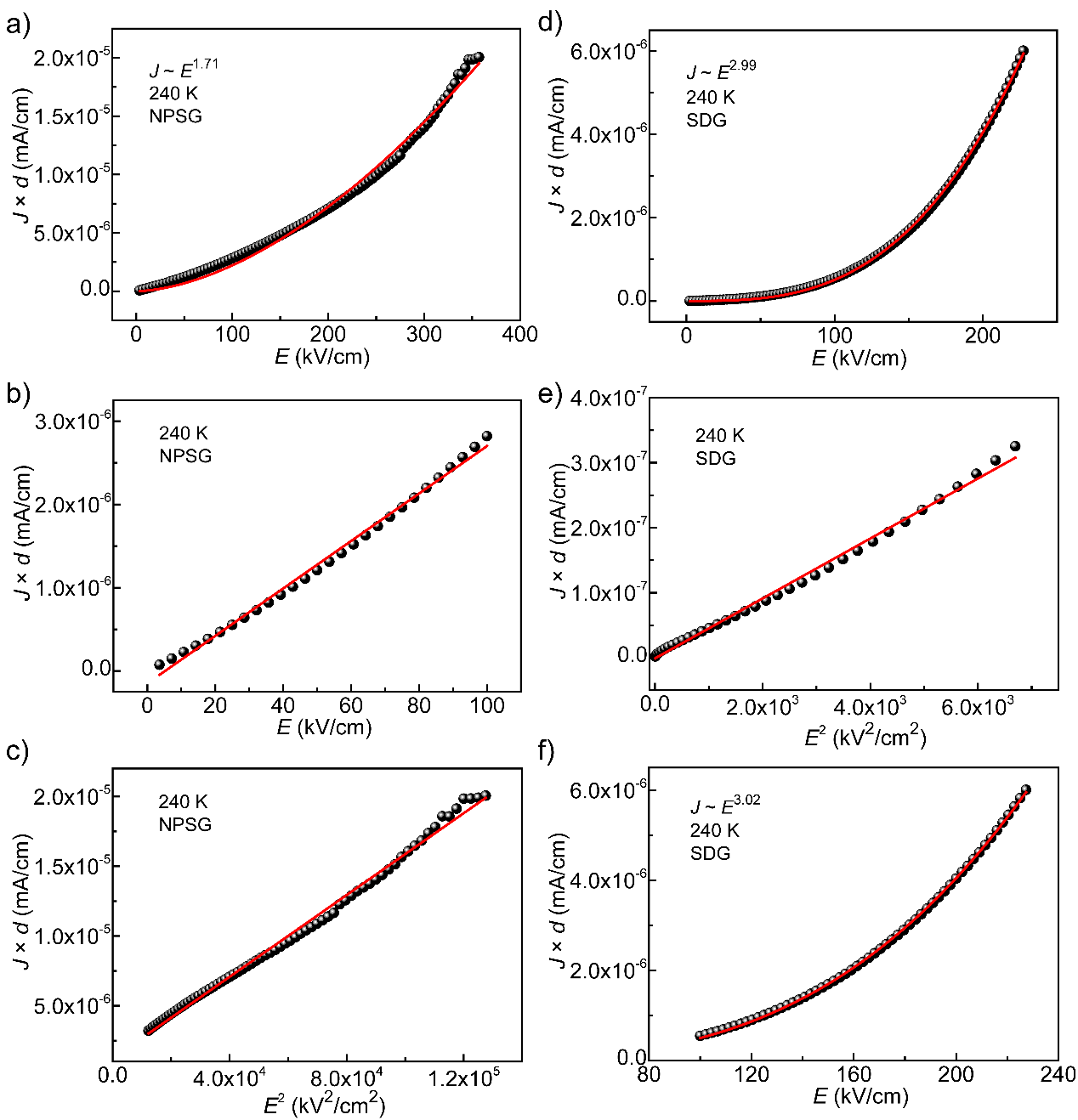


**Figure S80. Fitting of SCLC curve of NPSG and SDG under the temperature of 240 K.** (a) The total spectra of SCLC of NPSG. The fitting function is *J* ~ *E^v^*. (b) The partial spectra of SCLC curve of NPSG within the small electric field region of *E* = 0~100 kV/cm, showing the scaling law *J* ~ *E^1^*. (c) The partial spectra of SCLC curve of NPSG within the large electric field region of *E* = 150~350 kV/cm. The fitting function is used as the *J* ~ *E^2^*. (d) The total spectra of SCLC of SDG. The fitting function is used as the *J* ~ *E^v^*. (e) The partial spectra of SCLC curve of SDG within the small electric field region of *E* = 0~100 kV/cm, where the SCLC curve shows the scaling law *J* ~ *E^2^*. (f) The partial spectra of SCLC curve of SDG within the large electric field region of *E* = 100~230 kV/cm. The fitting function is used as the *J* ~ *E^v^*.

Under the testing temperature of 240 K, SCLC of NPSG possesses a power exponent of 1.71 in the whole *E* region (Figure S80a), as is higher than the power exponent (*J* ~ *E*^1.32^) under 293 K. This result is consistent with the fact that lowering the temperature is capable of increasing deep-traps and electric-field dependence [*78*]. In Figure S80b, the feature of Ohmic conductivity law with *J* ~ *E* relationships can also be maintained within the region *E* = 0~100 kV/cm. In Figure S80c, we also observed the linear fitting relationships between *J* and *E^2^* at *E* > 110 kV/cm, as the defect-free region used for calculating the hole mobility. In contrast, under the temperature of 240 K, SDG film exhibits the high power exponent m = 2.99 in the whole *E* region (Figure S80d). By deeper analysis in Figure S80e, we found that the current density matches the scaling law of *J* ~ *E*^2^ (Figure S80e) in trap-free mode within low *E* region (*E* = 0~90 kV/cm). At high *E* region (*E* > 100 kV/cm), the power exponent is increased to m = 3.02, indicating the presence of deep-traps (Figure S80f).


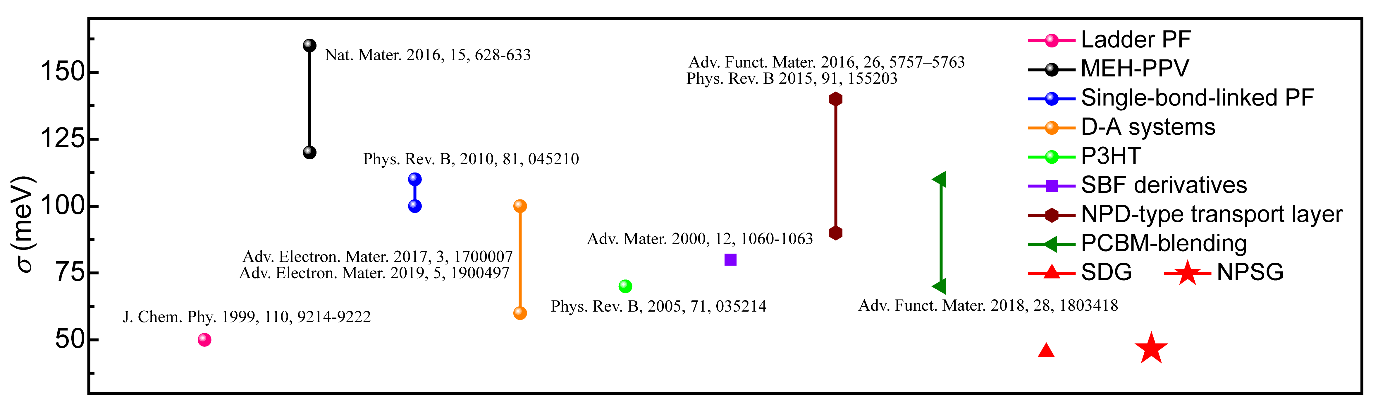


**Figure S81. The energy disorder of various organic small molecules and polymers [*6, 63, 66, 67, 69, 75, 76, 79, 80*] in amorphous state.**


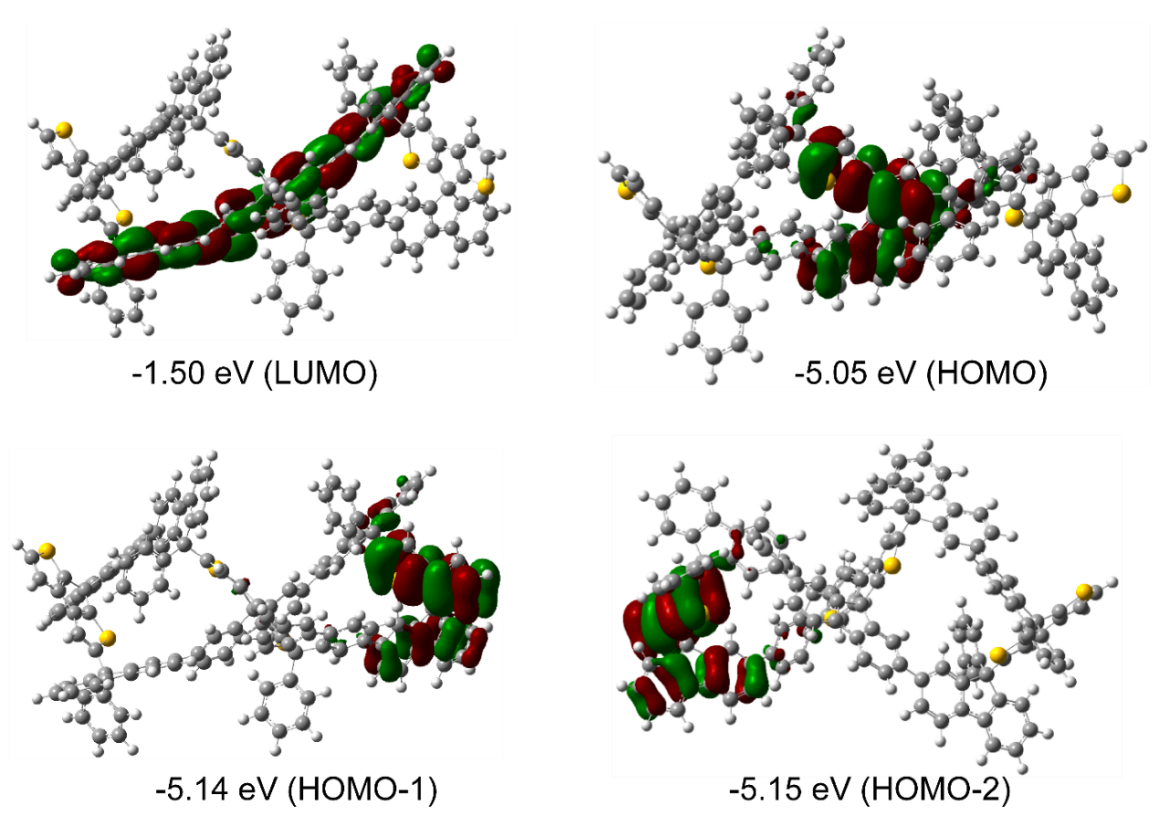


**Figure S82. The molecular orbital distribution of SDG.**


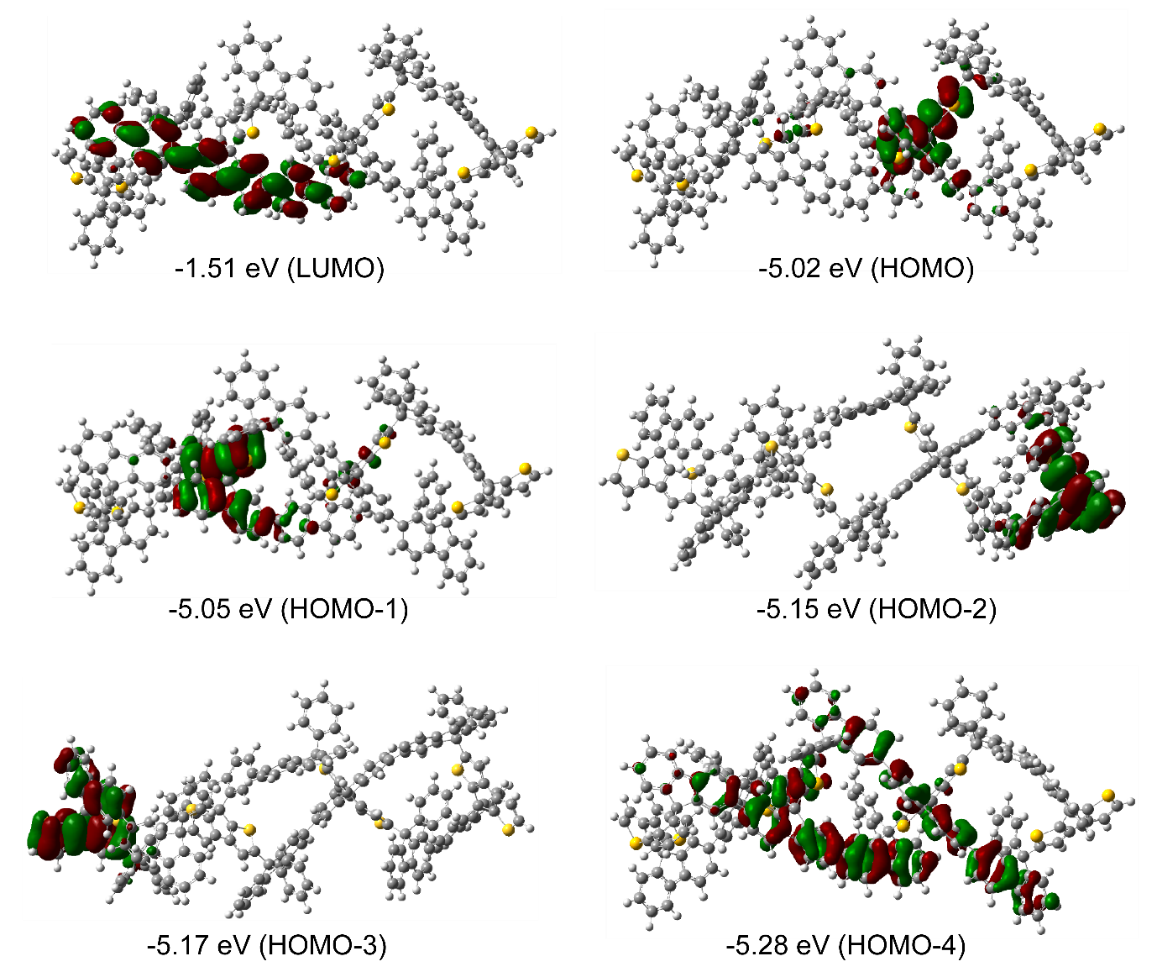


**Figure S83. The molecular orbital distribution of spirotrigrid (DP = 3 of NPSG).** The orbitals of HOMO and HOMO-1 levels are approximately to the degenerated states mutually.


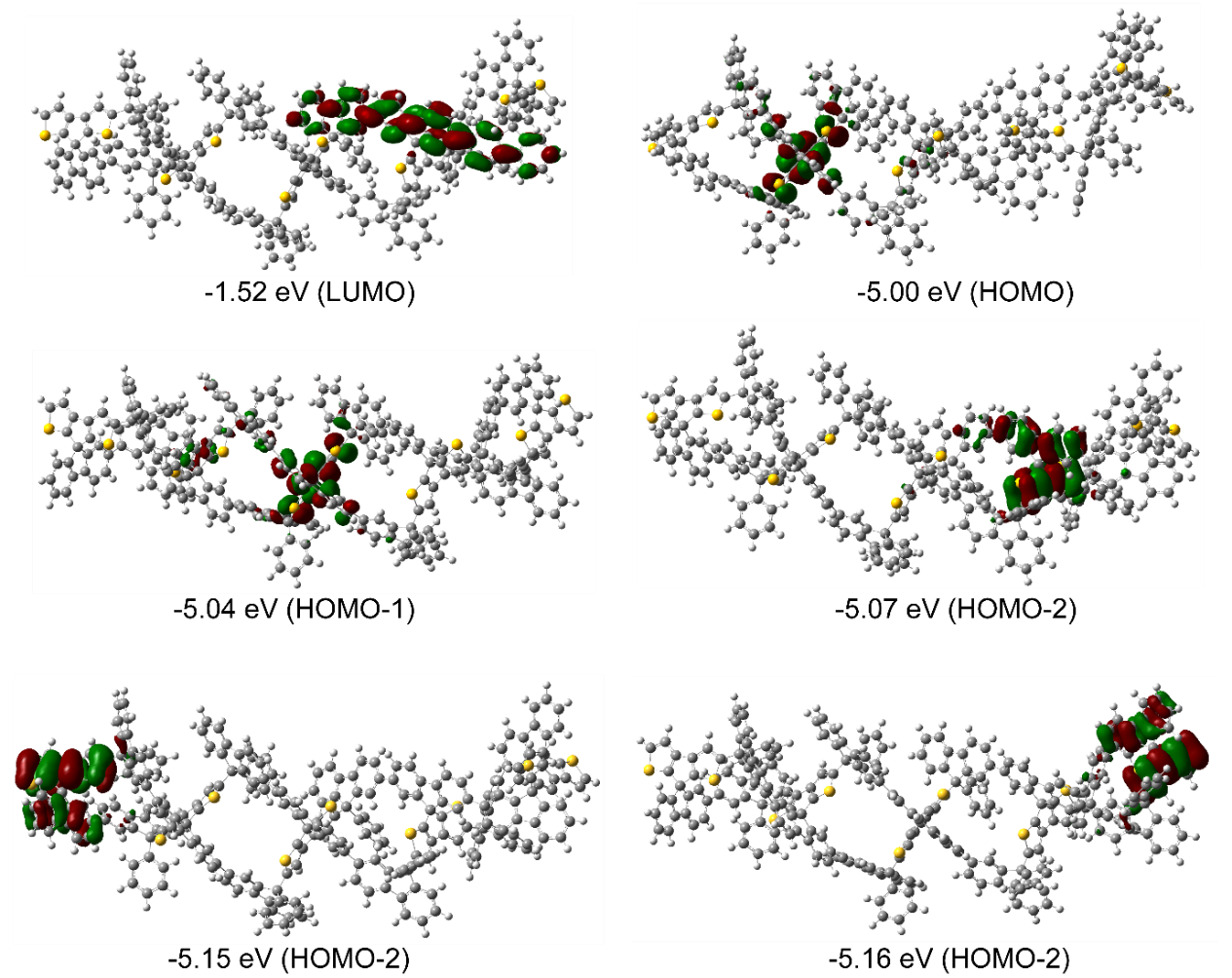


**Figure S84. The molecular orbital distribution of spirotetragrid (DP = 4 of NPSG).** The energy levels marked in red (HOMO-2, HOMO-1 and HOMO) are all approximate to degenerated states.


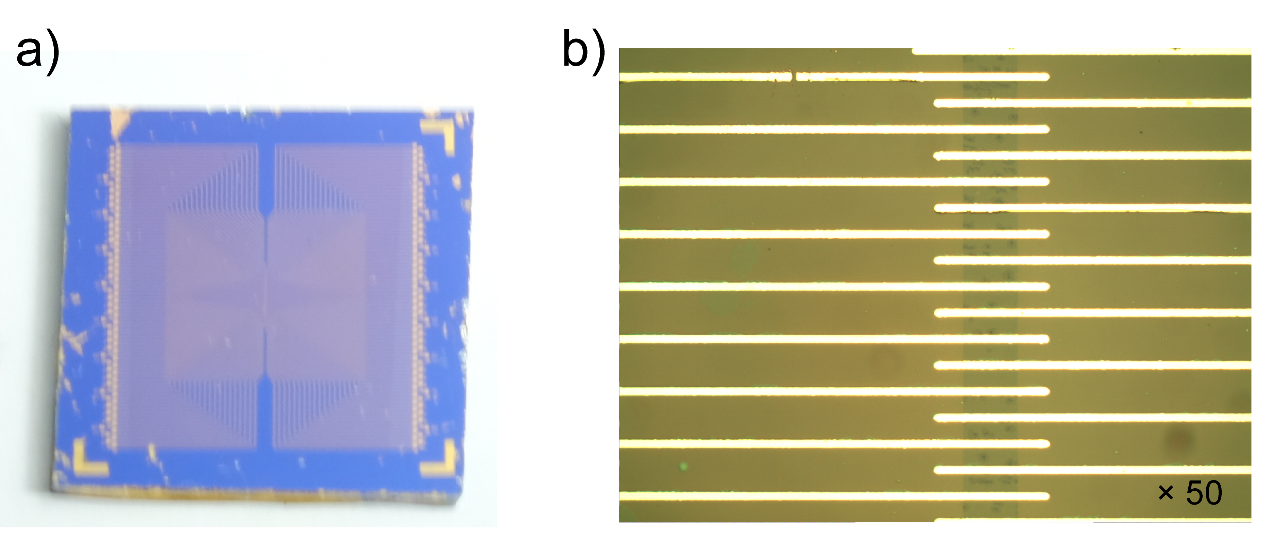


**Figure S85. A macroscopic pattern of NPSG-based molecular electronic device.** The observation of such device via optical microscopy (the magnification of 50 times) was shown in (b).


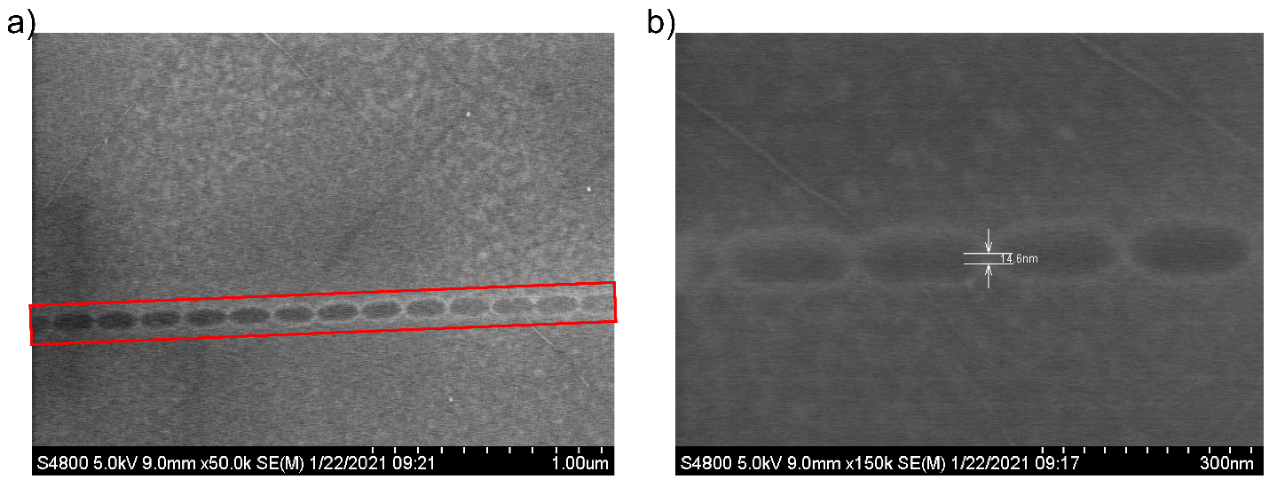


**Figure S86. The SEM imagine of molecular electronic device.** The red framework represents the graphene electrodes. An unterminated NPSG nanochain was linked between two electrodes with the length of ~14.6 nm (a). It is noted that two hydroxyl groups were remained on the chain-end fluorenol group of NPSG nanochains, which enables to perform ether linkage with graphene electrodes.

**13. NMR spectra**

**
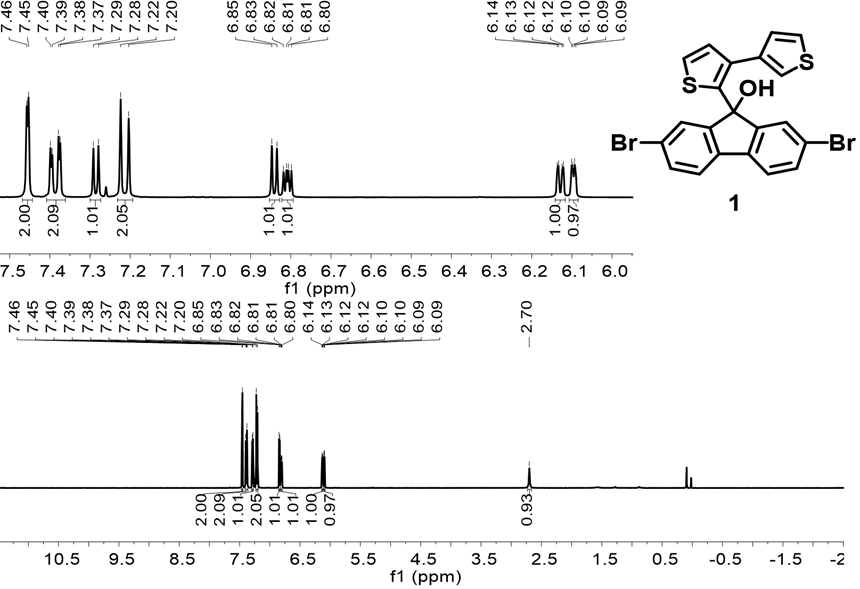
**

**Figure S87. ^1^H NMR spectra of 1**


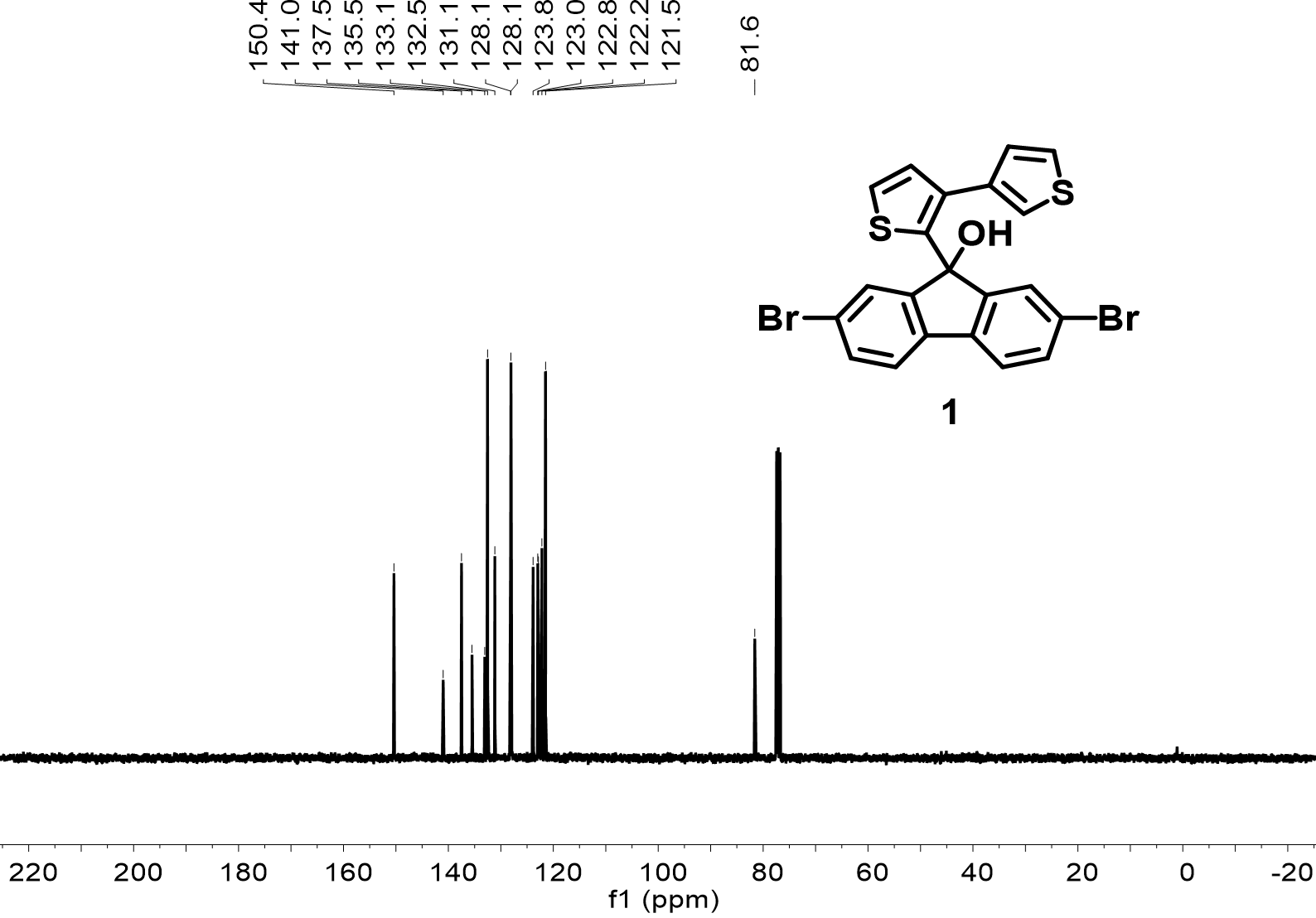


**Figure S88. ^13^C NMR spectra of 1**


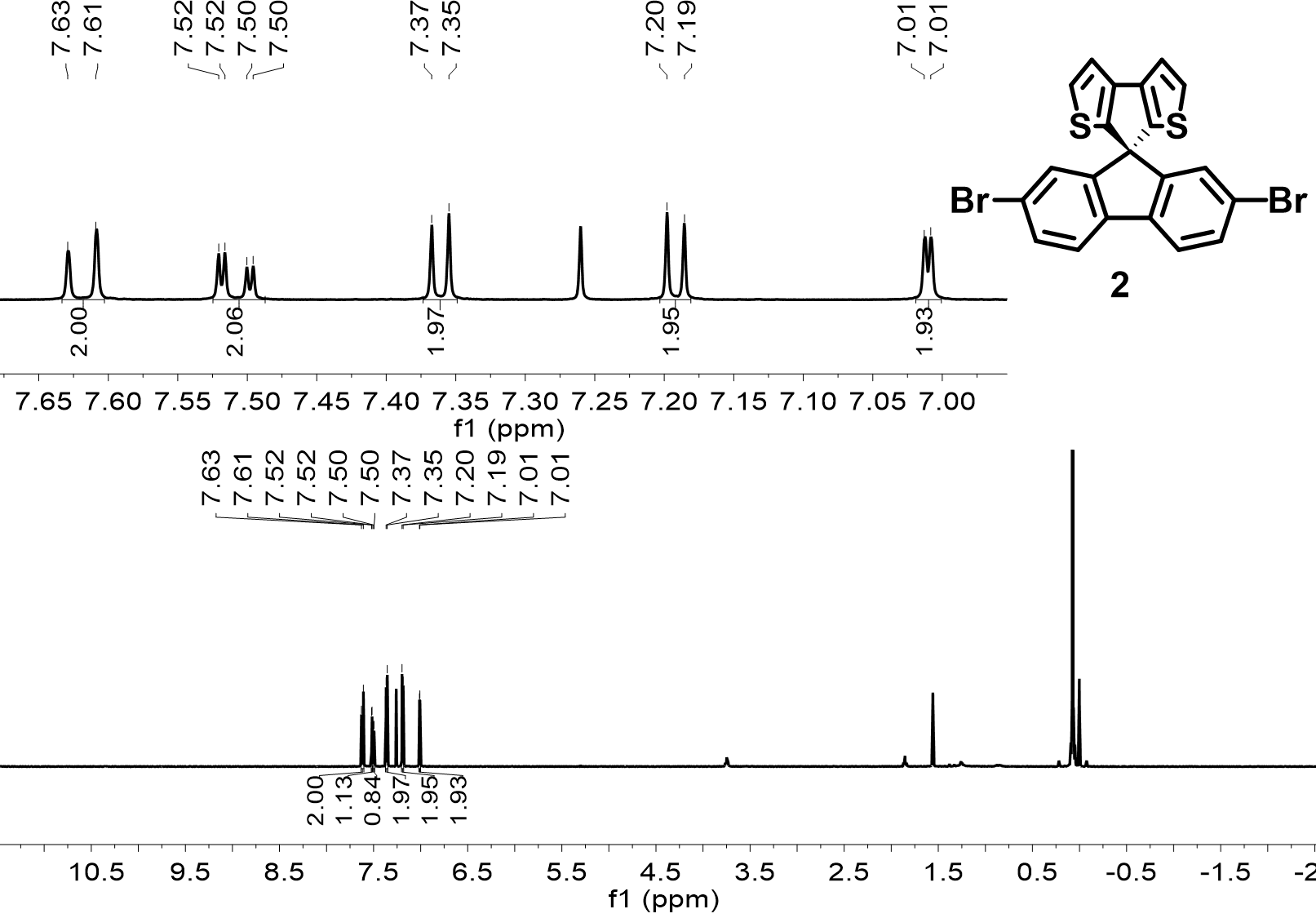


**Figure S89. ^1^H NMR spectra of 2**


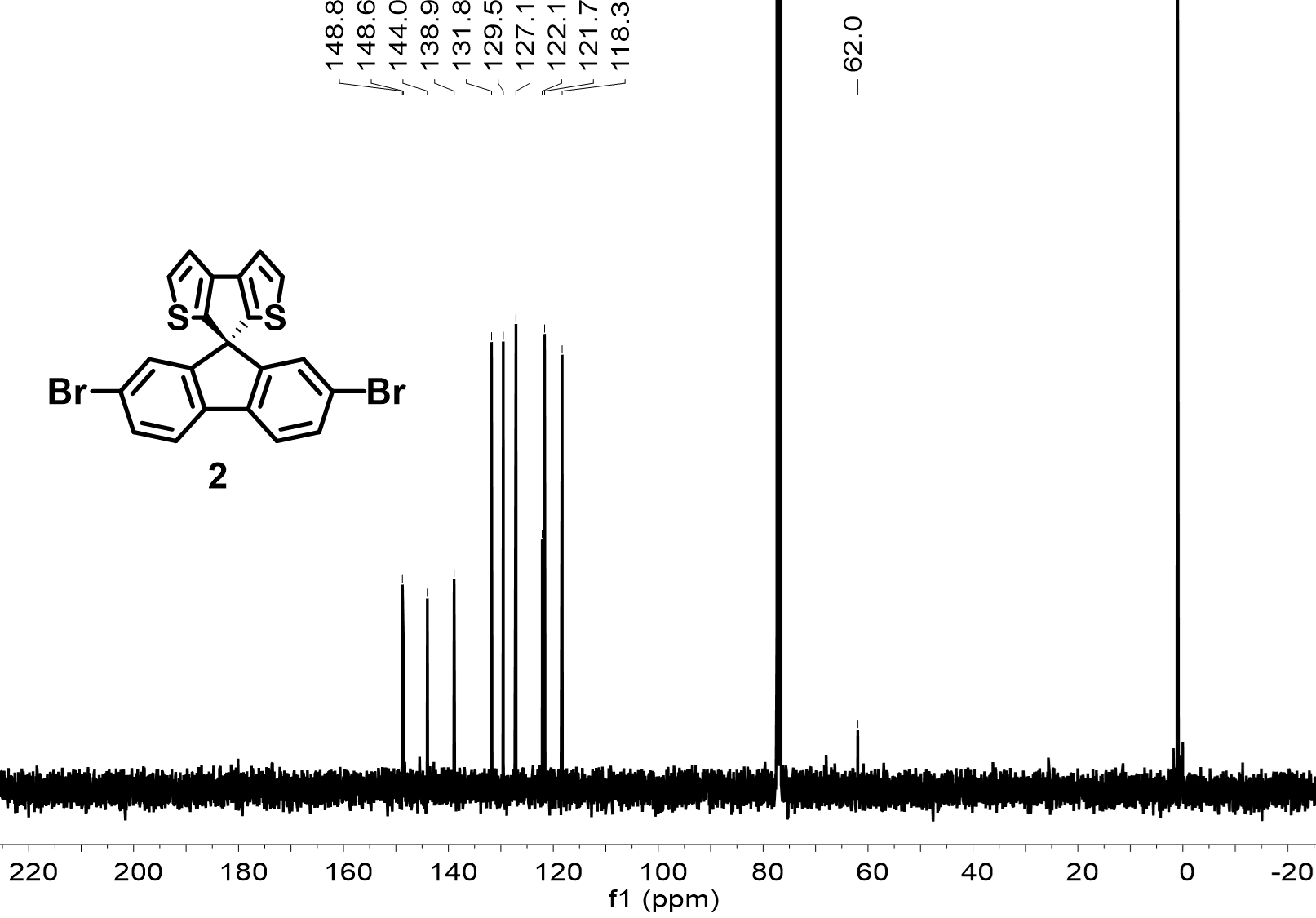


**Figure S90. ^13^C NMR spectra of 2**


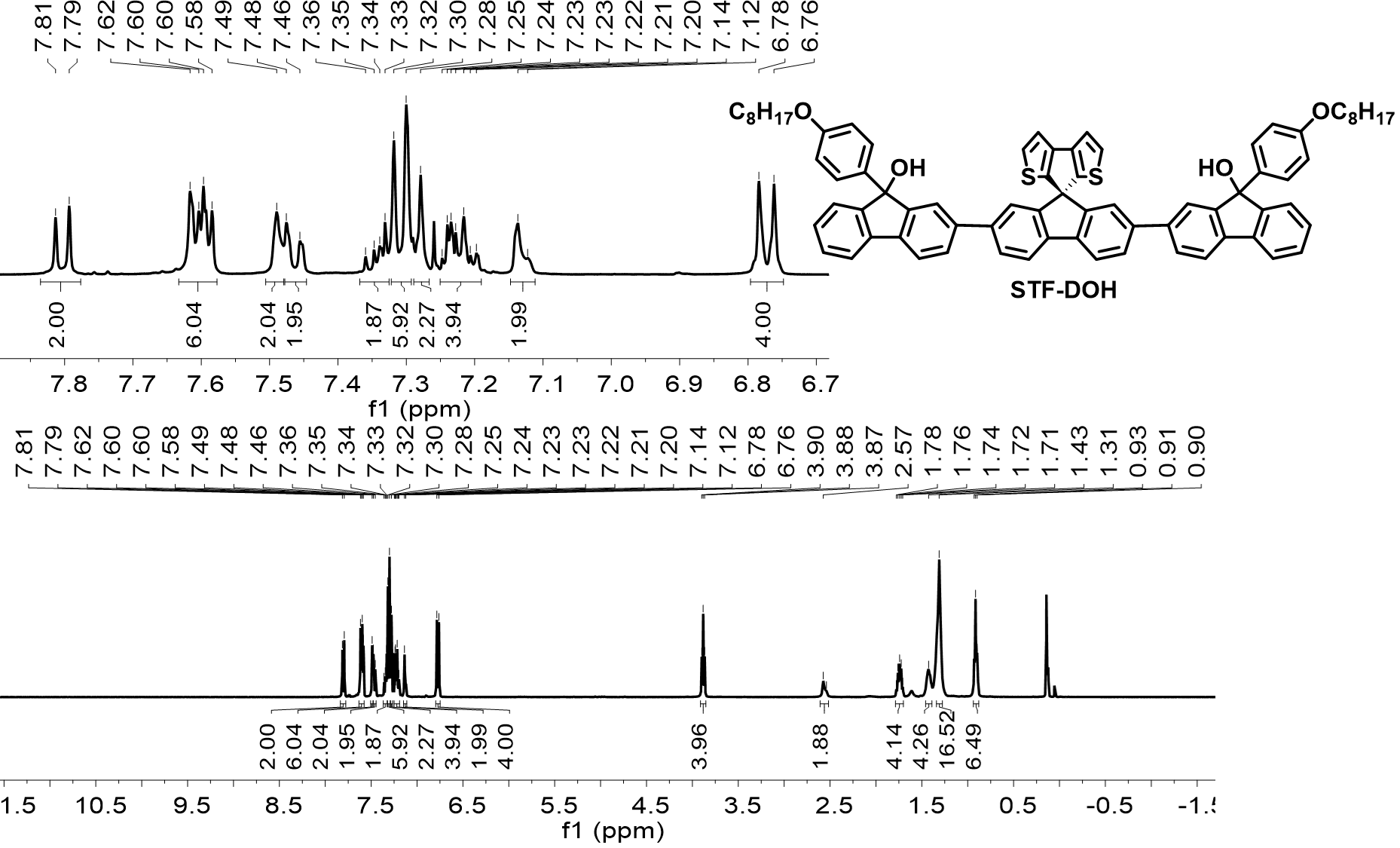


**Figure S91. ^1^H NMR spectra of STF-DOH**


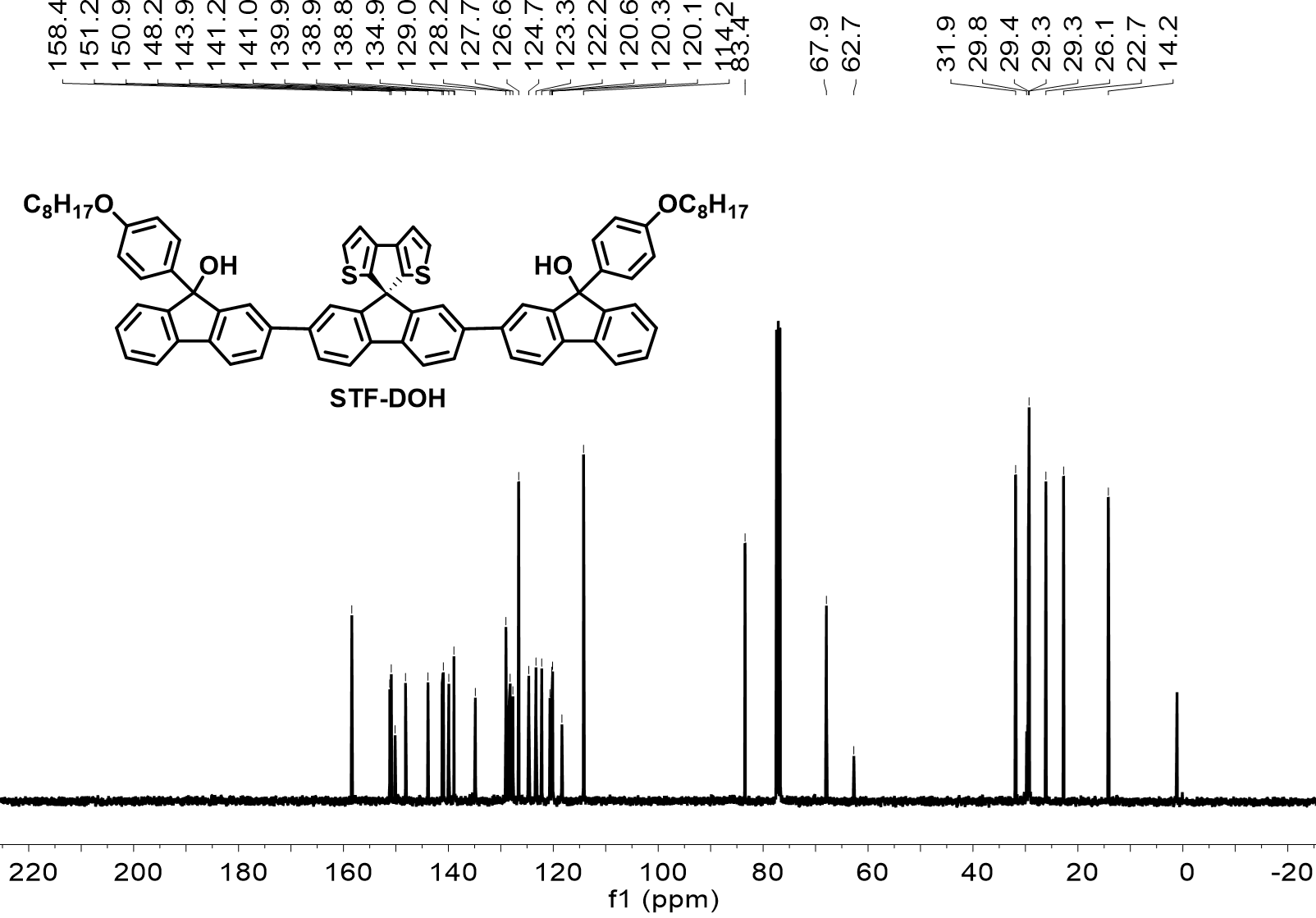


**Figure S92. ^13^C NMR spectra of STF-DOH**


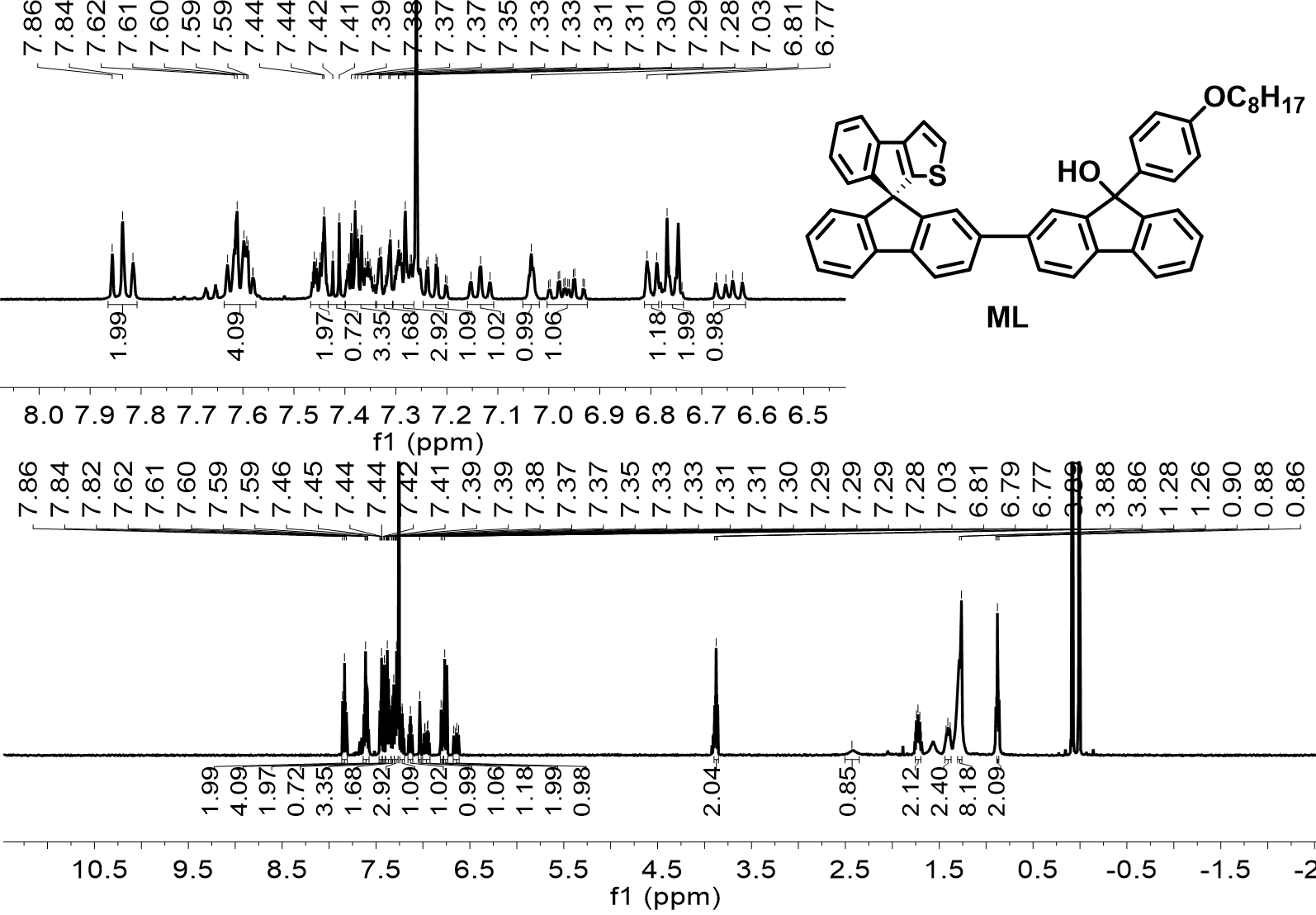


**Figure S93. ^1^H NMR spectra of ML**


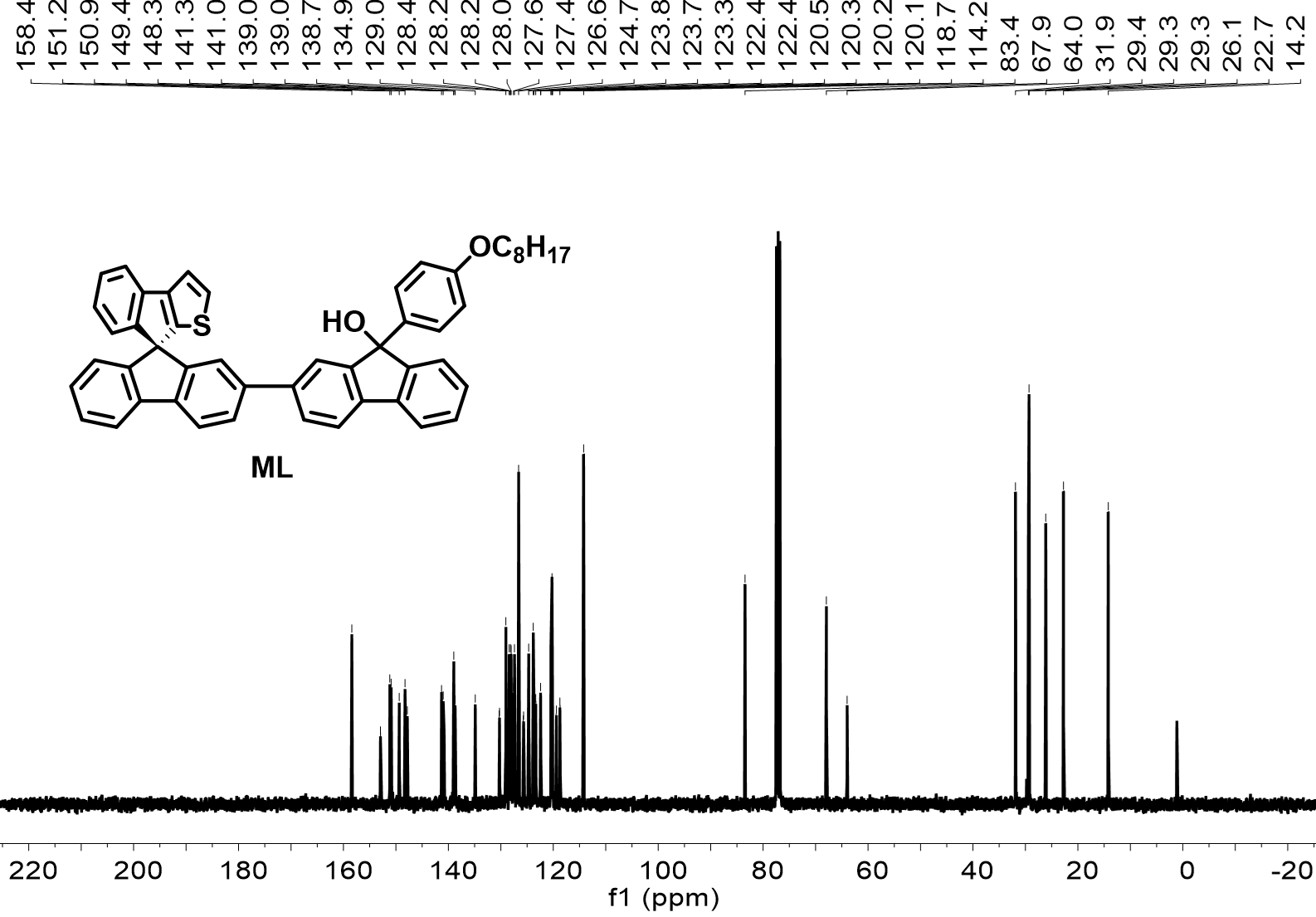


**Figure S94. ^13^C NMR spectra of ML**


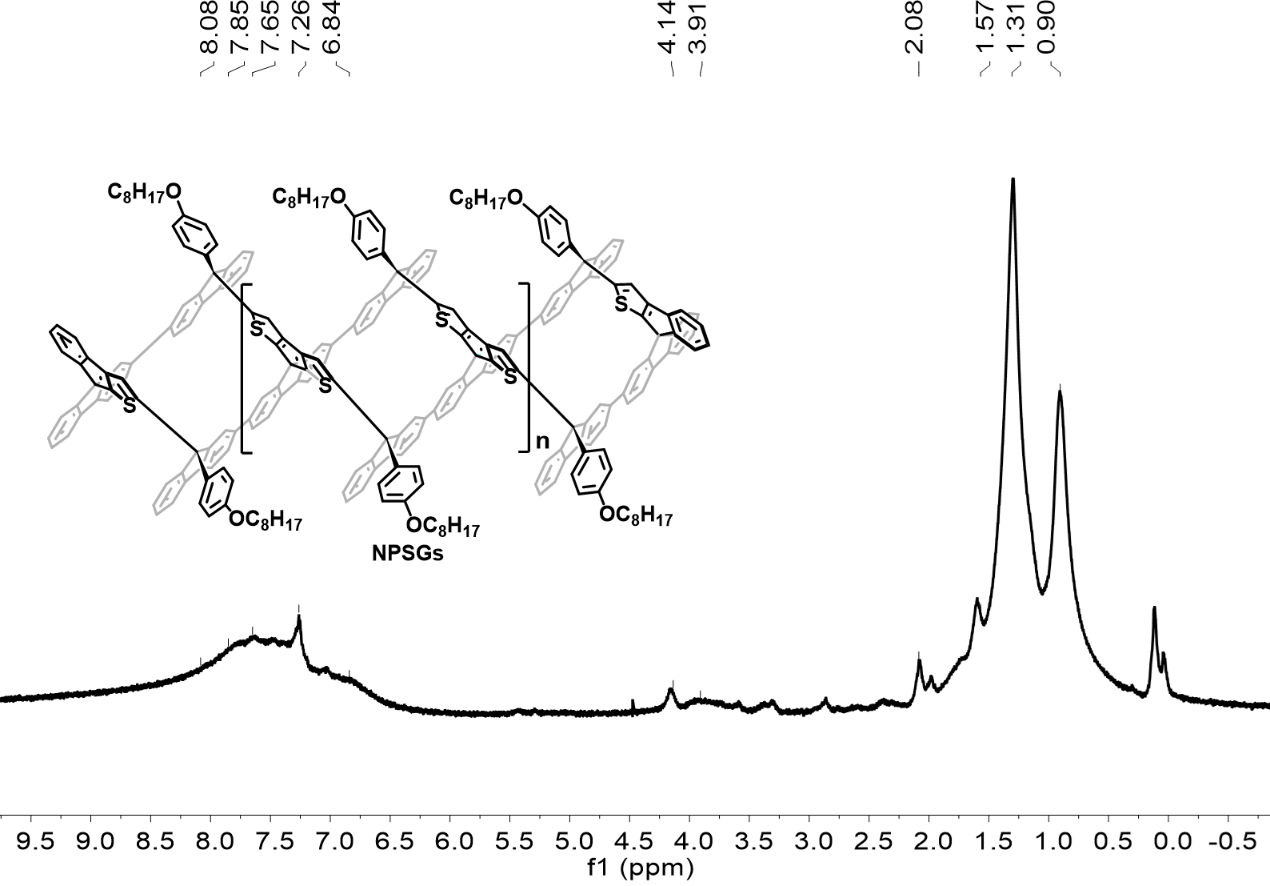


**Figure S95. ^1^H NMR spectra of NPSG**


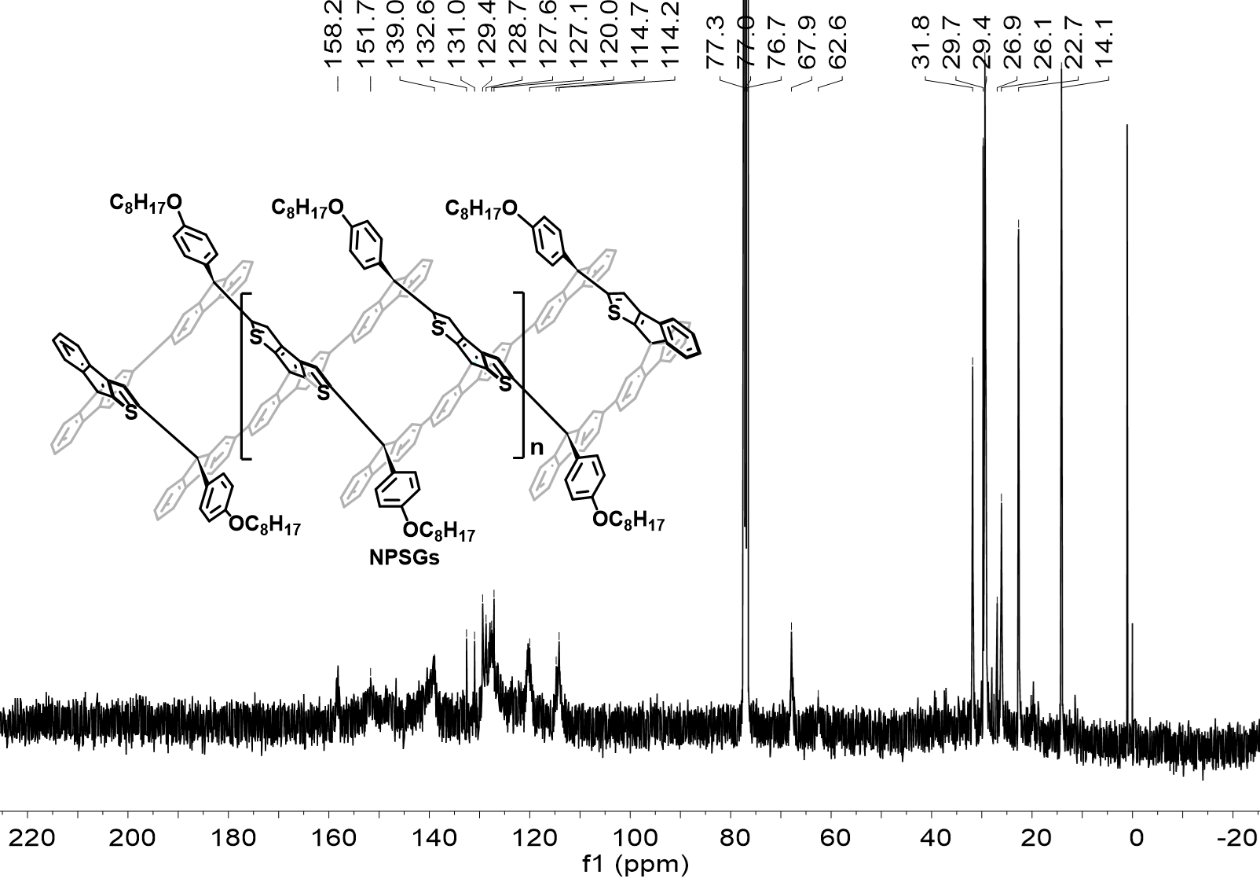


**Figure S96. ^13^C NMR spectra of NPSG**


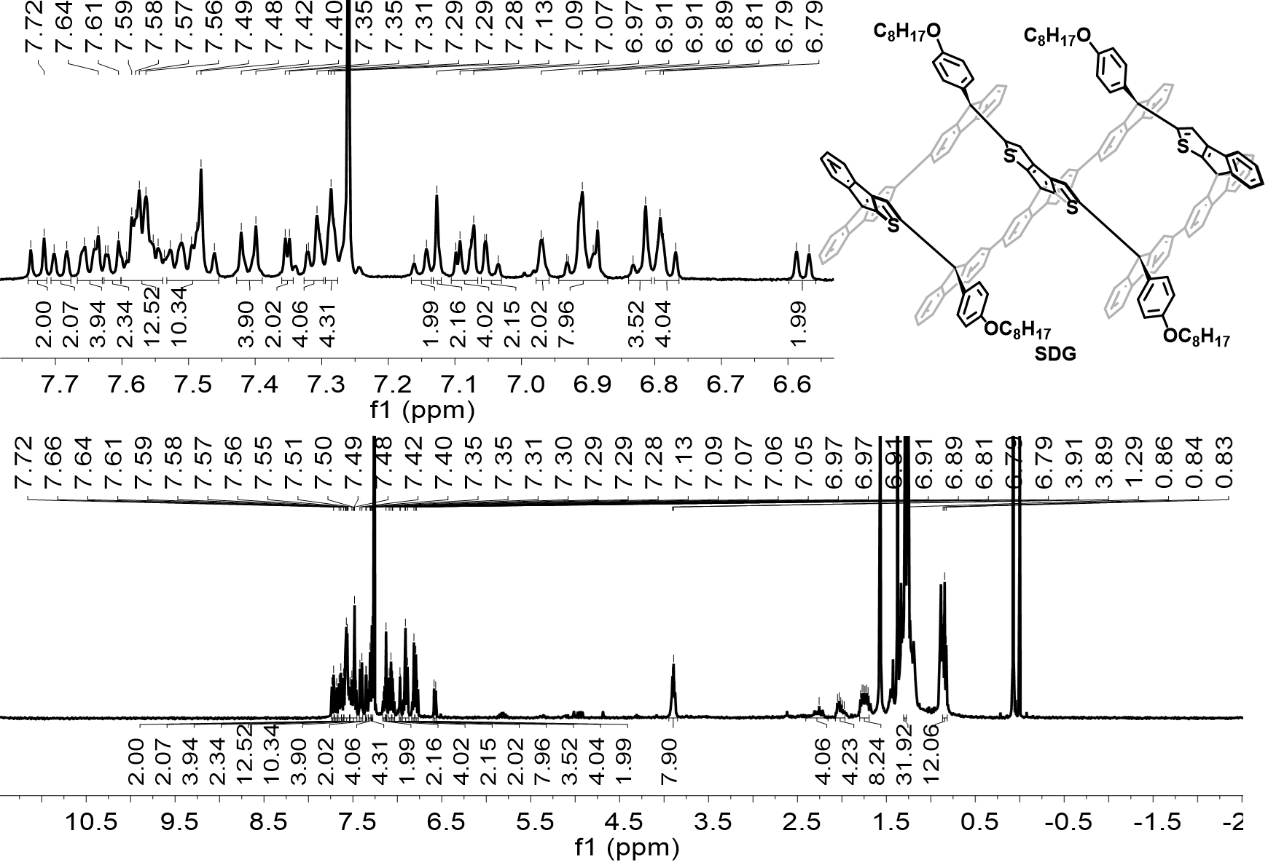


**Figure S97. ^1^H NMR spectra of SDG**


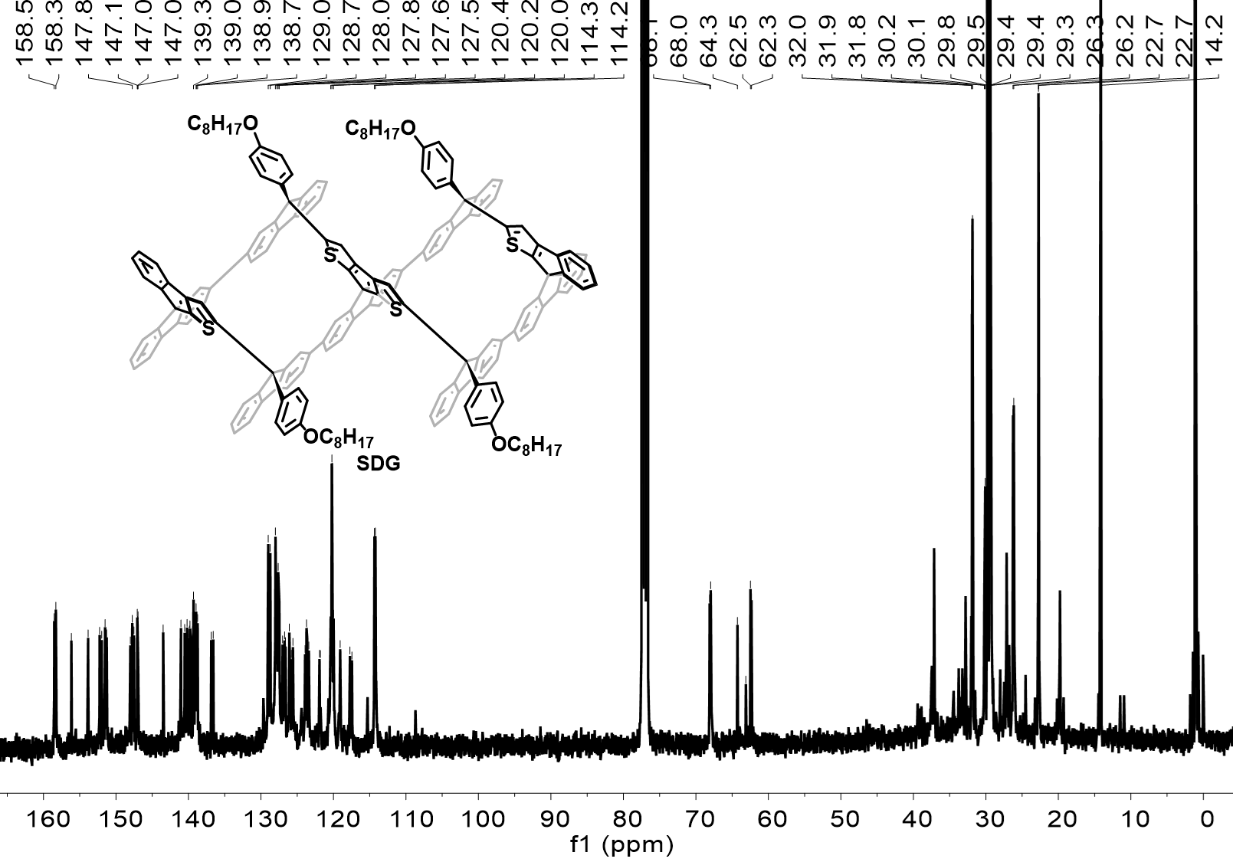


**Figure S98. ^13^C NMR spectra of SDG**

**14. References:**

[1] L.H. Xie, T. Fu, X. Y. Hou et al., “An efficient synthesis of novel spiro[[8H]indeno[2,1-b]-thiophene-8,9′-fluorene] building block for blue light-emitting materials,” *Tetrahedron Letters*, vol. 47, no. 36, pp. 6421-6424, 2006.

[2] L. Wang, G. W. Zhang, C. J. Ou et al., “Friedel–Crafts Bottom-up Synthesis of Fluorene-Based Soluble Luminescent Organic Nanogrids,” *Organic Letters*, vol. 16, no. 6, pp. 1748-1751, 2014.

[3] D. Q. Lin, Y. Wei, A. Z. Peng et al., “Stereoselective gridization and polygridization with centrosymmetric molecular packing,” *Nature Communications*, vol. 11, no. 1, pp. 1756-1766, 2020.

[4] A. P. Scott, L. Radom, “Harmonic Vibrational Frequencies:  An Evaluation of Hartree−Fock, Møller−Plesset, Quadratic Configuration Interaction, Density Functional Theory, and Semiempirical Scale Factors,” *Journal of Physical Chemistry*, vol. 100, no. 41, pp. 16502-16513, 1996.

[5] M. Radmacher, M. Fritz, H. Hansma et al., “Direct observation of enzyme activity with the atomic force microscope,” *Science*, vol. 265, no. 5178, pp. 1577-1579, 1994.

[6] H. Yin, S. H. Cheung, J. H. L. Ngai et al. “Thick-Film High-Performance Bulk-Heterojunction Solar Cells Retaining 90% PCEs of the Optimized Thin Film Cells,” *Advanced Electronic Materials*, vol. 3, no. 4, 1700007, 2017.

[7] (a) M. J. Frisch et al., Gaussian 09, revision A. 02, Gaussian, Inc., Wallingford, CT, 2009 Search PubMed; (b) C. T. Lee, W. T. Yang and R. G. Parr, “Development of the Colle-Salvetti correlation-energy formula into a functional of the electron density,” *Physical review B: Condensed matter and materials physics*, vol. 37, no. 2, pp. 785-789, 1988.

[8] C. C. Jia, J. Y. Wang, C. J. Yao et al., “Conductance Switching and Mechanisms in Single-Molecule Junctions,” *Angewandte Chemie International Edition*, vol. 52, no. 33, pp. 8666-8670, 2013.

[9] C. L. Wang, Z. B. Zhang, S. Pejic et al., “High Dielectric Constant Semiconducting Poly(3-alkylthiophene)s from Side Chain Modification with Polar Sulfinyl and Sulfonyl Groups,” *Macromolecules*, vol. 51, no. 22, pp. 9368-9381, 2018.

[10] S. Vanhee, R. Rulkens, U. Lehmann et al., “Synthesis and characterization of rigid rod poly(p-phenylenes),” *Macromolecules*, vol. 29, no. 15, pp. 5136-5142, 1996.

[11] L. H. Xie, X. Y. Hou, W. Huang, “Facile Synthesis of Complicated 9,9-Diarylfluorenes Based on BF3·Et2O-Mediated Friedel−Crafts Reaction,” *Organic Letters*, vol. 8, no. 17, pp. 3701-3704, 2006.

[12] X. J. Pan, M. W. Ishaq, A. Umair et al., “Evolution of Single Chain Conformation for Model Comb-Like Chains with Grafting Density Ranging from 0 to ∼100% in Dilute Solution,” *ACS Macro Letters*, vol. 8, no. 12, pp. 1535-1540, 2019.

[13] H. G. Chae, S. Kumar, “Rigid-rod polymeric fibers,” *Journal of Applied Polymer Science*, vol. 100, no. 1, pp. 791-802, 2006.

[14] J. B. Fleischman, “The physical properties of DNA from T2 and T4 bacteriophage,” *Journal of Molecular Biology*, vol. 2, no. 4, pp. 226-240, 1960.

[15] B. J. Bauer, M. L. Becker, V. Bajpai et al., “Measurement of Single-Wall Nanotube Dispersion by Size Exclusion Chromatography,” *Journal of Physical Chemistry C*, vol. 111, no. 48, pp. 17914-17918, 2007.

[16] M. Nagai, J. Liu, W. Huang et al., “Molecular Weight Induced Conformational Change and Its Impact on β-Phase Formation of Polydiarylfluorene in Dilute Solution,” *The Journal of Physical Chemistry C*, vol. 124, no. 11, pp. 6304-6310, 2020.

[17] M. Nagai, J. Huang, T. Zhou et al., “Effect of molecular weight on conformational characteristics of poly(3-hexyl thiophene),” *Journal of Polymer Science Part B- Polymer Physics*, vol. 55, no. 17, pp. 1273-1277, 2017.

[18] Y. B. Yao, S. D. Luo and T. Liu, “Determination of the Length, Diameter, Molecular Mass, Density and Surfactant Adsorption of SWCNTs in Dilute Dispersion by Intrinsic Viscosity, Sedimentation, and Diffusion Measurements,” *Macromolecules*, vol. 47, no. 9, pp. 3093-3100, 2014.

[19] C. Gans, J. Schnee, U. Scherf, “Viscometric determination of the statistical segment length of wormlike polymers,” *Polymer*, vol. 39, no. 17, pp. 4155-4158, 1998.

[20] T. Hokajo, K. Terao, Y. Nakamura et al., “Solution Properties of Polymacromonomers Consisting of Polystyrene V. Effect of Side Chain Length on Chain Stiffness,” *Polymer Journal*, vol. 33, no. 6, pp. 481-485, 2001.

[21] M. Jikei, M. Kakimoto, “Hyperbranched polymers: a promising new class of materials,” *Progress in Polymer Science*, vol. 26, no. 8, pp. 1233-1285, 2001.

[22] K. C. Lim, C. R. Fincher and A. J. Heeger, “Rod-to-coil transition of a conjugated polymer in solution,” *Physical Review Letters*, vol. 50, no. 24, pp. 1934-1937, 1983.

[23] W. H. Stockmayer, J. W. Kennedy, “Viscoelastic Spectrum of Free-Draining Block Copolymers,” *Macromolecules*, vol. 8, no. 3, pp. 351-355, 1975.

[24] L. Dai, “Random coils of polyisoprene in solution—a small angle x-ray scattering study,” *European Polymer Journal*, vol. 29, no. 5, pp. 645-651, 1993.

[25] M. E. Mackay, T. T. Dao, A. Tuteja et al., “Nanoscale effects leading to non-Einstein-like decrease in viscosity,” *nature materials*, vol. 2, no. 11, pp. 762-766, 2003.

[26] H. D. T.Mertens, D. I. Svergun, “Structural characterization of proteins and complexes using small-angle X-ray solution scattering,” *Journal of Structural Biology*, vol. 172, no. 1, pp. 128-141, 2010.

[27] T. J. Prosa, B. J. Bauer, E. J.Amis, “From Stars to Spheres:  A SAXS Analysis of Dilute Dendrimer Solutions,” *Macromolecules*, vol. 34, no. 14, pp. 4897-4906, 2001.

[28] G. R. Strobl, “The Physics of Polymers: Concepts for Understanding Their Structures and Behavior,” *Springer*, 2007.

[29] J. Teixeira, “Small-angle scattering by fractal systems,” *Journal of Applied Crystallography*, vol. 21, pp. 781-785, 1988.

[30] Q. Chen, C. Saltiel, S. Manickavasagam et al., “Aggregation behavior of single-walled carbon nanotubes in dilute aqueous suspension,” *Journal of Colloid and Interface Science*, vol. 280, no. 1, pp. 91-97, 2004.

[31] D. Bagchi, R. Menon, “Conformational modification of conducting polymer chains by solvents: Small-angle X-ray scattering study,” *Chemical Physics Letters*, vol. 425, no. 1-3, pp. 114-117, 2006.

[32] B. J.Bauer, E. K. Hobbie and M. L. Becker, “Small-Angle Neutron Scattering from Labeled Single-Wall Carbon Nanotubes,” *Macromolecules*, vol. 39, no. 7, pp. 2637-2642, 2006.

[33] J. H. Chen, C. S. Chang, Y. X. Chang et al., “Gelation and Its Effect on the Photophysical Behavior of Poly(9,9-dioctylfluorene-2,7-diyl) in Toluene,” *Macromolecules*, vol. 42, no. 4, pp. 1306-1314, 2009.

[34] H. Fischer, M. D. Neto, H. B. Napolitano et al., “Determination of the molecular weight of proteins in solution from a single small-angle X-ray scattering measurement on a relative scale,” *Journal of Applied Crystallography*, vol. 43, pp. 101-109, 2010.

[35] P. K. Choudhury, D. Bagchi, C. S. S. Sangeeth et al., “Modified conformation and physical properties in conducting polymers due to varying conjugation and solvent interactions,” *Journal of Materials Chemistry*, vol. 21, no. 5, pp. 1607-1614, 2011.

[36] Z. Q. Lin, N. Shi, Y. B. Li et al., “Preparation and Characterization of Polyfluorene-Based Supramolecular π-Conjugated Polymer Gels,” *Journal of Physical Chemistry C*, vol. 115, no. 11, pp. 4418-4424, 2011.

[37] A. Loubat, M. Imperor-Clerc, B. Pansu, et al., “Growth and Self-Assembly of Ultrathin Au Nanowires into Expanded Hexagonal Superlattice Studied by in Situ SAXS,” *Langmuir*, vol. 30, no. 14, pp. 4005-4012, 2014.

[38] J. K. Sprafke, D. V. Kondratuk, M. Wykes et al., “Belt-shaped π-systems: relating geometry to electronic structure in a six-porphyrin nanoring,” *journal of the american chemical society*, vol. 133, no. 13, pp. 17262-17273, 2011.

[39] X. H. Wang, S. H. Goh, Z. H. Lu et al., “Light-Scattering Characterization of Fullerene-Containing Poly(alkyl methacrylate)s in THF,” *Macromolecules*, vol. 32, no. 8, pp. 2786-2788, 1999.

[40] L. A. Feigin, D. I.Svergun, “Structure analysis by small-angle X-ray and neutron scattering,” *Springer*, 1, 1987.

[41] C. Bustamante, J. F. Marko, E. D et al., “Siggia Entropic elasticity of lambda-phage DNA,” *Science*, vol. 265, no. 5178, pp.1599-1600, 1994.

[40] Y. Nakamura, “Stiffness parameter of brush-like polymers with rod-like side chains,” *journal of chemical physics*, vol. 145, no. 1, 2016.

[41] L.Cademartiri, G. Guerin, K. J. M. Bishop et al., “Polymer-like Conformation and Growth Kinetics of Bi2S3 Nanowires,” *journal of the american chemical society*, vol. 134, no. 22, pp. 9327-9334, 2012.

[42] M. Wintermantel, M. Schmidt, Y. Tsukahara et al., “Rodlike combs,” *macromolecular rapid communications*, vol. 15, no. 3, pp. 279-284, 1994.

[43] N. Nemoto, M. Nagai, A. Koike et al., “Diffusion and Sedimentation Studies on Poly(macromonomer) in Dilute Solution,” *Macromolecules*, vol. 28, no. 11, pp. 3854-3859, 1995.

[44] P. M. Cotts, T. M. Swager, Q. Zhou, “Equilibrium flexibility of a rigid linear conjugated polymer,” *Macromolecules*, vol. 29, no. 23, pp. 7323-7328, 1996.

[45] P. Hickl, M. Ballauff, U. Scherf et al., “Characterization of a Ladder Polymer by Small-Angle X-ray and Neutron Scattering,” *Macromolecules*, vol. 30, no. 2, pp. 273-279, 1997.

[46] M. Sano, A. Kamino, J. Okamura et al., “Ring Closure of Carbon Nanotubes,” *Science*, vol. 293, no. 5533, pp.1299-1301, 2001.

[47] G. Fytas, H. G. Nothofer, U. Scherf et al., “Structure and dynamics of nondilute polyfluorene solutions,” *Macromolecules*, vol. 35, no. 2, pp. 481-488, 2002.

[48] M. Reches, E. Gazit, “Casting Metal Nanowires Within Discrete Self-Assembled Peptide Nanotubes,” *Science*, vol. 300, no. 5619, pp.625-627, 2003.

[49] L. A. Hough, M. F. Islam, P. A. Janmey et al., “ Viscoelasticity of Single Wall Carbon Nanotube Suspensions,” *Physical Review Letters*, vol. 93, no. 16, pp.168102, 2004.

[50] L.Cademartiri, G. Guerin, K. J. M. Bishop et al., “Polymer-like Conformation and Growth Kinetics of Bi2S3 Nanowires,” *Journal of the American Chemical Society*, vol. 134, no. 22, pp. 9327-9334, 2012.

[51] H. M. Chen, S. P. Meisburger, S. A. Pabit et al., “Ionic strength-dependent persistence lengths of single-stranded RNA and DNA,” *Proceedings of the National Academy of Sciences of the United States of America*, vol. 109, no. 3, pp.799-804, 2012.

[52] Y. Nakamura, “Stiffness parameter of brush-like polymers with rod-like side chains,” *journal of chemical physics*, vol. 145, no. 1, 2016.

[53] M. Saitoh, Y. Kashiwagi, M. Chigane, “Structural analysis of micrometer-long gold nanowires using a wormlike chain model and their rheological properties,” *Soft Matter*, vol. 13, no. 21, pp.3927-3935, 2017.

[54] Y. J. Kinose, K. Sakakibara, H. Ogawa et al., “Main-Chain Stiffness of Cellulosic Bottlebrushes with Polystyrene Side Chains Introduced Regioselectively at the O-6 Position,” *Macromolecules*, vol. 52, no. 22, pp. 8733-8740, 2019.

[55] P. J. Hagerman, “Flexibility of DNA,” *Annual review of biophysics and biophysical chemistry*, vol. 17, pp.265-286, 1988.

[56] R. S. Zhang, W. L. Mattice, “Evaluation of the persistence length of the rigid-rod polymers poly(benzobisoxazole) and poly(benzobisthiazole) using molecular-dynamics simulations,” *Macromolecules*, vol. 25, no. 19, pp.4937-4941, 1992.

[57] D. Hu, J. Yu, K. Wong, B. Bagchi, P. J. Rossky, P. F. Barbara, “Collapse of stiff conjugated polymers with chemical defects into ordered, cylindrical conformations,” *Nature*, vol. 405, no. 6790, pp. 1030-1033, 2000.

[58] Q. Y. Feng, Y. L. Han, M. N. Yu et al., “A robust and soluble nanopolymer based on molecular grid-based nanomonomer,” *Chinese Journal of Polymer Science*, vol. 35, no. 1, pp.87-97, 2017.

[59] M. N. Yu, J. Y. Lin, Y. X. Li et al., “Emission Enhanced and Stabilized by Stereoisomeric Strategy in Hierarchical Uniform Supramolecular Framework,” *Chem*, vol. 5, no. 9, pp.2470-2483, 2019.

[60] G. W. Zhang, Y. Wei, J. S. Wang et al., “A robust molecular unit nanogrid servicing as network nodes via molecular installing technology,” *Materials Chemistry Frontiers*, vol. 1, no. 3, pp.455-459, 2017.

[61] Y.Y. Lin, J. Y. Liu, Y. F. Bo et al., “Synthesis and Crystal Structure of Highly Strained [4]Cyclofluorene: Green-Emitting Fluorophore,” *Organic Letters*, vol. 18, no. 2, pp.172-175, 2016.

[62] D. Hertel, U. Scherf, H. Bässler, “Charge Carrier Mobility in a Ladder-Type Conjugated Polymer,” *Advanced Materials*, vol. 10, no. 14, pp. 1119, 1998.

[63] D. Hertel, H. Bässler, U. Scherf et al., “Charge carrier transport in conjugated polymers,” *Journal of Chemical Physics*, vol. 110, no. 18, pp. 9214-9222, 1999.

[64] L. Bozano, S. A. Carter, J. C. Scott et al., “Temperature- and field-dependent electron and hole mobilities in polymer light-emitting diodes,” *Applied Physics Letters*, vol. 74, no. 8, pp. 1132-1134, 1999.

[65] B. Liu, J. Y. Lin, F. Liu et al., “Highly Crystalline and Wide-Bandgap Polydiarylfluorene with β-Phase Conformation toward Stable Electroluminescence and Dual Amplified Spontaneous Emission,” *ACS Applied Materials & Interfaces*, vol. 8, no. 33, pp. 21648-21655, 2016.

[66] I. I. Fishchuk, A. K. Kadashchuk, , J. [Genoe](https://www.webofscience.com/wos/alldb/general-summary?queryJson=%5B%7B%22rowBoolean%22:null,%22rowField%22:%22AU%22,%22rowText%22:%22Genoe,%20J.%22%7D%5D&eventMode=oneClickSearch) et al., “Temperature dependence of the charge carrier mobility in disordered organic semiconductors at large carrier concentrations,” *Physical Review B*, vol. 81, no. 4, 2010.

[67] H. Yin, K. L. Chiu, P. Q. Bi et al., “Enhanced Electron Transport and Heat Transfer Boost Light Stability of Ternary Organic Photovoltaic Cells Incorporating Non-Fullerene Small Molecule and Polymer Acceptors,” *Advanced Electronic Materials*,vol. 5, no. 10, 2019.

[68] D. A. M. Egbe, S. Turk, S. Rathgeber et al., “Anthracene Based Conjugated Polymers: Correlation between π−π-Stacking Ability, Photophysical Properties, Charge Carrier Mobility, and Photovoltaic Performance,” *Macromolecules*, vol. 43, no. 3, pp. 1261-1269, 2010.

[69] A. J. Mozer, N. S. Sariciftci, A. Pivrikas et al., “Charge carrier mobility in regioregular poly(3-hexylthiophene) probed by transient conductivity techniques: A comparative study,” *Physical Review B*, vol. 71, no. 3, 2005.

[70] D. Chirvase, Z. Chiguvare, M. Knipper et al., “Temperature dependent characteristics of poly(3 hexylthiophene)-fullerene based heterojunction organic solar cells,” *Journal of Applied Physics*, vol. 93, no. 6, pp. 3376-3383, 2003.

[71] S. A. Choulis, Y. Kim, J. Nelson et al., “High ambipolar and balanced carrier mobility in regioregular poly(3-hexylthiophene),” *Applied Physics Letters*, vol. 85, no. 17, pp. 3890-3892, 2004.

[72] J. A. Röhr, X. Y. Shi, S. A. Haque et al., “Charge Transport in Spiro-OMeTAD Investigated through Space-Charge-Limited Current Measurements,” *Physical Review Applied*, vol. 9, no. 4, 2018.

[73] G. H. Meng, Y. T. Shi, X. D. Song et al., “Theoretical insight into the carrier mobility anisotropy of hole transport material Spiro-OMeTAD,” *Current Applied Physics*, vol. 17, no. 10, pp. 1316-1322, 2017.

[74] D. P. Tabor, V. A. Chiykowski, P. [Friederich](https://www.webofscience.com/wos/alldb/general-summary?queryJson=%5B%7B%22rowBoolean%22:null,%22rowField%22:%22AU%22,%22rowText%22:%22Friederich,%20Pascal%22%7D%5D&eventMode=oneClickSearch) et al. “Design rules for high mobility xanthene-based hole transport materials,” *Chemical Science*, vol. 10, no. 36, pp. 8360-8366, 2019.

[75] P. Friederich, T. Neumann, V .Rodin et al*.,* “Molecular Origin of the Charge Carrier Mobility in Small Molecule Organic Semiconductors,” *Advanced Functional Materials*, vol. 26, no. 31, pp. 5757-5763, 2016.

[76] V. Rodin, F. Symalla, V. Meded et al., “Generalized effective-medium model for the carrier mobility in amorphous organic semiconductors,” *Physical Review B*, vol. 91, no. 15, 2015.

[77] B. S. Simpkins, M. A. Mastro, C. R. Eddy, J. K. Hite, P. E. Pehrsson, “Space-charge-limited currents and trap characterization in coaxial AlGaN/GaN nanowires,” *Journal of Applied Physics*, vol. 110, no. 4, 2011.

[78] V. R. Nikitenko, H. Heil, H. v. Seggern, “Space-charge limited current in regioregular poly-3-hexyl-thiophene,” *Journal of Applied Physics*, vol. 94, no. 4, pp. 2480-2485, 2003.

[79] U. Bach, K. De Cloedt, H. Spreitzer et al., “Characterization of Hole Transport in a New Class of Spiro-Linked Oligotriphenylamine Compounds,” *Advanced Materials*, vol. 12, no. 14, pp. 1060-1063, 2000.

[80] D. Abbaszadeh, A. Kunz, G. A. H.Wetzelaer et al., “Elimination of charge carrier trapping in diluted semiconductors,” *Nature Materials*, vol. 15, no. 6, pp. 628-633, 2016.
